# Effectiveness of Recovery Strategies After Training and Competition in Endurance Athletes: An Umbrella Review

**DOI:** 10.1186/s40798-024-00724-6

**Published:** 2024-05-16

**Authors:** Shuting Li, Matthias Kempe, Michel Brink, Koen Lemmink

**Affiliations:** Department of Human Movement Sciences, University of Groningen, Groningen, The Netherlands

**Keywords:** Prevention, Research synthesis, Performance, Training, Fatigue

## Abstract

**Background:**

Recovery strategies are used to enhance performance and reduce injury risk in athletes. In previous systematic reviews, individual recovery strategies were investigated to clarify their effectiveness for mixed groups of athletes. However, the current evidence is ambiguous, and a clear overview of (training) recovery for endurance athletes is still lacking.

**Methods:**

We conducted an umbrella review based on a literature search in PubMed, Cochrane Database of Systematic Reviews, and Web of Science. Reviews published in English and before December 2022 were included. Systematic reviews and meta-analyses were eligible if they investigated the effectiveness of one or more recovery strategies compared with a placebo or control group after a training session in endurance athletes.

**Results:**

Twenty-two reviews (nine systematic reviews, three meta-analyses, and ten systematic reviews with meta-analyses included) met the inclusion criteria. In total, sixty-three studies with 1100 endurance athletes were included in our umbrella review. Out of the sixty-three studies, eight provided information on training recovery time frame for data synthesis. Among them, cryotherapy and compression garments showed positive effects, while applying massage showed no effect. In general, none of the included recovery strategies showed consistent benefits for endurance athletes.

**Conclusion:**

There is no particular recovery strategy that can be advised to enhance recovery between training sessions or competitions in endurance athletes. However, individual studies suggest that compression garments and cryotherapy are effective training recovery strategies. Further research should improve methodology and focus on the different time courses of the recovery process.

***Registration*:**

The review protocol was registered with the International Prospective Register of Systematic Reviews with the number CRD42021260509.

**Supplementary Information:**

The online version contains supplementary material available at 10.1186/s40798-024-00724-6.

## Key Points

- This is the first review that provides a global overview on the effectiveness of different (training) recovery strategies for endurance athletes.
- Research on training recovery in endurance athletes is sparse. While the use of compression garments and cold-water immersion seems to improve training recovery, none of the included recovery strategies have shown consistent recovery benefits in general.
- Endurance athletes seem to react differently to the application of specific strategies compared to, e.g., team sports athletes. Therefore, future research (reviews) should focus on endurance athletes instead of mixed groups of athletes.

**Supplementary Information:**

The online version contains supplementary material available at 10.1186/s40798-024-00724-6.

## Background

Performance increase in (endurance) sports is a result of continuous, consecutive training sessions [1]. To optimize the effect of these sessions, sufficient recovery is needed to be able to adapt a training stimulus [2–4]. An imbalance between the induced stress of training and the subsequent recovery possibly affects the rate of post-exercise glycogen synthesis/stores, inflammation processes, and metabolic disturbances [2]. Over time, this could accumulate into serious health problems like injuries, illnesses, and non-functional overreaching [5, 6]. It is therefore assumed that post-exercise recovery strategies are vital in improving the time course of recovery between (training) sessions [2, 7]. This is especially vital for athletes with exceptionally high training loads, such as endurance athletes. For instance, male marathon runners typically cover an average of 150 to 260 km per week [8]. Marathon running had the highest metabolic equivalent (MET) hours at 13.3, followed by triathlons and speed skating at the same level, rowing at 12.0, cycling at 10, and swimming at 9.8. In comparison, non-endurance athletes and team sports like bodybuilding scored 6 in MET hours, basketball 8.0, and soccer 10.0. Given their demand for high-volume and intense training, optimizing recovery is imperative for endurance athletes [9]. Therefore, they will be the focus point of this umbrella review.

Due to its multifaceted nature, recovery can be seen as an umbrella term covering multiple modalities. To account for this, there is no one marker for recovery, but instead a variety of markers. Performance tests (e.g., countermovement jumps and aerobic tests [10]) are often used to monitor recovery and performance in athletes. Thereby such tests provide specific markers that are associated with fatigue and recovery. They can be categorized, for example, as biomechanical (e.g. step length during a treadmill test) or biochemical markers (e.g. creatine kinase and lactate [11]). In addition, physiological markers (e.g., VO_2_max [11] or heart rate [12, 13]) can determine the underlying physiology of the post-exercise/competition recovery process. Finally, psychological markers that capture the perception of an athlete have been shown to be highly relevant for monitoring training responses. These markers are often assessed via self-reported measures (e.g., athlete recovery stress questionnaires [3] or mood [14]). Given this variety of markers, it is hard to judge if a recovery strategy is efficient as it could show positive results for one marker while there are no benefits for others.

Recovery strategies can be distinguished by their type of application. Using this approach, Kellmann et al. (2018) classified recovery strategies into passive, active, and proactive recovery [13, 15]. Passive recovery is seen as the process in which the body recovers through external stimulations, such as massage, compression garments, temperature-based strategies, or nutrition supplements. In contrast, active recovery involves voluntary activities like jogging, walking, and stretching. Proactive recovery involves self-initiated and social activities chosen by the individual and catered to the individual’s needs, for example, breathing techniques, social activities [13] or improving psychological resilience in athletes [16]. We will include all three categories in this umbrella review. Furthermore, we distinguish recovery strategies from rest within this umbrella review. Thereby, we see recovery, in line with the definition by Kellmann et al. (2018), as an additional provided stimulus, whereas we see rest as inactivity (e.g. standing still or sitting) or not changing an athlete’s normal daily routine.

Recovery strategies can also be classified based on the timing of application into immediate, short-term, and training recovery [4]. Immediate recovery refers to recovery occurring between short time movements, for instance, an alternating runner’s footstep. Short-term recovery includes strategies used between training sets, like two sets of sprints or a timeout in team games. In contrast, training recovery focuses on the time between consecutive training sessions or competitions. As most exercise-induced adaptations occur during the recovery period [4], training recovery plays a vital role in the adaptation process, e.g., restoring muscle and liver glycogen [17]. While some earlier reviews reported the effectiveness of recovery strategies in terms of timing (varying from immediately after the exercise to 120 h after), there is no systematic analysis of effectiveness in terms of training recovery. Hence, we will assess the effectiveness of different recovery strategies on a training recovery timeframe (8–24 h) in this review. In addition, we will evaluate the general effectiveness of various recovery strategies in endurance athletes by type of application (passive, active, and proactive).

Although a recent questionnaire study by Braun-Trocchio et al. (2022) [18] provided an overview of which recovery strategies are used by endurance athletes, a cohesive overview on ways to enhance training recovery in endurance athletes is still lacking. Thus, in this umbrella review (UR), we focus on training recovery to provide athletes and practitioners with evidence-based recommendations and guidelines to improve recovery, optimize the training process, and in turn improve performance.

## Methods

This UR addressed all the PRISMA statement’s recommendations (see Additional file 1: Table S1) [19]. The review protocol was registered with the International Prospective Register of Systematic Reviews (PROSPERO ID CRD42021260509).

### Search Strategy

The literature search was carried out by one reviewer (SL) in December 2022 and focused on three databases: PubMed, Web of Science, and Cochrane Database of Systematic Reviews. Relevant search terms were connected with Boolean operators by keywords of recovery and endurance to search for eligible reviews. The exact search terms are included in Additional file 2: Table S2.

The lead author searched the reference lists for eligible studies and topical review articles (SL). Two reviewers (SL and MK) independently selected and extracted the data using the online application Rayyan (Rayyan Systems Inc., Cambridge, MA, USA) [20]. Two reviewers (SL and MK) scanned the titles and abstracts of all included studies. All authors discussed conflicts in the extracted data to reach an agreement on the included reviews; one reviewer (SL) removed the duplicates.

### Selection Criteria

Systematic reviews (SR), meta-analyses (MA), and systematic reviews with meta-analyses (SRMA) were included if they focused on observational and experimental studies that explored the effects of recovery strategies in endurance athletes. Recovery, in this UR, was seen as a process of reducing exercise-induced fatigue; therefore, reviews focusing on recovery from injuries, wounds, or similar were excluded.

#### Participants

Adult male or female athletes at a recreational (e.g., trained, club), competitive (e.g., actively training for sports competition), or elite (e.g., highly trained, international) level with a focus on endurance sports in their training regime were eligible for this umbrella review. This is equivalent to tier 1 to tier 5 of the participant categorization framework of colleagues [21]. Endurance sports were seen as activities by individuals (e.g., non-team athletes) in which key muscles were used at submaximal intensity for prolonged periods.

#### Interventions

We conducted a targeted search for all recovery strategies, including massage, active recovery, cryotherapy, contrast temperature water immersion, compression garments, stretching, and electrostimulation, but not limited to those mentioned by Barnett 2006 [2]. Then, all strategies included were classified into three categories based on Kellmann 2022 [15].

### Evaluation of the Methodological Quality

Two reviewers (SL, MK) examined the quality of the reviews using the A MeaSurement Tool to Assess systematic Reviews 2 (AMSTAR 2) tool [22]. The AMSTAR 2 checklist is a dependable and effective tool for determining bias in reviews. All reviews were evaluated using the standard AMSTAR 2 with 16 items (detail items see Additional file 3: File S3). The modified AMSTAR 2 with 13 items was used if the article was an SR. The score for each question was ‘yes (1 point)’, ‘no (0 point)’, ‘partial yes (0.5 point)’, and ‘N/A (not applicable)’. According to the total score, reviews were divided into four quality groups, namely high (≥ 75%), moderate (≥ 50%), low (< 50%), and critically low (< 25%). To ensure the quality of included studies, the reviews with a ‘critically low’ overall confidence (OC) rating were eliminated from further analyses.

### Data Extraction and Analysis

Two reviewers (SL and MK) extracted data in three steps using Microsoft Excel (Microsoft Excel for Mac Version 16.69) to organize the data. Firstly, we presented the characteristics of the selected reviews as follows: authors, recovery type, number of included studies, number of participants, heterogeneity (I^2^), confidence intervals (95% CI) around the effect size, *p* value, as well as the main finding. As all reviews also included non-endurance athletes, individual studies focusing only on endurance athletes were extracted from the selected reviews in a second step. For further inclusion, those studies needed to have a clear control or placebo group. To control for that, all studies included in the reviews were checked and those that met these criteria were used for further analysis. As some studies were repeated in different reviews, the information in the study with the higher AMSTAR 2 rating was used for further analysis. For these included studies, key information like the number and types of participants, type of exercise, intervention exercise (intensity), recovery type, comparison group, and main outcomes were extracted. The extracted data for each recovery strategy are presented in Additional file 4: Table S4. In the final and third steps, we analyzed the time courses of the different recovery strategies 8 to 24 h after training or competition with metadata of reviews.

### Data Synthesis

The outcomes of different recovery strategies were evaluated in terms of athletic performance and physiological and self-perceived outcomes. Outcome measures were chosen based on the frequency of occurrence in the included studies. The most frequently used outcome variables were selected for further analysis. The results were assessed using criteria from Born et al. (2013) and Engle et al. (2016) [23, 24] and divided into four categories: positive effects (↑), negative effects (↓), no effect (↔), or contradictory effects (↑↓) of positive as well as negative effects (see Additional file 4: Table S4).

## Results

### Search Results

**Fig. 1:**
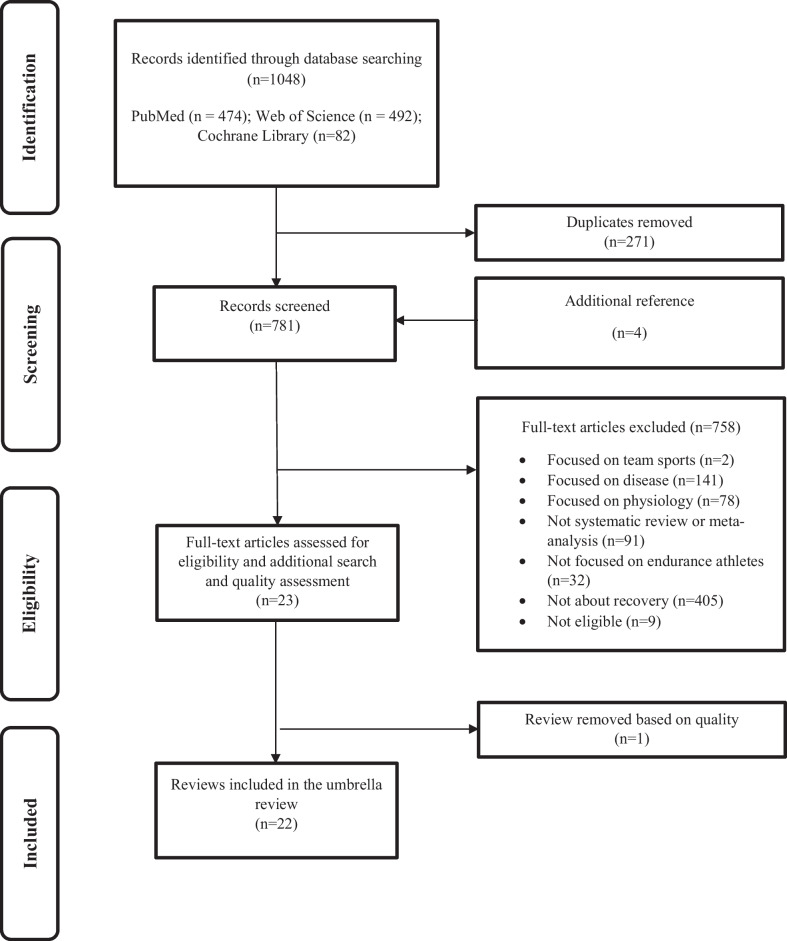
Flow chart of the review search and selection process

**Fig. 2:**
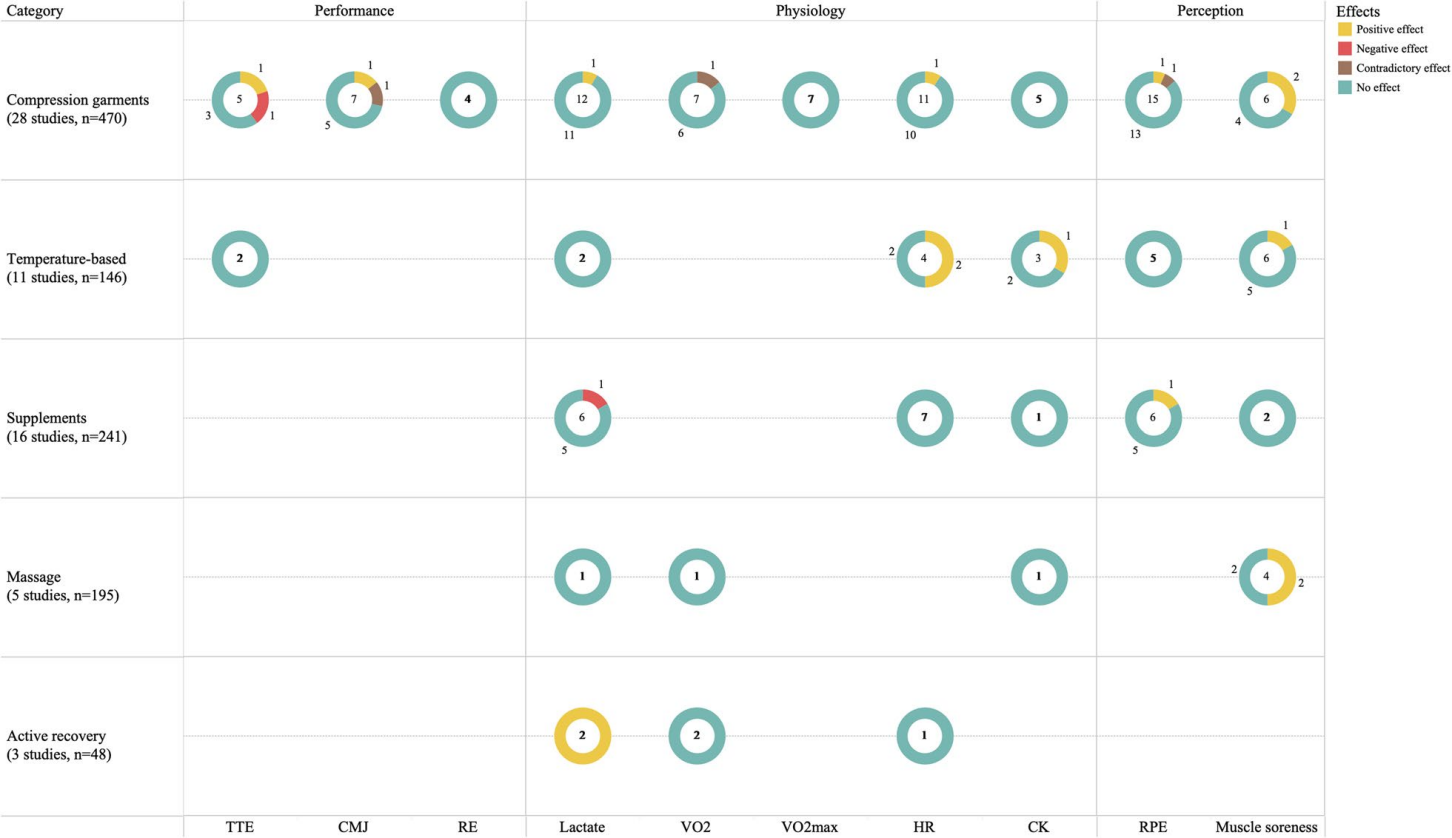
Effect in endurance athlete

Our literature search yielded twenty-two reviews, including nine SR, three MA, and ten SRMA (see Fig. 1). An overview of all 1030 excluded reviews can be found in Additional file 5: Table S5. All included reviews (see Table 1) were published in English before December 2022. Ten reviews were published from 2012 to 2018, and 12 were published between 2019 to 2022. The 22 included reviews contained 547 studies with 9327 participants in various sports. All reviews were categorized as either focusing on passive (nineteen reviews, 460 studies, 8097 participants, see "Effectiveness of Passive Recovery Strategies" section), active (two reviews, 75 studies, 1103 participants, see "Effectiveness of Active Recovery Strategies" section), or proactive recovery (one review, 12 studies, 127 participants, see "Effectiveness of Proactive Recovery Strategies" section) strategies. A complete overview of all included recovery strategies can be found in Table 1. Further details focusing on endurance athletes are presented in "Effects in Endurance Athletes", "Effect in Endurance Athletes", "Effect In Endurance Athletes" sections, and Fig. 2.

**Table 1:** Characteristics and results of the included reviews

Review	Type	Studies included within review/N	Included endurance studies/N	Outcomes	
				Heterogeneity (I^2^)	Main results
Alcantara et al. [25]	Pas^1,a^	11/194	1/8	NR	*No quantitative pooling*: The effects of cow’s milk consumption on exercise performance and muscle function recovery were controversies and deprivation
Amiri et al. [26]	Pas^1,c^	12 /130	8/82	TTE NR Serum lactate I^2^ = 0, *p* = 0.661	*Pooled results*: There was no effect of CM consumption on TTE, RPE, HR, serum lactate, and CK compared to placebo or other sports drinks (*p* > 0.05). *Subgroup analysis*: CM increased TTE (MD = 0.78 min; 95% CI 0.27 to 1.29; *p* = 0.003) and a significant decrease in serum lactate compared to placebo (MD = − 1.2 mmol/L; 95% CI − 2.06 to − 0.34; *P* = 0.006)
Ammar et al. [27]	Pas^1,a^	11/274	5/112	NR	*No quantitative pooling*: POM can potentially improve sports performance (endurance and strength), in antioxidant and anti-inflammatory effects before and after exercise, enhance cardiovascular response and accelerate recovery from high-intensity training
Bleakley et al. [28]	Pas^3,c^	17/366	3/29	Muscle soreness 1 h, I^2^ = 2%, *P* = 0.41 24 h, I^2^ = 64%, *P* = 0.03 48 h, I^2^ = 57, *P* = 0.02	*Pooled results*: Muscle soreness showed statistically significant effects in favour of CWI after exercise at 24 h (SMD = − 0.55; 95% CI − 0.84 to − 0.27; 10 trials), 48 h (SMD = − 0.66; 95% CI − 0.97 to − 0.35; 8 trials), 72 h (SMD = − 0.93; 95% CI − 1.36 to − 0.51; 4 trials) and 96 h (SMD = − 0.58; 95% CI − 1.00 to − 0.16; 5 trials) follow-ups
Brown et al. [29]	Pas^4,b^	23/348	8/132	Strength recovery I^2^ = 64%, *p* < 0.001 Resistance exercise I^2^ = 79%, *p* < 0.001 Metabolic I^2^ = 0%, *p* = 0.58	*Pooled results*: The greatest benefit from CG is the recovery of strength (ES = 0.62; 95% CI 0.39 to 0.84; *p* < 0.001) from 2 to 8 h ES = 1.14; 95% CI 0.72 to 1.56; *p* < 0.001) and 24 h (ES = 1.03; 95% CI 0.48 to 1.57; *p* < 0.001). Recovery with CG showed the greatest, very likely benefit at the 24 h (ES = 1.33; 95% 0.80 to 1.85; *p* < 0.001) time point after resistance exercise (ES = 0.49; 95% CI 0.37 to 0.61; *p* < 0.001). Recovery from metabolic exercise with CG improved cycling performance at 24 h (ES = 1.05; 95% CI 0.25 to 1.85; *p* = 0.01). In general, CG was most effective for long-term recovery, especially in 24 h recovery after training
Costello et al. [30]	Pas^3,c^	4/64	1/9	Muscle soreness 1 h, I^2^ = 0%, *p* = 0.45 24 h, I^2^ = 64%, *p* = 0.06 48 h, I^2^ = 53%, *p* = 0.12 72 h, I^2^ = 87%, *p* = 0.005	*Pooled results*: There is insufficient evidence to determine whether utilized WBC reduces muscle soreness (pain at rest, VAS) compared to the control group. However, some evidence supports that WBC reduces muscle soreness at 1 h (SMD = − 0.77; 95% CI − 1.42 to − 0.12; *p* = 0.02; n = 20, 2 studies), 24 h (SMD = − 0.57, 95% CI − 1.48 to 0.33; *p* = 0.21; n = 38, 3 studies), 48 h (SMD = − 0.58, 95% CI − 1.37 to 0.21; *p* = 0.15; n = 38, 3 studies), and 72 h (SMD = − 0.65, 95% CI − 2.54 to 1.24; *p* = 0.50; n = 29, 2 studies) post-exercise
Davis et al. [31]	Pas^2,c^	29/1012	5/204	Flexibility I^2^ = 90% DOMS I^2^ = 86%	*Pooled results*: No evidence was found that massage improved the measures of strength, jump, sprint, endurance, or fatigue, but massage may improve flexibility (SMD = 1.07; 95% CI 0.21 to 1.93; *p* = 0.01; n = 246; studies = 7) and DOMS (SMD = 1.13; 95% CI 0.44 to 1.82; n = 311; *p* < 0.005; studies = 10) to some extent
Engel et al. [24]	Pas^4,a^	32/494	24/361	NR	*No quantitative pooling*: By wearing CG, runners can improve running economy, biomechanical variables, perception, muscle temperature, and variables related to endurance performance (i.e., TTE). Wearing CG can also result in less muscle pain, injury, and inflammation during recovery
Hendricks et al. [32]	Act^6,a^	49/632	4/74	NR	*No quantitative pooling*: FR has been shown to decrease muscle stiffness and DOMS and should be combined with dynamic stretching and an active warm-up before training. FR improves ROM and PPT. To achieve maximum flexibility, FR should be used for at least 90 to 120 s
Kloby Nielsen et al. [33]	Pas^1,c^	43/326	22/231	TTE I^2^ = 33%, *p* = 0.06 TT I^2^ = 29%, *p* = 0.01 ≥ 8 h I^2^ = 5%, *p* = 0.35	*Pooled results*: When ingested CHO-PRO, a significant overall effect on TTE (MD = 3.62; 95% CI 0.44 to 6.79; *p* = 0.03) and TT (MD = − 1.50; 95% CI − 2.37 to − 0.63; *p* = 0.0007) performance compared to ingested CHO only. Subgroup analysis showed that long-term recovery (i.e., 8 h) consumed CHO-PRO significantly enhanced TTE compared to CHO only (MD = 10.59; 95% CI 4.18 to 17.01; *p* = 0.001); however, no effect was observed when less than 8 h
Lakićević [34]	Pro^7,a^	12/127	3/31	NR	*No quantitative pooling*: Alcohol consumption after resistance exercise does not affect biological, physical measures, and muscle function. However, if alcohol is consumed consistently during recovery this can lead to increased cortisol levels, decreased testosterone levels, and lower muscle protein synthesis rates, resulting in compromised long-term muscle adaptation
Loureiro et al. [35]	Pas^1,a^	9/89	8/76	NR	*No quantitative pooling*: Milk has no advantage over a combination of carbohydrates and protein in terms of muscle glycogen recovery and subsequent exercise performance. However, a milk drink with sufficient carbohydrate additions, such as chocolate milk, may be an option to improve performance as described above
Malta et al. [36]	Pas^3,c^	8/470	2/43	NR	*Pooled results*: Use of CWI has harmful effects on resistance training adaptations which include one-repetition maximum, maximum isometric strength, and strength endurance performance (SMD = − 0.60; 95% CI 0.87 to − 0.33; *p* < 0.0001), as well as on ballistic efforts performance (SMD = − 0.61; 95% CI − 1.11 to − 0.11; *p* = 0.02). On the other hand, selected studies verified no effect of CWI associated with endurance training on time-trial (mean power), maximal aerobic power in graded exercise test performance (SMD = − 0.07; 95% CI − 0.54 to 0.53; *p* = 0.71), or time-trial performance (duration) (SMD = 0.00; 95% CI − 0.58 to 0.58; *p* = 1.00)
McCartney et al. [37]	Pas^1,c^	67/745	25/271	Mean power output (CHO + W vs. W) I^2^ = 43.9% Mean power output (PRO + CHO + W vs. W) I^2^ = 72.9%	*Pooled results*: Ingesting CHO + W (102 ± 50 g CHO; 0.8 ± 0.6 g·CHO kg^−1^·h^−1^) improved exercise performance compared with W (1.6 ± 0.7 L) in mean power output (CHO + W vs. W, MD = 4.97; 95% CI 3.2 to 4.7; *p* = 0.000; n = 486). The enhancement was reduced when participants were ‘Fed’ (having a meal 2–4 h before the initial session) compared to being ‘Fasted’ (*p* = 0.012). Ingesting PRO + CHO + W (35 ± 26 g PRO; 0.5 ± 0.4 g PRO kg^−1^) did not have a significant impact on exercise performance compared to CHO + W (115 ± 61 g CHO; 0.6 ± 0.3 g CHO·kg body mass^−1^ h^−1^;1.2 ± 0.6 L) in mean power output (PRO + CHO + W vs. CHO + W, MD = 4.97; 95% CI − 0.5 to 1.6; *p* = 0.31; n = 125) CHO (and water) intake should be prioritized during and/or after the initial exercise session to enhance performance in subsequent tasks involving endurance and/or anaerobic activity. Protein intake is unlikely to be beneficial or detrimental to subsequent endurance exercise performance
Moore et al. [38]	Pas^3,c^	52/1191	3/51	Muscular power 24 h, I^2^ = 0% 24 h HIIT, I^2^ = 74.6% CK 24 h, I^2^ = 58.6% Muscle soreness 24 h, I^2^ = 77.4% Perceived recovery 24 h HIIT, I^2^ = 34.3%	*Pooled results*: CWI improved the recovery of muscular power 24 h after eccentric exercise (SMD = 0.34; 95% CI 0.06 to − 0.62; *p* = 0.018) and after high-intensity exercise (SMD = 0.22; 95% CI 0.004 to − 0.43; *p* = 0.046), and reduced serum CK (SMD = − 0.85; 95% CI − 1.61 to 0.08; *p* = 0.030) 24 h after high-intensity exercise. CWI also improved muscle soreness (SMD = − 0.89; 95% CI − 1.48 to 0.29; *p* = 0.003) and perceived feelings of recovery (SMD = 0.66; 95% CI 0.29 to 1.03; *p* = 0.001) 24 h after high-intensity exercise
Mota et al. [39]	Pas^4,a^	21/411	15/324	NR	*No quantitative pooling*: Wearing below-knee CS after exercise has been shown to increase actual performance in a few studies. On the other hand, wearing CS may benefit measures of lower muscle fatigue and muscle soreness several hours later (e.g., 48 h)
Murray and Cardinale [40]	Pas^3,c^	17/221	1/10	Subjective I^2^ = 72.51%, *p* = 0.000	*Pooled results*: The effects of CWI on young athletes appear to be minimal or non-existent in the acute phase or the days following exercise (i.e., > 96 h). The current literature provides only a small number of studies describing acute and chronic responses to CWI and CWT in adolescent athletes. In adolescent athletes, the overall effect size of CWI is negligible. In terms of acute outcomes, the only significant benefit appears to be in subjective outcome measures (ES = 0.41; 95% CI − 0.12 to 0.94). Overall, it is difficult to draw clear conclusions
Ortiz et al. [41]	Act^5,a^	26/471	8/104	NR	*No quantitative pooling*: Active recovery results were generally inconsistent, making it difficult to draw particular conclusions. The review concludes that 6- to 10-min active recovery treatments had a persistent favorable effect on performance. The data are unclear about the optimal intensity of AR sessions; nevertheless, blood lactate clearance appears to be an unreliable indicator of recovery. According to the review, active recovery seems to have beneficial psychological effects
Poppendieck et al. [42]	Pas^3,b^	21/216	2/23	NR	*Pooled results*: All studies determined the effect of cooling on performance and calculated the effect size (*g*). The effect sizes from highest to lowest, were sprint performance (2.6%, *g* = 0.69; 95% CI 0.48 to 0.90; n = 186), endurance parameters of time trials (2.6%, *g* = 0.19; 95% CI − 0.09 to 0.47; n = 100), jump (3.0%,* g* = 0.15; 95% CI − 0.07 to 0.38; n = 157), and strength (1.8%,* g* = 0.10; 95% CI − 0.07 to 0.27; n = 267). On average, cooling had a negligible impact on recovery in trained athletes (2.4%, *g* = 0.28). This effect was most significant when evaluating performance 96 h after exercise (4.3%, *g* = 1.03). However, some studies contradict the finding that cooling produces beneficial effects
Poppendieck et al. [43]	Pas^2,b^	22/270	3/40	NR	*Pooled results*: Massage has a minor and mostly unknown influence on sports performance recovery. Massage seems most effective for short-term recovery periods of less than 10 min. Short recovery times of up to 10 min (+ 7.9%, *g* = 0.45) had a bigger impact than longer recovery periods (+ 2.4%, *g* = 0.08). Massages lasting less than 12 min (+ 1.0%, g = 0.06) had a higher effect (+ 6.6%, g = 0.34) than massages lasting more than 12 min (+ 1.0%, g = 0.06). In addition, massage is more effective after high-intensity mixed exercise (+ 14.4%, *g* = 0.61), but the effects were reduced after strength (+ 3.9%, *g* = 0.18) and endurance (+ 1.3%, *g* = 0.12) exercise. In addition, untrained athletes benefited more from massage (+ 2.3%, *g* = 0.17) than trained athletes (+ 6.5%, *g* = 0.23)
Ranchordas et al. [44]	Pas^1,c^	50/1089	12/261	Muscle soreness 6 h, I^2^ = 53%, *P* = 0 24 h, I^2^ = 5%, *P* = 0.39 48 h, I^2^ = 47%, *P* = 0 72 h, I^2^ = 27%, *P* = 0.1 96 h, I^2^ = 31%, *P* = 0.11	*Pooled results*: Following DOMS, antioxidants did not result in clinically relevant reductions in muscle soreness at 6- (SMD = − 0.30; 95% CI − 0.56 to − 0.04; *p* = 0.03; n = 525; studies = 21), 24- (SMD = − 0.13; 95% CI − 0.27 to − 0.00; *p* = 0.05; n = 936; studies = 41), 48- (SMD = − 0.24; 95% CI − 0.42 to − 0.07; *p* = 0.01; n = 1047; studies = 45), 72- (SMD = − 0.19, 95% CI − 0.38 to − 0.00; *p* = 0.04; n = 657; studies = 28), and 96-h (SMD = − 0.05; 95% CI − 0.29 to 0.19; *p* = 0.68; n = 436; studies = 17) post-exercise. There is no evidence of subjective recovery and only limited evidence of the adverse effects of antioxidant supplements
Suhett et al. [45]	Pas^1,a^	11/197	2/58	NR	*No quantitative pooling*: Most studies have demonstrated that curcumin supplementation benefits athletes. Curcumin supplementation reduced inflammation, oxidative stress, pain, and muscle damage, enhanced recovery, and muscular performance, improved psychological and physiological (thermal and cardiovascular) responses during training, and improved gastrointestinal function

Act, active recovery strategies; CG, compression garments; CHO, carbohydrates; CI, confidence interval; CK, creatine kinase; CM, chocolate milk; CS, compression stockings; CWI, cold-water immersion; CWT, contrast water therapy; DOMS, delayed onset muscle soreness; FR foam roller; g effect sizes (Hedges’ g); HIIT, high-intensity interval training; HR, heart rate; MD, mean difference; NR, not reported; Pas, passive recovery strategies; POM, pomegranate; Pro, proactive recovery strategies; PRO, protein; PPT, pressure pain threshold; ROM, range of motion; RPE, rate of perceived exertion; SMD, standardised mean difference; TT, time trial; TTE, time to exhaustion; VAS, visual analogue scale; W, water; WBC, whole-body cryotherapy

^a^Systematic review; ^b^meta-analysis; ^c^systematic review and meta-analysis; ^1^supplements; ^2^massage; ^3^cryotherapy; ^4^compression garments; ^5^active recovery; ^6^foam roller; ^7^alcohol

### Methodological Quality

The methodological quality of included reviews is presented in Table 2. The results of the AMSTAR 2 ranged from ‘critically low’ to ‘high’ in the OC ratings. Three reviews received ‘high’ level OC rates; eleven were rated ‘moderate’, and eight were rated ‘low’. One review was excluded due to a critically low AMSTAR 2 quality. This means that 22 reviews were considered for further analysis of the different recovery strategies.

**Table 2:** Results of the AMSTAR 2 methodological quality evaluation of the included meta-analysis

	AMSTAR 2 items																%	OC
Review	1	2	3	4	5	6	7	8	9	10	11	12	13	14	15	16		
Costello et al. [30]	1	0.5	1	1	1	1	1	1	1	1	1	1	1	1	0	1	90.63	High
Bleakley et al. [28]	1	0.5	0	1	1	1	1	1	1	0	1	1	1	1	0	1	78.13	High
Amiri et al. [26]	0	1	0	0.5	1	1	1	1	1	0	1	1	1	1	1	1	78.13	High
McCartney et al. [37]	1	0.5	0	0.5	1	1	0	0.5	1	0	1	1	1	1	1	1	71.88	Moderate
Alcantara et al. [25]	1	1	0	0.5	1	1	0	0.5	1	0	N/A	N/A	1	1	N/A	1	69.23	Moderate
Hendricks et al. [32]	1	1	1	0.5	1	1	0	0.5	1	0	N/A	N/A	0	1	N/A	1	69.23	Moderate
Kloby Nielsen et al. [33]	1	0	0	0.5	1	1	0	0.5	1	0	1	1	1	1	1	1	68.75	Moderate
Brown et al. [29]	1	0	0	0.5	0	0	0	0.5	1	0	1	1	1	1	1	1	56.25	Moderate
Lakićević [34]	1	0	0	0.5	1	1	0	0.5	1	0	N/A	N/A	1	0	N/A	1	53.85	Moderate
Ranchordas et al. [44]	1	0	0	0	0	1	0	0.5	1	1	0	1	1	1	0	1	53.13	Moderate
Loureiro et al. [35]	1	0	0	1	1	1	0.5	1	0	0	N/A	N/A	0	0	N/A	1	50.00	Moderate
Malta et al. [36]	1	0.5	0	0.5	1	1	0	0	0	1	0	1	1	0	0	1	50.00	Moderate
Moore et al. [38]	1	1	0	0.5	1	1	0	0.5	1	0	1	0	0	0	0	1	50.00	Moderate
Suhett et al. [45]	1	0.5	0	0.5	1	0	0	0.5	1	0	N/A	N/A	1	0	N/A	1	50.00	Moderate
Murray and Cardinale [40]	1	0	0	0	0	1	0	0.5	1	0	1	0	0	1	1	1	46.88	Low
Ammar et al. [27]	1	0	0	0.5	1	1	0	0.5	1	0	N/A	N/A	0	0	N/A	1	46.15	Low
Ortiz et al. [41]	1	0	0	0.5	1	0	0	0.5	1	0	N/A	N/A	1	0	N/A	1	46.15	Low
Davis et al. [31]	1	0	0	0	1	1	0	0.5	0	0	1	0	0	1	0	1	40.63	Low
Poppendieck et al. [43]	1	0	0	0.5	0	0	0	1	1	0	0	1	1	0	0	1	40.63	Low
Poppendieck et al. [42]	1	0	0	0	1	0	0	1	1	0	0	0	0	0	0	1	31.25	Low
Mota et al. [39]	1	0	0	0	1	0	0	0.5	0	0	N/A	N/A	0	0	N/A	1	26.92	Low
Engel et al. [24]	1	0	0	0.5	0	1	0	0.5	0	0	0	0	0	0	0	1	25.00	Low
Born et al. [23]	0	0	0	0	0	0	0	0	0	0	1	0	0	0	0	1	18.75	Critically low

N/A, not applicable; OC, overall confidence

One item of the AMSTAR 2 was reported more rigorously than others. All reviews disclosed potential conflicts of interest (item 16). Except for the critically low review [23], only one review did not report the basic PICOT format (item 1) [26]. Moreover, only one study did not report adequate details of the included reviews (item 8) [36]. Furthermore, the majority of the reviews (17 out of 23) reported the risk of bias (RoB) in individual reviews (item 9), which is considered to be one of the essential items in the AMSTAR 2 tool.

Most reviews neglected the following three general methodological items: 2 out of 23 reviews explained the design of the selected reviews (item 3), 4 out of 23 reviews listed the excluded reviews and provided justification for their exclusion (item 7), and 2 out of 23 reviews disclosed funding for the source reviews (item 10).

In addition, 4 out of 23 reviews reported that the research protocol was developed prior to the review and reported deviations from the protocol (item 2) [25, 26, 32, 38]. Therefore, most included reviews were not registered on websites such as The International Prospective Register of Systematic Reviews (PROSPERO) or Cochrane Central Register of Controlled Trials (CENTRAL).

### Characteristics of Included Studies

Based on the research questions, we mainly focused on outcomes for endurance athletes. In total, 165 studies with 2473 participants studying endurance athletes were extracted from the 22 reviews. Overall, after removing the studies that had mixed comparison groups (multiple interventions or studies that did not compare with the placebo/control group) and duplicated studies, sixty-three studies (1100 participants: 835 male, 120 female, 145 sex non-mentioned) were assessed for eligibility, which utilized passive recovery (60 studies) and active recovery (3 studies). None of the included studies investigated proactive recovery strategies for endurance athletes. The included reviews consisted of various sports; based on the research questions, we mainly focused on outcomes for endurance athletes (such as cyclists, runners, climbers, and triathletes [for an overview see Additional file 4: Table S4]).

Pre- recovery exercises were generally in line with the athlete’s sport, meaning that, for example, runners mostly performed a running activity. The pre-recovery exercise intensities varied considerably between sports and studies. For cycling, they ranged from sprinting to two hours of cycling between 59 and 80% of VO_2_max. For running, both road (marathon) or lab (treadmill) runs at different intensity levels (3 × 30 s sprints for the marathon or 120 min at 60% of VO_2_max to TTE test at 90% of VO_2_max) were used, while in swimming, only maximal intensity swims were studied. Additionally, full or half triathlon competitions were used.

In terms of study design, there were major differences in the assessment of different recovery strategies. Studies applying cryotherapy and massage typically used a pre-post design, where a single exercise bout was followed by the application of the recovery strategy and multiple post-measurements were taken to give an overview of the effectiveness over time. Other studies, such as those evaluating supplements and compression garments (CG), typically had only one post-measurement. A minority of studies also included several post-measurements at different time points after the initial intervention.

Outcomes for endurance athletes were assessed by the ten most common outcome variables via time to exhaustion (TTE) on a treadmill or cycle ergometer, countermovement jumps (CMJ), and running economy (RE) for performance benefits. To measure physiological adaptations, lactate (La), oxygen consumption (VO_2_), maximum oxygen consumption (VO_2_max), heart rate (HR), and creatine kinase (CK) were evaluated. Furthermore, the rate of perceived exertion (RPE) and muscle soreness were the most used measures for perceived outcome variables.

### Effectiveness of Passive Recovery Strategies

#### General Information on Reviews on Passive Recovery

Nineteen reviews focused on passive recovery, of which eight examined the effects of nutrition or nutrition supplements [25–27, 33, 35, 37, 44, 45], six reviews conducted research on cryotherapy [28, 30, 36, 38, 40, 42] and three reviews focused on the use of CG [24, 29, 39]. Two reviews focused on the effects of massage [31, 43] (see Table 1).

##### Supplements

Used supplements were cow milk [25], chocolate milk [26], milk [35], carbohydrates and protein [33, 37], antioxidants [44, 46], pomegranate [27], and curcumin [45]. Considering all athletes, mixed results were found for the effectiveness of nutrition supplements. Carbohydrate [33, 37] and curcumin supplements [45] benefited recovery, while pomegranate [27] showed a potentially beneficial effect on endurance and strength performance.

##### Temperature-Based

Cryotherapy included whole-body cryotherapy (WBC), partial-body cooling, cold water immersion (CWI), alternating hot and cold-water immersion treatments, and cooling packs. The effectiveness of cryotherapy was mixed across studies with a lack of conclusive evidence. Only one review showed that cryotherapy had negligible positive effects (2.4%, g = 0.28) on recovery performance [42]. Conversely, another review showed that the use of cryotherapy resulted in harmful effects [36].

##### CG

CG included the application of stockings, knee socks/calf sleeves, arm sleeves, whole-body garments, graduated tights, and sleeved tops. In general, marginal to large effects were found for the application of CG indicating a positive effect on recovery.

##### Massage

Massage included automated massage, vibration, warm under-water water jet, pneumatic classic, manual massage, and petrissage, friction, and tapotement. The effect of massage [31, 43] on recovery was marginal or nonexistent (see Table 1).

#### Effects in Endurance Athletes

In total, 1052 endurance athletes participated in 60 studies on passive recovery, and a detailed overview of the distribution can be found in Fig. 2. Not all of the included studies on endurance athletes reported one of the pre-determined (most common) outcome variables (see Additional file 4: Table S4 for an overview).

##### CG

For CG, positive effects on performance variables (TTE and CMJ) were found only twice, while non-significant results in these variables were apparent 12 times across studies. The study by Rider et al. (2014) even found a negative impact of CG on TTE in a maximal treadmill test [47]. Conversely, this study showed a beneficial effect of using CG on lactate concentration one minute after exercise. The only other study that showed a positive effect of using CG on the physiological variables was Driller and Halson (2013) for HR [48], while there were 39 non-significant effects for the other physiological variables (La, VO_2_, VO_2_max, HR, and CK). For self-perceived variables like muscle soreness, two positive [49, 50] and four non-significant results were found across the studies. For RPE, only Rugg and Sternlicht (2013) [51] found a beneficial effect while 13 non-significant and one [52] controversial finding was found.

##### Temperature-Based

For temperature-based recovery strategies, there was no positive or negative effect on performance, while non-significant results were found twice for TTE. Three studies [53–55] showed a positive effect of temperature on HR and CK, while six non-significant effects for the other physiological variables (La, HR, and CK). For self-perceived variables, one positive outcome [56] was observed on muscle soreness and ten non-significant results across the studies (RPE and muscle soreness).

##### Supplements

None of the studies using nutrition supplements for recovery reported any performance variables. The study by Abbiss et al. (2008) found a negative impact on lactate concentration following the carbohydrate compared with the placebo [57]. Other than that, thirteen non-significant effects were observed for the other physiological variables (La, HR, and CK) using supplements. In addition, only one study found a positive effect on RPE while five were non-significant [58]. Two studies found a non-significant effect of supplement use on muscle soreness.

##### Massage

Similarly to nutrition supplements, none of the studies applying massage for recovery reported any performance variable or information on physiological variables like HR and VO2max. RPE was not included in any of these studies either. However, Edge et al. (2009) found non-significant results for whole-body vibration on La, VO_2_, and CK [59]. For self-perceived variables, only two [60, 61] studies showed a positive effect of massage on muscle soreness, while the other two found a non-significant effect on the same variable.

### Effectiveness of Active Recovery Strategies

#### General Information on Reviews on Active Recovery

Two reviews investigated active recovery strategies, once of which examined the effects of foam rolling (FR) [32]. The other review evaluated active recovery (AR) [41] with a variety of submaximal activities, such as running, jogging, cycling, swimming, or active stretching (see Table 1). As in this review studies did not compare the effects of active recovery against a control group (ten studies), we did not include their results in our analysis.

Submaximal activities (e.g., warm and cool down) generally resulted in positive effects on recovery [41]. It was further mentioned that FR should be combined with dynamic stretching and an active warm-up before training at the same time. In general, a reduction of muscle stiffness and DOMS was apparent after FR [32] (see Table 1).

#### Effect in Endurance Athletes

In total, 48 endurance athletes participated in three studies on active recovery (see Fig. 2). All three studies included in the active recovery category reported at least one outcome measure. None of the studies reported any performance and self-perceived variables. Positive effects on La were found twice [62, 63], while there were three non-significant effects for other physiological variables (VO_2_ and HR).

### Effectiveness of Proactive Recovery Strategies

#### General Information on Reviews on Proactive Recovery

Only one proactive recovery review was identified, focusing on alcohol consumption (see Table 1) [34]. For this review, outcomes like force, power, muscular endurance, soreness, and RPE were unaffected during recovery, meaning no negative effects could be found.

#### Effect in Endurance Athletes

No endurance athletes were included in the review of the proactive recovery strategy.

### Training Recovery Time Frame

Five [29, 30, 38, 42, 43] out of twenty-two reviews, focusing on massage [43], cryotherapy [30, 38, 42], and CG [29], compared recovery outcomes at different time points. Eight studies [49, 54, 56, 59, 64–67] from these five reviews examined the effects in endurance athletes 8–24 h after the exercise and qualified for further analysis. Of these eight studies, strength, jump, sprint, and endurance performance were reported (see Table 3).

**Table 3:** Effects of recovery at different time points

Performance	SR and MA	Type	References	Participants	Time	ES	95%	CI
Strength performance	Costello et al. [30]	Cryotherapy^a^	Hausswirth et al. [64]	9M well-trained runners	Post 24 h	3.00	− 2.59	8.59
	Moore et al. [38]	Cryotherapy^b^	Dantas et al. [54]	30M recreational street runners	Post 24 h	0.33	− 0.56	1.21
	Poppendieck et al. [43]	Massage^c^	Dawson et al. [65]	12 (8M, 4F) competitive runners	Post 24 h	0.00	− 0.23	0.27
		Massage^d^	Edge et al. [59]	9M competitive runners	Post 24 h	− 0.03	− 0.29	0.24
	Brown et al. [29]	CG^e^	Bieuzen et al. [66]	11M highly trained runners	Post 24 h	0.83	− 0.05	1.70
		CG^f^	Hill et al. [49]	24 (M17, 7F) recreational marathon runners	Post 24 h	0.35	− 0.46	1.16
Jump performance	Brown et al. [29]	CG^e^	Bieuzen et al. [66]	11M highly trained runners	Post 24 h	0.48	− 0.37	1.33
Sprint performance	Poppendieck et al. [42]	Cryotherapy^g^	Stanley et al. [56]	11M trained cyclists	Post 24 h	0.57	− 0.28	1.42
		Cryotherapy^h^	Vaile et al. [67]	12M cyclists	Post 24 h	1.01	0.16	1.86
Endurance performance	Poppendieck et al. [42]	Cryotherapy^g^	Stanley et al. [56]	11M trained cyclists	Post 24 h	0.12	− 0.72	0.96
		Cryotherapy^h^	Vaile et al. [67]	12M cyclists	Post 24 h	0.10	− 0.70	0.90

CG, compression garments; CI, confidence intervals; ES, effect size; F, female; M, male; MA, meta-analysis; SR, systematic review

^a^Cryogenic chamber; ^b^whole-body cryotherapy; ^c^manual massage; ^d^whole-body vibration massage; ^e^compression knee socks/calf sleeves; ^f^graduated compression tights; ^g^cold water immersion; ^h^hydrotherapy included cold water immersion, hot water immersion, contrast water therapy

Four out of the eight studies applied cryotherapy to enhance recovery, with generally positive outcomes (ES 0.33 to 3.00) [54, 56, 64, 67]. The largest effects were reported for the strength performance of runners, who benefited by 10.8% compared to controls allocated to 3 × 3 min in a cryo-chamber at − 110 °C [64]. Sprint performance of cyclists also improved by 2.6% compared to controls after 24 h using a 14-min whole-body CWI at 15 °C and by 7.8% when using a 5-min whole-body CWI at 10.1 °C. However, the effects were marginal for similar applications for endurance performance [56, 67].

The two studies using massage as a recovery strategy found no benefits compared to controls [59, 65].

Using CG in the form of compression stockings (CS) [66] and graduated tights [29] benefited the recovery of endurance athletes for strength and jump performance after 24 h.

## Discussion

This is the first umbrella review that systematically evaluated recovery strategies for endurance athletes. The main findings were: (1) in terms of methodological quality, only three reviews had high scores (two assessing cryotherapy and one assessing supplementation), while the other included reviews were of low to moderate quality; (2) in general, none of the investigated recovery strategies were effective across the ten parameters; however, the utilization of active recovery in endurance athletes resulted in reduction in lactate concentration following exercise when compared to control groups. Muscle soreness was also reduced after applying different passive recovery strategies (CG, massage, and cryotherapy); (3) The subgroup analysis on training recovery (8–24 h after initial exercise) revealed promising tendencies for CG and cryotherapy. However, there is a scarcity of studies focusing exclusively on training recovery in endurance athletes.

### Methodological Quality

The quality of included reviews mostly (19 out of 22) ranged from low to moderate except for one review. This means that the overall quality of research in this field still needs improvement, with specific recurring issues. Among the common methodological items of AMSTAR 2, only a few studies explicitly addressed the following items, namely: “an explanation of the selected study design”, “a list of excluded studies and rationale”, and “reported funding reports for source studies”. Although all studies included in this UR were published after 2009 and after publishing the PRISMA statement, not all actually adhere to the guidelines. While these items might not drastically influence the quality of evidence, they should be reported. Therefore, authors should pay attention in future studies to properly follow the updated PRISMA statement and explain each item separately.

Arguably, item 9, the RoB, is one of the most vital components in AMSTAR 2. It is an essential tool for assessing the quality of a review. It was found that six out of twenty-two included reviews did not use the appropriate tool to assess the RoB, which demonstrates the increasing attention authors in sports science are paying to the RoB. Other than AMSTAR 2, authors may also consider using the Risk Of Bias In Systematic reviews (ROBIS) tool, a more effective instrument for assessing the RoB. While most authors chose suitable RoB tools, not all of them registered their reviews in PROSPERO or other registration websites. PROSPERO and the Cochrane Database of Systematic Reviews (CDSR) are two databases where systematic review protocols can be registered. Such protocols can help reduce the risk of duplication of reviews by independent research groups. However, even when a review was submitted to PROSPERO, it could still run the risk of not being explicitly registered under the sports discipline. Many systematic reviewers in sports science overlook this critical step. Therefore, as the number of sport-related systematic reviews increases, it will be necessary to recommend a register specifically for this area in the future.

### General Effectiveness of Recovery Strategies in Endurance Athletes

Passive recovery was the most investigated type of recovery with a variety of strategies. Among the 63 studies that included endurance athletes, CG (28 out of 63) was the most extensively researched recovery strategy. Our results indicated that there was no conclusive evidence that CG benefited sports-related variables (RE, VO_2_max, and CK), except for a limited number of studies showing positive results for performance (TTE and CMJ) [51, 68] and physiological (La and HR) outcomes [47, 48]. However, these findings were not consistent across all studies. The results on CG were rather confusing, as highlighted by one study that found cross-country runners had lower La concentration after wearing CS, but a longer time to fatigue in the non-CS group [47]. Our findings also align with the results of the review by Marqués-Jiménez et al. (2016), indicating that CG did not help decrease lactate or creatine kinase levels [69]. Based on previous research, we had anticipated that CG would have an effect on performance-related variables, such as the power and strength [12]. However, this was not the case. One reason for this could be bias in the sample as most studies using CG were conducted with marathon runners wearing CS while running [70–73]. In addition, marathon runners are different from general endurance athletes in that they have different physical and muscular demands [74], which may also affect the outcome indicators of wearing CS.

In addition, contradictory results were found for CMJ [52], VO_2_ [75], and RPE [52] in endurance athletes who utilized CG. A study that investigated the performance of wearing different graduated CS during 10 km time trials found that wearing low (12–15 mm Hg) and medium (18–21 mm Hg) graduated CS improved leg muscle function and CMJ [52]. The effect was even more prominent after intense endurance exercise (five 10-km time trials with 7 days recovery between each trial) compared to control and high (23–32 mm Hg) graduated CS groups [52]. Another study compared the difference between graduated compression tights during a 15-min incremental run at 50, 70, and 85% heart rate reserve [51]. The tights had ankle, calf, and thigh pressure of 18.0 mm Hg, 12.6 mm Hg, and 7.2 mm Hg, respectively, and the results showed a significant improvement in both CMJ and RPE [51]. Comparing the socks from the previous study [52], it was concluded that the highest pressure sock was not necessarily the optimal option for different exercise intensities. These findings also indicated that wearing different types/pressures of socks may have caused the contradictory results and further research is needed to determine the optimal application of CG. It must also be noted that while the included reviews on CG yielded overall benefits for recovery, endurance athletes did not seem to experience a benefit in RPE from wearing CS, especially when compared to soccer players [39].

For temperature-based recovery strategies, we could only draw conclusions for CWI, given the lack of studies on other strategies. We observed that CWI reduced the HR of endurance athletes after a 100 m swim [53] and a 40-min cycle [76] as well as CK levels [54] after a 10 km run using water of 14 °C for 5 min, 11.5 °C for 60 s repeated three times, and 10 °C for 10 min, respectively. A temperature below 14 °C seemed to be essential for a physiological effect of CWI, as a previous study compared the physiological responses of water immersion at three different temperatures (32 °C, 20 °C, and 14 °C) and showed that HR and blood pressure increased in 14 °C compared to warm water [77]. The authors explained that this was due to shivering, which was treated as light exercise, rather than due to the cold. Despite the numerous studies on CWI, the variety in the design of the application, including immersion times, patterns, ambient temperatures, immersion areas, and other factors, make it hard to give concrete recommendations for best practice. It should also be mentioned that a recent review by Malta et al. (2021) found CWI to be harmful in different performance variables [36]. However, these findings are based on non-endurance athletes.

The included supplements-based strategies were ingesting carbohydrates (with and without water) or curcumin [78]. Our UR found that the consumption of carbohydrates and curcumin did not result in positive outcomes for HR and RPE in exercise lasting longer than 90 min during recovery, which was consistent with the study by Saunders et al. (2004) [79]. While these findings are discouraging, it must be noted that the included studies did not examine variables related to performance. Additionally, there was a lack of studies on exercise duration for less than 90 min. In contrast, the review by McCartney et al. (2018) [37] found a beneficial effect of CHO (and CHO + water) for aerobic and endurance exercises (lasting from 45 min to 2 h). However, their analysis also included non-endurance athletes and several intervention groups instead of a comparison against a control group.

A review by Cheung showed that massage did have an effect on DOMS, depending on the type of massage, time, and technique [80]. Also, in our results, massage only affected muscle soreness while other non-self-reported measures (La, VO_2_, and CK) were not affected.

In terms of active recovery strategies, it was shown that active recovery had a positive effect on lactate (concentration) compared to seated rest in swimmers and climbers [62, 63]. Similar results were also found by Mota et al. (2017) in a 200 m freestyle swimming trial, where blood lactate was decreased [81]. However, these results were limited by the intensity of exercise over a very short period of time. In general, the review by Ortiz et al. (2019) recommends an active recovery period of 6 to 10 min after exercise, but they also state that the benefits might be psychological rather than physiological [41].

According to Kellman’s definition, proactive recovery was primarily a form of recovery from social activity as well as self-selection [13]. In terms of proactive recovery strategies, sleep was the most popular one. Unfortunately, no studies related to sleep were included based on the search criteria of this UR, as there was a lack of endurance athletes in these studies. However, several studies on sleep in team sports have been conducted [82, 83], and from these studies, it was observed that sleep was positively associated with recovery and later athletic performance. The only proactive recovery strategy included in this UR was alcohol, but the participants were non-endurance athletes, so further analysis for endurance athletes was not conducted [34]. A previous study of cyclists showed that acute small ethanol (EtOH) consumption during recovery (0.5 ml EtOH/kg fat-free mass, combined with carbohydrate) did not affect recovery but decreased endurance performance [84]. Another review not included in our UR also supported this conclusion [85].

### Effects of Recovery Strategies on Training Recovery

Out of the total of twenty-two reviews, only five investigated recovery strategies (cryotherapy, massage, and CG) with a focus on their effects on training recovery. Within these five reviews, eight studies were eligible for further analysis. We observed that applying passive recovery (CG and cryotherapy) strategies could benefit endurance athletes’ recovery following training sessions.

Applying CG in the form of stockings consistently yielded benefits for runners (see Table 3). However, since the study focused on runners, these benefits might not be transferable to other endurance sports. Furthermore, the effects were only demonstrated for strength and jump performance after 24 h and not for sprint and endurance performance. While no study tested the influence of CG on endurance performance, Brown et al. (2017) suggested that based on data from other sports, beneficial effects of CG are likely [29]. They demonstrated positive effects at 24 h following metabolic exercise or prior to endurance performance, indicating a potential benefit for endurance athletes.

For cryotherapy, mixed results were observed as three studies showed large effects (ES 0.57 to 1.01) and four showed low effects (0.1 to 0.17) (see Table 3). The findings suggest that cryotherapy may have positive effects on sprint performance, but the results were mixed for strength, and marginal effects were observed for jump and endurance performance. The inconsistent results for strength performance could be due to differences in the applied methods. For instance, Hausswirth et al. (2011) reported benefits from WBC (− 110 °C for 3 min) [64], while Vaile et al. (2008) found marginal effects using CWI (15 °C for 14 min) [67]. This indicates that WBC could be more useful. However, similar cold-water immersion protocols did show beneficial effects on sprint performance. Furthermore, one should be cautious when applying whole-body cryotherapy on a regular basis, as excessive exposure to very low temperatures (e.g., ice immersion) can be harmful to training adaptations according to Fröhlich et al. (2014) [86].

Our results suggest that massage does not affect training recovery, except for one study by Viitasalo et al. (1995), who showed an improvement in jump performance after 12 h of underwater jet massage [87]. However, this effect was negative after 20 h, which makes the initial findings questionable. In general, massage appears to have more of a psychological placebo effect with short-term benefits.

### Strengths and Methodological Limitations

The findings of this umbrella review contribute to the current literature. Firstly, this UR is the first comprehensive investigation of different recovery strategies in endurance athletes. Secondly, the results are from not only the reviews but also trace back to individual studies. Thirdly, we present an overview of the effectiveness of various recovery strategies rather than focusing on a single approach. By only including studies that use a control group, we can also provide a better understanding of the benefits of different recovery strategies in endurance athletes.

However, our findings suggest the need for further research on endurance athletes recovering from training to confirm the conclusions drawn from the data collected in this UR. Our research has certain limitations, including a limited number of analyses for the time course of the different recovery strategies. With a rather low number of training recovery studies, it is hard to draw general conclusions. Therefore, our results regarding training recovery need to be interpreted with caution. In addition, given the number of different outcome measures for recovery, it is challenging to compare studies with one another and draw a conclusion from the meta-analysis. Furthermore, the results need to be interpreted with caution as there could be inconsistencies in exercise modality, volume, and recovery protocols, as these were not always reported in the individual studies. In general, the current dearth of high-quality meta-analyses limits our understanding of the application of various recovery strategies at various points during recovery in endurance athletes.

### Suggestions for Future Research

Our analysis highlights that researchers prefer to conduct research on passive strategies in endurance athletes. High-quality studies for active recovery and proactive strategies are lacking. Future studies should clearly follow the relevant statement and specifically register under the sports field catalogue. While there is a body of research on recovery, active recovery strategies are seldom evaluated against control or sham groups. Also, the specific changes in recovery measures over time are relatively unknown. To better understand the processes and mechanisms of recovery, we suggest conducting studies focusing on the time course of recovery. Furthermore, one can suggest that using only one recovery strategy no longer meets the sport’s increasing recovery demands. Future studies might consider utilizing various recovery strategy combinations to allow each athlete to realize recovery to its maximum potential. For instance, combining CWI and massage is better than only using massage to reduce blood lactate levels after submaximal exercise [88]. Finally, when discussing recovery strategies, it is best to focus on one peer group instead of mixing them. For example, comparing endurance athletes with team sport athletes may show different responses to the same treatment.

## Conclusion

In summary, we found that most studies investigating the recovery of endurance athletes utilized nutrition supplements or compression garments. However, based on the studied recovery-related outcome variables, no particular recovery strategy demonstrated consistent benefits across different studies for endurance athletes in general. This means one cannot advise the use of one strategy based on our findings. On the other hand, for a small number of studies, both compression garments and cold-water immersion seem to be promising strategies to enhance performance in terms of training recovery. Nevertheless, further research is needed to confirm these findings.

### Supplementary Information

**Additional file 1.** PRISMA.

**Additional file 2.** Research Terms.

**Additional file 3.** AMSTAR 2 Items.

**Additional file 4.** Included Studies.

**Additional file 5.** Excluded Reviews.

## Data Availability

Detailed information regarding data availability can be found in Additional file 4: Table S4. This table provides comprehensive details on the datasets used, including their sources. All data/information regarding the re-analysis can be found in the supplementary tables.

## Abbreviations

UR: Umbrella review
SR: Systematic reviews
MA: Meta-analyses
SRMA: Systematic reviews with meta-analyses
AMSTAR 2: A Measurement Tool to Assess systematic Reviews 2
PICOT: Population, intervention, comparison, outcome, and time
RoB: Risk of bias
CG: Compression garments
TTE: Time to exhaustion
CMJ: Countermovement jumps
RE: Running economy
La: Lactate
VO_2_: Oxygen consumption
VO_2_max: Maximum oxygen consumption
HR: Heart rate
CK: Creatine kinase
RPE: Rate of perceived exertion
WBC: Whole-body cryotherapy
CWI: Cold water immersion
FR: Foam rolling
CS: Compression stockings
