# Supplementary material for: Effectiveness of Recovery Strategies After Training and Competition in Endurance Athletes: An Umbrella Review
Source: Sports Med Open. 2024 May 16;10:55. doi: 10.1186/s40798-024-00724-6 (PMC11098991; doi:10.1186/s40798-024-00724-6)
Supplement: Supplementary file 2 — Additional file 2. Research Terms. [file 40798_2024_724_MOESM2_ESM.docx]

**Supplementary** **Table S2** Research terms in different databases (Search conducted on December 8, 2022)

| Database | Search syntax |
| --- | --- |
| Cochrane Library | (athle*) AND (recover*) |
| PubMed | (("recovery of function"[MeSH Terms] OR ("recovery"[All Fields] AND "function"[All Fields]) OR "recovery of function"[All Fields] OR "recover*"[All Fields]) AND ("endurance training"[MeSH Terms] OR ("endurance"[All Fields] AND "training"[All Fields]) OR "endurance training"[All Fields] OR "enduran*"[All Fields])) AND (meta-analysis[Filter] OR review[Filter] OR systematicreview[Filter]) |
| Web of Science (including Science Citation Index Expanded, Social Sciences Citation Index, and Arts & Humanities Citation Index) | (Recovery of Function OR recover*) AND (Endurance Training OR enduran*) and Review |
