# Supplementary material for: Effectiveness of Recovery Strategies After Training and Competition in Endurance Athletes: An Umbrella Review
Source: Sports Med Open. 2024 May 16;10:55. doi: 10.1186/s40798-024-00724-6 (PMC11098991; doi:10.1186/s40798-024-00724-6)
Supplement: Supplementary file 4 — Additional file 4. Included Studies. [file 40798_2024_724_MOESM4_ESM.docx]

**Supplementary Table S4** Characteristics of included studies

| Study | N | Participants | Exercise Type | Intervention exercise | Recovery Type | Comparison | TTE | CMJ | RE | La | VO_2_ | VO_2max_ | HR | CK | RPE | Muscle soreness |
| --- | --- | --- | --- | --- | --- | --- | --- | --- | --- | --- | --- | --- | --- | --- | --- | --- |
| Greco et al., 2012[1] | 19M | Trained cyclists | Cycling | (i) incremental test until exhaustion; (ii) 2 to 3 CWRT; (iii) 2 to 3 intermittent submaximal CWRT | Act | 50% power output associated with VO_2_max cycling 　　　　vs. Passive recovery |  |  |  |  | ↔ |  | ↔ |  |  |  |
| Greenwood et al., 2008[2] | 14M | Swimmers | Swiming | 200 yard max effort swim | Act | V_LT_ 5 swimming vs. V_LT_ swimming vs.V_LT_1.5 swimming vs. Control (sitting in a chair or on the pool deck) |  |  |  | *↓ |  |  |  |  |  |  |
| Watts et al., 2000[3] | 15M | Rock climbers | Climbing | 20 m difficult route set on an indoor climbing wall | Act | 25 W recumbent cycling vs. resting condition |  |  |  | *↓ | ↔ |  |  |  |  |  |
| Argus et al., 2013[4] | 11M | Highly trained cyclists | Running | 3 × 30 seconds sprints | CG | CG vs. Electronic muscle stimulation vs. Humidification therapy vs. Control |  |  |  |  |  |  |  |  |  |  |
| Driller and Halson 2013 [5] | 10M | Highly trained cyclists | Cycling | 15 minutes at 70% of peak power output and 15 minutes TT | CG | Full-length lower body CG vs. Above-knee cycling shorts |  |  |  | ↔ |  |  | *↓ |  |  | ↔ |
| Armstrong et al., 2015[6] | 33 (23M, 10F) | Recreational marathon runner | Running | Marathon | CG | Below-knee compression socks vs. Placebo non-compressive below-knee socks | *↑ |  |  |  |  |  |  |  |  |  |
| Hill et al., 2014[7] | 24 (17M, 7F) | Recreational marathon runners | Running | Marathon | CG | Treatment group (lower limb compression tights) vs. Sham treatment group |  |  |  |  |  |  |  | ↔ |  | *↑ |
| Ali et al., 2007[8] | 14M | Recreational runners | Running | Multi-stage fitness test | CG | Knee-length graduated CS vs. Control |  |  |  |  |  |  | ↔ |  | ↔ | *↑ |
| Bieuzen et al., 2014[9] | 11M | Highly trained runners | Running | Simulated trail races-15.6 km with 6.6 km hills | CG | Wearing CS (while running or during recovery) vs. Control (ie, run and recovery without CS) |  | ↔ |  |  |  |  |  | ↔ |  |  |
| Rugg and Sternlicht 2013[10] | 14 (8M, 6F) | Competitive runners | Running | 15 minutes incremental run at 50, 70, 85%  HR reserve | CG | Graduated compression tights vs. non compression (loose fitting running shorts) |  | *↑ |  |  |  |  |  |  | *↑ |  |
| Bovenschen et al., 2013[11] | 13M | Moderately trained runners | Running | 10 km sub maximal running and treadmill steptest until exhaustion | CG | Graduated CS on one leg during running vs. Without Graduated CS on the other leg during running |  |  |  |  |  |  |  |  | ↔ |  |
| Goh et al., 2011[12] | 10M | Recreational runners | Running | 20 minutes at 1st ventilatory threshold followed by run to exhaustion at VO₂max at 10 °C and 32 °C | CG | 10°C with lower body CG vs. 10°C without CG vs. 32°C with CG vs. 32°C without CG | ↔ |  |  |  |  |  |  |  |  |  |
| Stickford et al., 2015[13] | 16M | Highly trained runners | Running | 3 × 4 minutes submaximal treadmill running at 3 constant speeds | CG | Calf-compression sleeves (15–20 mm Hg) vs. Control (without compression sleeves) |  |  | ↔ |  |  |  |  |  |  |  |
| Ali et al., 2010[14] | 10M | Highly trained runners and triathletes | Running | 40 minutes treadmill running at 80 % of VO₂max | CG | Low (12–15 mmHg) vs. High (23–32 mmHg) graduated CS vs. Control (0 mmHg) |  | ↔ |  | ↔ | ↔ |  | ↔ |  | ↔ |  |
| Bringard et al., 2006[15] | 6M | Well-trained runners | Running | Energy cost at 10, 12, 14, 16 km·h-1 and 15 minutes treadmill running at 80 % VO₂max | CG | Compression tights vs. Classic elastic tights vs. Control (conventional shorts) |  |  |  |  |  | ↔ | ↔ |  | ↔ |  |
| Berry and McMurray 1987[16] | 6M | Well-trained runners | Running | Incremental treadmill test until exhaustion | CG | Graduated CS vs. Without wear graduated CS | ↔ |  |  |  |  | ↔ |  |  |  |  |
| Dascombe et al., 2011[17] | 11M | Well-trained runners | Running | Steptest and TTE test at 90 % of VO₂max | CG | Manufacturer-recommended LBCG vs. Undersized LBCG vs. Loose running shorts |  |  | ↔ | ↔ | ↑↓ | ↔ | ↔ |  |  |  |
| Cabri et al., 2010[18] | 6M | Trained runners | Running | Submaximal 5000 m running at a velocity of 85 % of 5000 m personal best | CG |  |  |  |  | ↔ |  |  | ↔ |  | ↔ |  |
| Ali et al., 2011[19] | 12 (9M, 3F) | Competitive runners | Running | 10 km TT | CG | Low (12–15 mm Hg) vs. Medium (18–21 mm Hg) vs. high (23–32 mm Hg) grades of graduated CS vs. Control (0 mm Hg) |  | ↑↓ |  |  |  |  | ↔ |  | ↑↓ |  |
| Sperlich et al., 2010[20] | 15M | Well-trained runners and triathletes | Running | 15 minutes treadmill running at 70 % VO₂max followed by running to exhaustion at VO₂max of previous incremental test | CG | Craft of Scandinavia compression socks vs. Craft compression tights vs. Craft whole-body compression vs. conventional running clothing without compression |  |  |  | ↔ | ↔ | ↔ |  |  | ↔ |  |
| Vercruyssen et al., 2012[21] | 11M | Well-trained runners | Running | 15.6 km trail run | CG | Compression socks vs. Non-compression socks |  | ↔ |  | ↔ |  |  | ↔ |  | ↔ |  |
| Wahl et al., 2012[22] | 9M | Well-trained endurance athletes | Running | 30 minutes sub-maximal running and TTE thereafter using a ramp test | CG | Different levels of sock compression (0, 10, 20, and 40 mmHg) |  |  |  | ↔ | ↔ |  | ↔ |  |  |  |
| Varela-Sanz et al., 2011[23] | 16 (13M, 3F) | Experiment 1: endurance trained athletes | Running | 4 bouts of 6 minutes half-marathon pace treadmill running | CG | Gradual-elastic CS vs. Without wear Gradual-elastic CS |  |  | ↔ |  |  |  |  |  | ↔ |  |
| Varela-Sanz et al., 2011[23] | 12 (10M, 2F) | Experiment 2: endurance trained athletes | Running | Treadmill running until exhaustion at 105% of the athlete's recent 10 km time and 1% grade | CG | Gradual-elastic CS vs. Without wear gradual-elastic CS | ↔ |  | ↔ | ↔ |  | ↔ |  |  | ↔ |  |
| Areces et al., 2015[24] | 34 (30M, 4F) | Experienced runners | Running | marathon | CG | Foot-to-knee graduated CS vs. Control |  | ↔ |  | ↔ |  |  |  | ↔ | ↔ | ↔ |
| Del Coso et al., 2014[25] | 36 NS | Experienced triathletes | Triathlon | Half-ironman triathlon competition | CG | Ankle-to-knee graduated CS vs. Control (regular socks) |  | ↔ |  |  |  |  |  | ↔ | ↔ | ↔ |
| Zadow et al., 2018[26] | 67 (43M, 24F) | Marathon runners | Running | Marathon | CG | Compression sock vs. Control |  |  |  |  |  |  |  |  |  |  |
| Zaleski et al., 2018[27] | 20 (10M, 10F) | Runners | Running | Marathon | CG | Compression sock vs. Control |  |  |  |  |  |  |  | ↔ |  |  |
| Brophy-Williams et al., 2019[28] | 12M | Well-trained runners | Running | Maximal 5 kmTT on treadmill | CG | Wearing compression socks vs. Without compression socks |  |  |  | ↔ | ↔ |  |  |  | ↔ | ↔ |
| Rider et al., 2014[29] | 10 (7M, 3F) | Cross-country runners | Running | Maximal treadmill test | CG | CS vs. Without CS | *↓ |  |  | *↑ | ↔ | ↔ | ↔ |  | ↔ |  |
| Kemmler et al., 2009[30] | 21M | Moderately trained runners | Running | Stepwise speed-incremented treadmill test | CG | Below-knee CS vs. Without below-knee CS |  |  |  | ↔ | ↔ | ↔ | ↔ |  |  |  |
| Parouty et al., 2010[31] | 10 (5M, 5F) | Well-trained swimmers | Swiming | 100 m | Cryo | CWI vs. Control |  |  |  | ↔ |  |  | *↑ |  | ↔ |  |
| Stanley et al., 2013[32] | 11M | Trained cyclists | Cycling | 120 minutes cycling (combined intermittent sprint and time trial) per day, repeated exercise over 3 days | Cryo | CWI vs. Control |  |  |  |  |  |  | ↔ |  | ↔ | *↑ |
| Vaile et al., 2008[33] | 12M | Cyclists | Cycling | 105 minutes cycling (combined intermittent sprint and time trial) per day, repeated exercise over 5 days | Cryo | CWI vs. Hot water immersion vs. CWT vs. Control |  |  |  |  |  |  | ↔ |  | ↔ |  |
| Buchheit et al., 2009[34] | 10M | Cyclists | Cycling | Supramaximal 1 km trial undertaken at 35°C, 40% humidity | Cryo | CWI vs. Control |  |  |  |  |  |  |  |  |  |  |
| Cassar et al., 2010[35] | 8M | Cyclists | Cycling | 30 minutes at 70% of VO₂max | Cryo | CWI vs. CWT vs. Control | ↔ |  |  |  |  |  |  |  |  |  |
| Halson et al., 2008[36] | 11M | Cyclists | Cycling | 40 minutes in an environmental chamber maintained at 34.3°C and 41.2% relative humidity | Cryo | CWI vs. Control |  |  |  | ↔ |  |  | *↑ | ↔ |  | ↔ |
| Halson et al., 2014[37] | 24M | Endurance-trained competitive cyclists | Cycling | Sprint interval training + specific cycling efforts (2 repetitions of 4-min maximal effort following by short repeated sprints (6–20 s), pursuit and TT efforts) | Cryo | CWI vs. Control |  |  |  |  |  |  |  |  |  |  |
| Dantas et al., 2020[38] | 30M | Recreational street runners | Running | 10 km | Cryo | Immersion vs. CWI vs. Control |  |  |  |  |  |  |  | *↑ |  | ↔ |
| Bosak et al., 2009[39] | 12 (9M, 3F) | Well-trained runners | Running | 5 km | Cryo | Ice water immersion vs. Control |  |  |  |  |  |  |  |  | ↔ | ↔ |
| Stenson et al., 2017[40] | 9M | Endurance-trained male | Running | 8 × 1200 m runs at 75% of VO₂peak | Cryo | CWI vs. Control | ↔ |  |  |  |  |  |  |  | ↔ | ↔ |
| Hausswirth et al., 2011[41] | 9M | Well-trained runners | Running | Simulated trial lasting 48 minutes | Cryo | Whole body cryotherapy vs. Far infrared Far infrared vs. Control |  |  |  |  |  |  |  | ↔ |  | ↔ |
| Dawson et al., 2011[42] | 28 (6M, 22F) | Novice recreational runners | Running | 10 km road race | Msg | Massage vs. Control |  |  |  |  |  |  |  |  |  | ↔ |
| Hoffman et al., 2016[43] | 72 NS | Runners | Running | 161 km Western States Endurance Run | Msg | Therapeutic massage (effleurage, compressions and tapotement) vs. Pneumatic compression vs. Control (supine rest) |  |  |  |  |  |  |  |  |  | *↑ |
| Dawson et al., 2004[44] | 12 (12M, 4F) | Recreational runners | Running | Half marathon road race | Msg | Massage legs vs. Control legs |  |  |  |  |  |  |  |  |  | ↔ |
| Nunes et al., 2016[45] | 74 (70M, 4F) | Triathlon athletes | Triathlon | Ironman triathlon competition | Msg | Experimental group (massage to the quadriceps) vs. Control |  |  |  |  |  |  |  |  |  | *↑ |
| Edge et al., 2009[46] | 9M | Competitive runners | Running | 3 km TT and 8 × 400 m | Msg | Whole body vibration vs. Control |  |  |  | ↔ | ↔ |  |  | ↔ |  |  |
| Laupheimer et al., 2014[47] | 7M | Well-trained distance runners | Running | London marathon race 2010 | Sup | Resveratrol (600 mg Resveratrol daily for 7 days immediately before the marathon) vs. Placebo |  |  |  |  |  |  |  |  |  | ↔ |
| Lynn et al., 2015[48] | 21 NS | Recreationally trained runners | Running | Sheffield half marathon | Sup | 2 × 200 ml of bilberry juice vs. Energy matched control drink |  |  |  |  |  |  |  | ↔ |  | ↔ |
| Oosthuyse et al., 2015[49] | 8M | Cyclists | Cycling | 120 minutes at 60% of peak power output | Sup | CHO-only vs. CHO-whey hydrolysate vs. CHO-casein hydrolysate vs. Placebo-water |  |  |  |  |  |  |  |  | ↔ |  |
| Abbiss et al., 2008[50] | 10M | Endurance trained cyclists | Cycling | 90 minutes at 62% of VO₂max | Sup | CHO (0.96 g····kg^1^·h^1^) vs. Placebo gels |  |  |  | *↓ |  |  |  |  |  |  |
| Bonetti et al., 2010[51] | 16 NS | Trained cyclists/triathletes | Cycling | 120 minutes at 55-60% of peak power output | Sup | Mizone vs. Powerade vs. Placebo water |  |  |  | ↔ |  |  | ↔ |  |  |  |
| Cole et al., 1993[52] | 10M | Trained cyclists | Cycling | 105 minutes at 70% of VO₂max | Sup | 6 g% glucose-sucrose (CHO-6GS) vs. 8.3 g% high fructose corn syrup (CHO-8HF) vs. 6.3 g% high fructose corn syrup + 2 g% glucose polymer (CHO-8HP) vs.Water placebo |  |  |  |  |  |  |  |  |  |  |
| El-Sayed et al., 1995[53] | 9M | Competitive cyclists | Cycling | 60 minutes at 70% of VO₂max | Sup | 8% glucose solution (CHO) vs. Placebo solution |  |  |  | ↔ |  |  | ↔ |  |  |  |
| Jarvis et al., 1999[54] | 10F | Trained cyclists | Cycling | 50 minutes at 80% of VO₂max | Sup | 7% CHO electrolyte beverage vs. placebo |  |  |  | ↔ |  |  |  |  | *↑ |  |
| Newell et al., 2015[55] | 20M | Trained cyclists/triathletes | Cycling | 120 minutes at 59% of VO₂max | Sup | 0%, 2%, 3.9% or 6.4% CHO solutions vs. Water placebo |  |  |  |  |  |  |  |  |  |  |
| Osterberg et al., 2008[56] | 13M | Trained cyclists/triathletes | Cycling | 120 minutes at 5% below LT | Sup | CHO vs. CHO + protein vs. Placebo |  |  |  |  |  |  | ↔ |  | ↔ |  |
| Smith et al., 2010[57] | 12M | Recreational cyclists/triathletes | Cycling | 120 minutes constant-load ride at 77% of peak O₂ uptake followed by a 20 km TT | Sup | 1.5, 3.0, or 6.0% glucose solution in water with the electrolytes (15, 30, or 60 g/h glucose) vs. Placebo (water with electrolytes) |  |  |  | ↔ |  |  | ↔ |  |  |  |
| Robson-Anseley et al., 2011[58] | 9M | Trained runners | Running | 120 minutes at 60% of VO₂max | Sup | Carbohydrate vs. Isovolumetric placebo drink |  |  |  |  |  |  | ↔ |  | ↔ |  |
| Potter and Fuller 2015[59] | 10M | Climbers | Climbing | Climbed on the Treadwall set to vertical (90° angle) at a self-determined pace | Sup | Chocolate milk vs. Water |  |  |  | ↔ |  |  | ↔ |  | ↔ |  |
| Chilelli et al., 2016[60] | 47M | Cyclists | Cycling | Aerobic cycling around 200 km weekly | Sup | Group 1: Mediterranean diet + 50 mg Phytome® turmeric (10 mg of + curcumin) + 140 mg of Boswellia extract (105 mg of boswellic acid) vs. Control (Mediterranean diet) |  |  |  |  |  |  |  |  |  |  |
| Sciberras et al., 2015[61] | 11M | Aerobic exercise athletes | Cycling | 120 minutes of aerobic exercise at 95% power from LT | Sup | Curcumin supplementation vs. Placebo supplementation vs. Control (No supplementation) |  |  |  |  |  |  | ↔ |  | ↔ |  |
| Mazani et al., 2014[62] | 28M | Endurance athletes | Running | Treadmill runs at 70 % of maximum HR | Sup | Pomegranate juice vs. Tap water |  |  |  |  |  |  |  |  |  |  |

*, significant; ↑ Positive effect; ↑↓ Contradictory effect; ↓ Negative effect; ↔ No effect; Act, Active recovery; CG, Compression garments; CHO, carbohydrate; CK, creatine kinase; Cryo, Cryotherapy; CS, compression stockings; CWI, cold-water immersion; CWT, Contrast water therapy; CWRT, continuous submaximal constant work rate tests; CMJ, countermovement jumps; F, female; HR, heart rate; L, lactate; M, male; Msg, Massage; NS, Not mentioned; RE, running economy; RPE, rate of perceived exertion; Sup, Supplements; TTE, time to exhaustion; TT, time trial; V_LT_, lactate threshold; VO_2_, oxygen consumption; VO_2_max, maximum oxygen consumption.

Reference:

1. Greco CC, Barbosa LF, Caritá RAC, Denadai BS. Is maximal lactate steady state during intermittent cycling different for active compared with passive recovery? Appl Physiol Nutr Metab. 2012;37:1147–52.

2. Greenwood JD, Moses GE, Bernardino FM, Gaesser GA, Weltman A. Intensity of exercise recovery, blood lactate disappearance, and subsequent swimming performance. J Sports Sci. 2008;26:29–34.

3. Watts PB, Daggett M, Gallagher P, Wilkins B. Metabolic Response During Sport Rock Climbing and the Effects of Active Versus Passive Recovery. Int J Sports Med. 2000;21:185–90.

4. Argus CK, Driller MW, Ebert TR, Martin DT, Halson SL. The Effects of 4 Different Recovery Strategies on Repeat Sprint-Cycling Performance. Int J Sports Physiol Perform. 2013;8:542–8.

5. Driller M, Halson S. The Effects of Wearing Lower Body Compression Garments During a Cycling Performance Test. Int J Sports Physiol Perform. 2013;8:300–6.

6. Armstrong SA, Till ES, Maloney SR, Harris GA. Compression Socks and Functional Recovery Following Marathon Running: a Randomized Controlled Trial. 2015;

7. Hill JA, Howatson G, van Someren KA, Walshe I, Pedlar CR. Influence of Compression Garments on Recovery After Marathon Running. J Strength Cond Res. 2014;28:2228–35.

8. Ali A, Caine MP, Snow BG. Graduated compression stockings: Physiological and perceptual responses during and after exercise. J Sports Sci. 2007;25:413–9.

9. Bieuzen F, Brisswalter J, Easthope C, Vercruyssen F, Bernard T, Hausswirth C. Effect of Wearing Compression Stockings on Recovery After Mild Exercise-Induced Muscle Damage. Int J Sports Physiol Perform. 2014;9:256–64.

10. Rugg S, Sternlicht E. The Effect of Graduated Compression Tights, Compared With Running Shorts, on Counter Movement Jump Performance Before and After Submaximal Running. J Strength Cond Res. 2013;27:1067–73.

11. Bovenschen HJ, Booij MT, Van Der Vleuten CJM. Graduated Compression Stockings for Runners: Friend, Foe, or Fake? J Athl Train. 2013;48:226–32.

12. Goh SS, Laursen PB, Dascombe B, Nosaka K. Effect of lower body compression garments on submaximal and maximal running performance in cold (10°C) and hot (32°C) environments. Eur J Appl Physiol. 2011;111:819–26.

13. Stickford ASL, Chapman RF, Johnston JD, Stager JM. Lower-Leg Compression, Running Mechanics, and Economy in Trained Distance Runners. Int J Sports Physiol Perform. 2015;10:76–83.

14. Ali A, Creasy RH, Edge JA. Physiological effects of wearing graduated compression stockings during running. Eur J Appl Physiol. 2010;109:1017–25.

15. Bringard A, Perrey S, Belluye N. Aerobic Energy Cost and Sensation Responses During Submaximal Running Exercise - Positive Effects of Wearing Compression Tights. Int J Sports Med. 2006;27:373–8.

16. Berry MJ, McMurray RG. Effects of Graduated Compression Stockings on Blood Lactate Following an Exhaustive Bout of Exercise. Am J Phys Med Rehabil [Internet]. 1987;66. Available from: https://journals.lww.com/ajpmr/Fulltext/1987/06000/EFFECTS_OF_GRADUATED_COMPRESSION_STOCKINGS_ON.2.aspx

17. Dascombe BJ, Hoare TK, Sear JA, Reaburn PR, Scanlan AT. The Effects of Wearing Undersized Lower-Body Compression Garments on Endurance Running Performance. Int J Sports Physiol Perform. 2011;6:160–73.

18. Cabri J, Caldonazzi S, Clijsen R. Auswirkung von Kompressionsstrümpfen auf die Ausdauerleistung während eines submaximalen Laufbandtests. Sportverletz · Sportschaden. 2010;24:179–83.

19. Ali A, Creasy RH, Edge JA. The Effect of Graduated Compression Stockings on Running Performance. J Strength Cond Res. 2011;25:1385–92.

20. Sperlich B, Haegele M, Achtzehn S, Linville J, Holmberg H-C, Mester J. Different types of compression clothing do not increase sub-maximal and maximal endurance performance in well-trained athletes. J Sports Sci. 2010;28:609–14.

21. Vercruyssen F, Easthope C, Bernard T, Hausswirth C, Bieuzen F, Gruet M, et al. The influence of wearing compression stockings on performance indicators and physiological responses following a prolonged trail running exercise. Eur J Sport Sci. 2014;14:144–50.

22. Wahl P, Bloch W, Mester J, Born D-P, Sperlich B. Effects of different levels of compression during sub-maximal and high-intensity exercise on erythrocyte deformability. Eur J Appl Physiol. 2012;112:2163–9.

23. Varela-Sanz A, España J, Carr N, Boullosa DA, Esteve-Lanao J. Effects of Gradual-Elastic Compression Stockings on Running Economy, Kinematics, and Performance in Runners. J Strength Cond Res. 2011;25:2902.

24. Areces F, Salinero JJ, Abian-Vicen J, González-Millán C, Ruiz-Vicente D, Lara B, et al. The Use of Compression Stockings During a Marathon Competition to Reduce Exercise-Induced Muscle Damage: Are They Really Useful? J Orthop Sports Phys Ther. 2015;45:462–70.

25. Del Coso J, Areces F, Salinero JJ, González-Millán C, Abián-Vicén J, Soriano L, et al. Compression stockings do not improve muscular performance during a half-ironman triathlon race. Eur J Appl Physiol. 2014;114:587–95.

26. Zadow EK, Adams MJ, Wu SSX, Kitic CM, Singh I, Kundur A, et al. Compression socks and the effects on coagulation and fibrinolytic activation during marathon running. Eur J Appl Physiol. 2018;118:2171–7.

27. Zaleski AL, Pescatello LS, Ballard KD, Panza GA, Adams W, Hosokawa Y, et al. The Influence of Compression Socks During a Marathon on Exercise-Associated Muscle Damage. J Sport Rehabil. 2019;28:724–8.

28. Brophy-Williams N, Driller MW, Kitic CM, Fell JW, Halson SL. Wearing compression socks during exercise aids subsequent performance. J Sci Med Sport. 2019;22:123–7.

29. Rider BC, Coughlin AM, Hew-Butler TD, Goslin BR. Effect of Compression Stockings on Physiological Responses and Running Performance in Division III Collegiate Cross-Country Runners During a Maximal Treadmill Test. J Strength Cond Res. 2014;28:1732–8.

30. Kemmler W, Stengel S von, Köckritz C, Mayhew J, Wassermann A, Zapf J. Effect of Compression Stockings on Running Performance in Men Runners. J Strength Cond Res. 2009;23:101–5.

31. Parouty J, Al Haddad H, Quod M, Leprêtre PM, Ahmaidi S, Buchheit M. Effect of cold water immersion on 100-m sprint performance in well-trained swimmers. Eur J Appl Physiol. 2010;109:483–90.

32. Stanley J, Peake JM, Buchheit M. Consecutive days of cold water immersion: effects on cycling performance and heart rate variability. Eur J Appl Physiol. 2013;113:371–84.

33. Vaile J, Halson S, Gill N, Dawson B. Effect of Hydrotherapy on Recovery from Fatigue. Int J Sports Med. 2008;29:539–44.

34. Buchheit M, Peiffer JJ, Abbiss CR, Laursen PB. Effect of cold water immersion on postexercise parasympathetic reactivation. Am J Physiol-Heart Circ Physiol. 2009;296:H421–7.

35. Cassar S, Kidgell D, Pearce A. The effect of hydrotherapy recovery on central fatigue: A preliminary examination using transcranial magnetic stimulation. J Sci Med Sport. 2010;12:e53.

36. Halson SL, Quod MJ, Martin DT, Gardner AS, Ebert TR, Laursen PB. Physiological Responses to Cold Water Immersion Following Cycling in the Heat. Int J Sports Physiol Perform. 2008;3:331–46.

37. Halson SL, Bartram J, West N, Stephens J, Argus CK, Driller MW, et al. Does Hydrotherapy Help or Hinder Adaptation to Training in Competitive Cyclists? Med Sci Sports Exerc. 2014;46:1631–9.

38. Dantas G, Barros A, Silva B, Belém L, Ferreira V, Fonseca A, et al. Cold-Water Immersion Does Not Accelerate Performance Recovery After 10-km Street Run: Randomized Controlled Clinical Trial. Res Q Exerc Sport. 2020;91:228–38.

39. Bosak A, Bishop P, Green J, Hawver G. Impact of cold water immersion on 5km racing performance. Sport J. 2009;12.

40. Stenson MC, Stenson MR, Matthews TD, Paolone VJ. 5000 Meter Run Performance is not Enhanced 24 Hrs After an Intense Exercise Bout and Cold Water Immersion. J Sports Sci Med. 2017;

41. Hausswirth C, Louis J, Bieuzen F, Pournot H, Fournier J, Filliard J-R, et al. Effects of Whole-Body Cryotherapy vs. Far-Infrared vs. Passive Modalities on Recovery from Exercise-Induced Muscle Damage in Highly-Trained Runners. Lucia A, editor. PLoS ONE. 2011;6:e27749.

42. Dawson KA, Dawson L, Thomas A, Tiidus PM. Effectiveness of regular proactive massage therapy for novice recreational runners. Phys Ther Sport. 2011;12:182–7.

43. Hoffman MD, Badowski N, Chin J, Stuempfle KJ. A Randomized Controlled Trial of Massage and Pneumatic Compression for Ultramarathon Recovery. J Orthop Sports Phys Ther. 2016;46:320–6.

44. Dawson LG, Dawson KA, Tiidus PM. Evaluating the Influence of Massage on Leg Strength, Swelling, and Pain Following a Half-Marathon. J Sports Sci Med. 2004;

45. Nunes GS, Bender PU, de Menezes FS, Yamashitafuji I, Vargas VZ, Wageck B. Massage therapy decreases pain and perceived fatigue after long-distance Ironman triathlon: a randomised trial. J Physiother. 2016;62:83–7.

46. Edge J, Mündel T, Weir K, Cochrane DJ. The effects of acute whole body vibration as a recovery modality following high-intensity interval training in well-trained, middle-aged runners. Eur J Appl Physiol. 2009;105:421–8.

47. Laupheimer M, Perry M, Benton S, Malliaras P, Maffulli N. Resveratrol exerts no effect on inflammatory response and delayed onset muscle soreness after a marathon in male athletes.: a randomised, double-blind, placebo-controlled pilot feasibility study. Transl Med UniSa. 2014;10:38.

48. Lynn A, Garner S, Nelson N, Simper T, Hall A, Ranchordas M. Effect of bilberry juice on muscle damage and inflammation in runners completing a half marathon. Proc Nutr Soc. 2015;74:E287.

49. Oosthuyse T, Carstens M, Millen A. Whey or Casein Hydrolysate with Carbohydrate for Metabolism and Performance in Cycling. Int J Sports Med. 2015;36:636–46.

50. Abbiss CR, Peiffer JJ, Peake JM, Nosaka K, Suzuki K, Martin DT, et al. Effect of carbohydrate ingestion and ambient temperature on muscle fatigue development in endurance-trained male cyclists. J Appl Physiol. 2008;104:1021–8.

51. Bonetti D, Hopkins W. Effects of Hypotonic and Isotonic Sports Drinks on Endurance Performance and Physiology. 2010;

52. Cole KJ, Grandjean PW, Sobszak RJ, Mitchell JB. Effect of carbohydrate composition on fluid balance, gastric emptying, and exercise performance. Int J Sport Nutr Exerc Metab. 1993;3:408–17.

53. El-Sayed MS, Rattu AJ, Roberts I. Effects of carbohydrate feeding before and during prolonged exercise on subsequent maximal exercise performance capacity. Int J Sport Nutr Exerc Metab. 1995;5:215–24.

54. Jarvis AT, Felix SD, Sims S, Coughlin MA, Headley SA. Carbohydrate supplementation fails to improve the sprint performance of female cyclists. J Exerc Physiol. 1999;

55. Newell ML, Hunter AM, Lawrence C, Tipton KD, Galloway SD. The ingestion of 39 or 64 g· hr− 1 of carbohydrate is equally effective at improving endurance exercise performance in cyclists. Int J Sport Nutr Exerc Metab. 2015;25:285–92.

56. Osterberg KL, Zachwieja JJ, Smith JW. Carbohydrate and carbohydrate+ protein for cycling time-trial performance. J Sports Sci. 2008;26:227–33.

57. Smith JW, Zachwieja JJ, Péronnet F, Passe DH, Massicotte D, Lavoie C, et al. Fuel selection and cycling endurance performance with ingestion of [13C] glucose: evidence for a carbohydrate dose response. J Appl Physiol. 2010;108:1520–9.

58. Robson-Ansley P, Walshe I, Ward D. The effect of carbohydrate ingestion on plasma interleukin-6, hepcidin and iron concentrations following prolonged exercise. Cytokine. 2011;53:196–200.

59. Potter JA, Fuller B. The effectiveness of chocolate milk as a post-climbing recovery aid. J Sports Med Phys Fitness. 2015;55:1438–44.

60. Chilelli N, Ragazzi E, Valentini R, Cosma C, Ferraresso S, Lapolla A, et al. Curcumin and Boswellia serrata Modulate the Glyco-Oxidative Status and Lipo-Oxidation in Master Athletes. Nutrients. 2016;8:745.

61. Sciberras JN, Galloway SD, Fenech A, Grech G, Farrugia C, Duca D, et al. The effect of turmeric (Curcumin) supplementation on cytokine and inflammatory marker responses following 2 hours of endurance cycling. J Int Soc Sports Nutr. 2015;12:5.

62. Mazani M, Fard AS, Baghi AN, Nemati A, Mogadam RA. Effect of pomegranate juice supplementation on matrix metalloproteinases 2 and 9 following exhaustive exercise in young healthy males. J Pak Med Assoc. 2014;64:785–90.
