# Supplementary material for: Effectiveness of Recovery Strategies After Training and Competition in Endurance Athletes: An Umbrella Review
Source: Sports Med Open. 2024 May 16;10:55. doi: 10.1186/s40798-024-00724-6 (PMC11098991; doi:10.1186/s40798-024-00724-6)
Supplement: Supplementary file 5 — Additional file 5. Excluded Reviews. [file 40798_2024_724_MOESM5_ESM.docx]

**Supplementary** **Table S5** Characteristics of Excluded Reviews

| **Title** | **Authors** | **Year** | **Exclusion reasons** |
| --- | --- | --- | --- |
| A biomineralization, mechanical and durability features of bacteria-based self-healing concrete-a state of the art review | Kashif Ur Rehman S, Mahmood F, Jameel M, Riaz N, Javed MF, Salmi A, Awad YA. | 2022 | Not about recovery |
| A brief review: factors affecting the length of the rest interval between resistance exercise sets | Willardson JM. | 2006 | Duplicate |
| A brief review: factors affecting the length of the rest interval between resistance exercise sets | Willardson JM. | 2006 | Not about recovery, Not a systematic review or meta-analysis |
| A brief review: how much rest between sets? | Willardson JM. | 2008 | Not about recovery |
| A framework for understanding the training process leading to elite performance | Smith DJ. | 2003 | Duplicate |
| A framework for understanding the training process leading to elite performance | Smith DJ. | 2003 | Not about recovery |
| A literature review of immersion pulmonary edema | Kumar M, Thompson PD. | 2019 | Duplicate |
| A literature review of immersion pulmonary edema | Kumar M, Thompson PD. | 2019 | Focused on disease |
| A mathematical model for training impulse and lactate influx and outflux during exercise | Moxnes JF, Hausken K. | 2009 | Not about recovery |
| A patient in persistent vegetative state and his rehabilitation | Lippert-Gruener M, Angerova Y, Hralova M, Syestkova O. | 2011 | Not about recovery |
| A rationale for training respiratory muscles | Grassino A. | 1984 | Not about recovery |
| A review of the ketogenic diet for endurance athletes: performance enhancer or placebo effect? | Bailey CP, Hennessy E. | 2020 | Duplicate |
| A review of the ketogenic diet for endurance athletes: performance enhancer or placebo effect? | Bailey CP, Hennessy E. | 2020 | Not about recovery |
| A review on strength exercise-induced muscle damage: applications, adaptation mechanisms and limitations | Brentano MA, Martins Kruel LF. | 2011 | Not about recovery |
| A review with special reference to considerations for the optimisation of training protocols to elicit the longest time at or near vo2max | Midgley AW, Mc Naughton LR. | 2006 | Not about recovery |
| A robust framework for low-cost cubesat scientific missions in-orbit recovery, results and lessons learned from UNSW-EC0 | Cheong JW, Southwell BJ, Andrew W, Aboutanios E, Lam C, Croston T, Li L, Green S, Kroh A, Glennon EP, Bultitude J. | 2020 | Not about recovery |
| A soy-based supplement alters energy metabolism but not the exercise-induced stress response | Berg A, Schaffner D, Pohlmann Y, Baumstark MW, Deibert P, König D, Gollhofer A. | 2012 | Not about recovery |
| A systematic comparison of exercise training protocols on animal models of cardiovascular capacity | Feng R, Wang L, Li Z, Yang R, Liang Y, Sun Y, Yu Q, Ghartey-Kwansah G, Sun Y, Wu Y, Zhang W. | 2019 | Focused on physiology |
| A systematic review of muscle morphology and function in intermittent claudication | Harwood AE, King S, Totty J, Smith GE, Vanicek N, Chetter IC. | 2017 | Duplicate |
| A systematic review of muscle morphology and function in intermittent claudication | Harwood AE, King S, Totty J, Smith GE, Vanicek N, Chetter IC. | 2017 | Not about recovery |
| A systematic review of supervised comprehensive functional physiotherapy after radical prostatectomy | Steenstrup B, Cartier M, Nouhaud FX, Kerdelhue G, Gilliaux M. | 2022 | Duplicate |
| A systematic review of supervised comprehensive functional physiotherapy after radical prostatectomy | Steenstrup B, Cartier M, Nouhaud FX, Kerdelhue G, Gilliaux M. | 2022 | Focused on disease, Not about recovery |
| A systematic review of the mysterious caterpillar fungus ophiocordyceps sinensis in dong-chongxiacao ( dōng chóng xià cǎo) and related bioactive ingredients | Lo HC, Hsieh C, Lin FY, Hsu TH. | 2013 | Not about recovery |
| A systematic review on heart-rate recovery to monitor changes in training status in athletes | Daanen HA, Lamberts RP, Kallen VL, Jin A, Van Meeteren NL. | 2012 | Focused on physiology |
| A systematic review on markers of functional overreaching in endurance athletes | Roete AJ, Elferink-Gemser MT, Otter RTA, Stoter IK, Lamberts RP. | 2021 | Duplicate |
| A systematic review on markers of functional overreaching in endurance athletes | Roete AJ, Elferink-Gemser MT, Otter RTA, Stoter IK, Lamberts RP. | 2021 | Not about recovery |
| A systematic review with meta-analysis of mindful exercises on rehabilitative outcomes among poststroke patients | Zou L, Sasaki JE, Zeng N, Wang C, Sun L. | 2018 | Focused on disease |
| A unique case series of novel biomarkers of cardiac damage in cyclists completing the 4800 km race across america (RAAM) | Williams K, George K, Hulton A, Godfrey R, Lahart I, G Wilson M, Charlesworth S, Warburton D, Gaze D, Whyte G. | 2011 | Focused on physiology |
| Accelerating recovery from exercise-induced muscle injuries in triathletes: considerations for olympic distance races | Hotfiel T, Mayer I, Huettel M, Hoppe MW, Engelhardt M, Lutter C, Pöttgen K, Heiss R, Kastner T, Grim C. | 2019 | Duplicate |
| Accelerating recovery from exercise-induced muscle injuries in triathletes: considerations for olympic distance races | Hotfiel T, Mayer I, Huettel M, Hoppe MW, Engelhardt M, Lutter C, Pöttgen K, Heiss R, Kastner T, Grim C. | 2019 | Not a systematic review or meta-analysis |
| Achieving optimal post-exercise muscle protein remodeling in physically active adults through whole food consumption | Vliet SV, Beals JW, Martinez IG, Skinner SK, Burd NA. | 2018 | Duplicate |
| Achieving optimal post-exercise muscle protein remodeling in physically active adults through whole food consumption | Vliet SV, Beals JW, Martinez IG, Skinner SK, Burd NA. | 2018 | Not focused on endurance athletes |
| Acquired and genetic thrombotic risk factors in the athlete | Zadow EK, Adams MJ, Kitic CM, Wu SS, Fell JW. | 2018 | Not about recovery |
| Acupuncture for shoulder pain | Green S, Buchbinder R, Hetrick SE, Cochrane Musculoskeletal Group. | 2005 | Focused on disease |
| Acupuncture for treating acute ankle sprains in adults | Kim TH, Lee MS, Kim KH, Kang JW, Choi TY, Ernst E. | 2014 | Focused on disease |
| Acute and long-term effects of concurrent resistance and swimming training on swimming performance | Arsoniadis G, Botonis P, Bogdanis GC, Terzis G, Toubekis A. | 2022 | Not about recovery |
| Acute cardiovascular responses to interval exercise: a systematic review and meta-analysis | Price KJ, Gordon BA, Bird SR, Benson AC. | 2020 | Focused on physiology |
| Acute changes to biomarkers as a consequence of prolonged strenuous running | Bird SR, Linden M, Hawley JA. | 2014 | Focused on physiology |
| Acute effects of muscle fatigue and recovery on force production and relaxation in endurance, power and strength athletes | Häkkinen K, Myllylä E. | 1990 | Not about recovery |
| Acute effects of warm-up, exercise and recovery-related strategies on assessments of soccer kicking performance: a critical and systematic review | Palucci Vieira LH, Santinelli FB, Carling C, Kellis E, Santiago PRP, Barbieri FA. | 2021 | Duplicate |
| Acute effects of warm-up, exercise and recovery-related strategies on assessments of soccer kicking performance: a critical and systematic review | Palucci Vieira LH, Santinelli FB, Carling C, Kellis E, Santiago PR, Barbieri FA. | 2021 | Focused on team sports |
| Acute ingestion of ketone monoesters and precursors do not enhance endurance exercise performance: a systematic review and meta-analysis | Brooks E, Lamothe G, Nagpal TS, Imbeault P, Adamo K, Kara J, Doucet É. | 2021 | Not about recovery |
| Adaptations of lactate kinetics to mixed and continuous training | Gharbi A, Chamari K, Latiri I, Tabka Z, Zbidi A. | 2008 | Not about recovery |
| Adaptations to post-exercise cold water immersion: friend, foe, or futile? | Ihsan M, Abbiss CR, Allan R. | 2021 | Duplicate |
| Adaptations to post-exercise cold water immersion: friend, foe, or futile? | Ihsan M, Abbiss CR, Allan R. | 2021 | Not a systematic review or meta-analysis |
| Adapted exercise important after stroke. acute and long-term effects of different training programs | Engardt M, Grimby G. | 2005 | Not about recovery |
| Adjunctive therapies in addition to land‐based exercise therapy for osteoarthritis of the hip or knee | French HP, Abbott JH, Galvin R. | 2022 | Not about recovery |
| Aerobic endurance training for cancer patients | Crevenna R, Zielinski C, Keilani MY, Schmidinger M, Bittner C, Nuhr M, Nur H, Marosi C, Fialka-Moser V, Quittan M. | 2003 | Focused on disease |
| Aerobic exercise deconditioning and countermeasures during bed rest | Lee SM, Moore AD, Everett ME, Stenger MB, Platts SH. | 2010 | Duplicate |
| Aerobic exercise deconditioning and countermeasures during bed rest | Lee SM, Moore AD, Everett ME, Stenger MB, Platts SH. | 2010 | Not about recovery |
| Aerobic physical exercise for adult patients with haematological malignancies | Knips L, Bergenthal N, Streckmann F, Monsef I, Elter T, Skoetz N. | 2019 | Focused on disease |
| Aerobic training with blood flow restriction for endurance athletes: potential benefits and considerations of implementation | Smith NDW, Scott BR, Girard O, Peiffer JJ. | 2021 | Not about recovery |
| Age differences in human skeletal muscle fatigue during high-intensity intermittent exercise | Ratel S, Lazaar N, Williams CA, Bedu M, Duché P. | 2003 | Not about recovery |
| Age- and sex-related differences in recovery from high-intensity and endurance exercise: a brief review | Hottenrott L, Ketelhut S, Schneider C, Wiewelhove T, Ferrauti A. | 2021 | Duplicate |
| Age- and sex-related differences in recovery from high-intensity and endurance exercise: a brief review | Hottenrott L, Ketelhut S, Schneider C, Wiewelhove T, Ferrauti A. | 2021 | Not a systematic review or meta-analysis |
| Alcohol withdrawal and conditioning | Little HJ, Stephens DN, Ripley TL, Borlikova G, Duka T, Schubert M, Albrecht D, Becker HC, Lopez MF, Weiss F, Drummond C. | 2005 | Focused on physiology |
| Alcohol, athletic performance and recovery | Vella LD, Cameron-Smith D. | 2010 | Not a systematic review or meta-analysis |
| Altitude training considerations for the winter sport athlete | Chapman RF, Stickford JL, Levine BD. | 2010 | Duplicate |
| Altitude training considerations for the winter sport athlete | Chapman RF, Stickford JL, Levine BD. | 2010 | Not about recovery |
| Altitude training improves glycemic control | Chen SM, Lin HY, Kuo CH. | 2013 | Duplicate |
| Altitude training improves glycemic control | Chen SM, Lin HY, Kuo CH. | 2013 | Focused on physiology |
| American college of sports medicine position stand. nutrition and athletic performance | Rodriguez NR, Di Marco NM, Langley S. | 2009 | Not a systematic review or meta-analysis |
| Amino acid supplementation and exercise performance. analysis of the proposed ergogenic value | Kreider RB, Miriel V, Bertun E. | 1993 | Duplicate |
| Amino-acid supplementation and exercise performance - analysis of the proposed ergogenic value | Kreider RB, Miriel V, Bertun E. | 1993 | Not a systematic review or meta-analysis |
| Ampk pathway gene regulation by physical exercise: systematic review and in silico analysis | Pacheco C, Santos LH, Alves JO, Queiroz AN, Soares PM, Ceccatto VM. | 2017 | Not about recovery |
| An official systematic review of the european respiratory society/american thoracic society: measurement properties of field walking tests in chronic respiratory disease | Singh SJ, Puhan MA, Andrianopoulos V, Hernandes NA, Mitchell KE, Hill CJ, Lee AL, Camillo CA, Troosters T, Spruit MA, Carlin BW. | 2014 | Focused on disease |
| Anabolic steroids for rehabilitation after hip fracture in older people | Farooqi V, van den Berg ME, Cameron ID, Crotty M. | 2014 | Not about recovery |
| Analgesic and anti-inflammatory drugs in sports: implications for exercise performance and training adaptations | Lundberg TR, Howatson G. | 2018 | Duplicate |
| Analgesic and anti-inflammatory drugs in sports: implications for exercise performance and training adaptations | Lundberg TR, Howatson G. | 2018 | Not about recovery |
| Analogies between heart and respiratory muscle failure. importance to clinical practice | Köhler D. | 2009 | Focused on disease |
| Analysis of physical therapy application in children with acute lymphoblastic leukemia (literature review) | Bas OA, Ivasyk NO, Tyravska OI, Hertsyk AM. | 2021 | Focused on disease |
| Analysis of the results of heel-rise test with sensors: a systematic review | Pires IM, Ponciano V, Garcia NM, Zdravevski E. | 2020 | Not about recovery |
| Antidepressant treatment for postnatal depression | Brown JV, Wilson CA, Ayre K, Robertson L, South E, Molyneaux E, Trevillion K, Howard LM, Khalifeh H. | 2021 | Focused on disease |
| Antidepressants for depression in adults with HIV infection | Eshun‐Wilson I, Siegfried N, Akena DH, Stein DJ, Obuku EA, Joska JA. | 2018 | Focused on disease |
| Antidepressants plus benzodiazepines for adults with major depression | Ogawa Y, Takeshima N, Hayasaka Y, Tajika A, Watanabe N, Streiner D, Furukawa TA. | 2019 | Not about recovery |
| Antioxidant supplements and endurance exercise: current evidence and mechanistic insights | Mason SA, Trewin AJ, Parker L, Wadley GD. | 2020 | Not a systematic review or meta-analysis |
| Antioxidants and exercise performance: with a focus on vitamin E and C supplementation | Higgins MR, Izadi A, Kaviani M. | 2020 | Not about recovery |
| Application of protein or protein hydrolysates to improve postexercise recovery | van Loon LJ. | 2007 | Not a systematic review or meta-analysis |
| Applications of fish oil supplementation for special operators | Heileson JL, Funderburk LK, Cardaci TD. | 2021 | Not focused on endurance athletes |
| Applications of omega-3 polyunsaturated fatty acid supplementation for sport performance | Philpott JD, Witard OC, Galloway SDR. | 2019 | Duplicate |
| Applications of omega-3 polyunsaturated fatty acid supplementation for sport performance | Philpott JD, Witard OC, Galloway SDR. | 2019 | Not about recovery |
| Applied sport science of rugby league | Johnston RD, Gabbett TJ, Jenkins DG. | 2014 | Duplicate |
| Applied sport science of rugby league | Johnston RD, Gabbett TJ, Jenkins DG. | 2014 | Not about recovery |
| Applying heart rate variability to monitor health and performance in tactical personnel: a narrative review | Stephenson MD, Thompson AG, Merrigan JJ, Stone JD, Hagen JA. | 2021 | Not a systematic review or meta-analysis |
| Approaches for discontinuation versus continuation of long‐term antidepressant use for depressive and anxiety disorders in adults | Van Leeuwen E, van Driel ML, Horowitz MA, Kendrick T, Donald M, De Sutter AI, Robertson L, Christiaens T. | 2021 | Not about recovery |
| Are children metabolically born endurant? | Ratel S. | 2016 | Not about recovery |
| Are measures of physical function of the neck region associated with poor prognosis following a whiplash trauma? a systematic review | Alalawi A, Mazaheri M, Gallina A, Luque-Suarez A, Sterling M, Falla D. | 2022 | Not about recovery |
| Are measures of physical function of the neck region associated with poor prognosis following a whiplash trauma?: a systematic review | Alalawi A, Mazaheri M, Gallina A, Luque-Suarez A, Sterling M, Falla D. | 2021 | Duplicate |
| Are there benefits from the use of fish oil supplements in athletes? a systematic review | Lewis NA, Daniels D, Calder PC, Castell LM, Pedlar CR. | 2020 | Duplicate |
| Are there benefits from the use of fish oil supplements in athletes? a systematic review | Lewis NA, Daniels D, Calder PC, Castell LM, Pedlar CR. | 2020 | Not eligible |
| Are women less susceptible to exercise-induced muscle damage? | Clarkson PM, Hubal MJ. | 2001 | Not about recovery |
| Assessment and psychologic factors in stroke rehabilitation | Kelly-Hayes M, Paige C. | 1995 | Not about recovery |
| Astaxanthin in exercise metabolism, performance and recovery: a review | Brown DR, Gough LA, Deb SK, Sparks SA, McNaughton LR. | 2018 | Duplicate |
| Astaxanthin in exercise metabolism, performance and recovery: a review | Brown DR, Gough LA, Deb SK, Sparks SA, McNaughton LR. | 2018 | Not a systematic review or meta-analysis |
| Autonomic control of heart rate during and after exercise - measurements and implications for monitoring training status | Borresen J, Lambert MI. | 2008 | Not about recovery |
| Autonomic control of heart rate during and after exercise : measurements and implications for monitoring training status | Borresen J, Lambert MI. | 2008 | Duplicate |
| Balance of carbohydrate and lipid utilization during exercise - the crossover concept | Brooks GA, Mercier J. | 1994 | Focused on physiology |
| Basic recovery aids: what's the evidence? | Peterson AR, Smoot MK, Erickson JL, Mathiasen RE, Kregel KC, Hall M. | 2015 | Not a systematic review or meta-analysis |
| Bcaa metabolism and insulin sensitivity - dysregulated by metabolic status? | Gannon NP, Schnuck JK, Vaughan RA. | 2018 | Duplicate |
| Bcaa metabolism and insulin sensitivity - dysregulated by metabolic status? | Gannon NP, Schnuck JK, Vaughan RA. | 2018 | Not a systematic review or meta-analysis |
| Beat the exercise-induced muscle damage | Qamar MM, Javed MS, Dogar MZ, Basharat A. | 2019 | Not about recovery, Not a systematic review or meta-analysis |
| Beetroot juice - a suitable post-marathon metabolic recovery supplement? | Stander Z, Luies L, van Reenen M, Howatson G, Keane KM, Clifford T, Stevenson EJ, Loots DT. | 2021 | Not a systematic review or meta-analysis |
| Behavioural activation therapy for depression in adults | Uphoff E, Ekers D, Robertson L, Dawson S, Sanger E, South E, Samaan Z, Richards D, Meader N, Churchill R. | 2020 | Not about recovery |
| Behavioural and cognitive behavioural therapy for obsessive compulsive disorder (OCD) in individuals with autism spectrum disorder (ASD) | Elliott SJ, Marshall D, Morley K, Uphoff E, Kumar M, Meader N. | 2021 | Not about recovery |
| Beneficial effects of protein hydrolysates in exercise and sports nutrition | Yuan J, Jiang B, Li K, Shen W, Tang JL. | 2017 | Focused on physiology |
| Beneficial role of blood flow restriction exercise in heart disease and heart failure using the muscle hypothesis of chronic heart failure and a growing literature | Cahalin LP, Formiga MF, Owens J, Anderson B, Hughes L. | 2022 | Duplicate |
| Beneficial role of blood flow restriction exercise in heart disease and heart failure using the muscle hypothesis of chronic heart failure and a growing literature | Cahalin LP, Formiga MF, Owens J, Anderson B, Hughes L. | 2022 | Focused on disease |
| Benefits of creatine supplementation for vegetarians compared to omnivorous athletes: a systematic review | Kaviani M, Shaw K, Chilibeck PD. | 2020 | Duplicate |
| Benefits of creatine supplementation for vegetarians compared to omnivorous athletes: a systematic review | Kaviani M, Shaw K, Chilibeck PD. | 2020 | Not about recovery |
| Benefits of daytime napping opportunity on physical and cognitive performances in physically active participants: a systematic review | Souabni M, Hammouda O, Romdhani M, Trabelsi K, Ammar A, Driss T. | 2021 | Duplicate |
| Benefits of daytime napping opportunity on physical and cognitive performances in physically active participants: a systematic review | Souabni M, Hammouda O, Romdhani M, Trabelsi K, Ammar A, Driss T. | 2021 | Not focused on endurance athletes |
| Benefits of exercise training in chronic heart failure | Tabet JY, Meurin P, Driss AB, Weber H, Renaud N, Grosdemouge A, Beauvais F, Cohen-Solal A. | 2009 | Focused on disease |
| Beta2‐agonists for exercise‐induced asthma | Bonini M, Di Mambro C, Calderon MA, Compalati E, Schünemann H, Durham S, Canonica GW. | 2013 | Focused on disease |
| Beyond muscle hypertrophy: why dietary protein is important for endurance athletes | Moore DR, Camera DM, Areta JL, Hawley JA. | 2014 | Duplicate |
| Beyond muscle hypertrophy: why dietary protein is important for endurance athletes | Moore DR, Camera DM, Areta JL, Hawley JA. | 2014 | Not about recovery |
| Biochemical aspects of overtraining in endurance sports - a review | Petibois C, Cazorla G, Poortmans JR, Déléris G. | 2002 | Not about recovery |
| Biochemical aspects of overtraining in endurance sports: a review | Petibois C, Cazorla G, Poortmans JR, Déléris G. | 2002 | Duplicate |
| Biomarkers in sports and exercise: tracking health, performance, and recovery in athletes | Lee EC, Fragala MS, Kavouras SA, Queen RM, Pryor JL, Casa DJ. | 2017 | Duplicate |
| Biomarkers in sports and exercise: tracking health, performance, and recovery in athletes | Lee EC, Fragala MS, Kavouras SA, Queen RM, Pryor JL, Casa DJ. | 2017 | Focused on physiology |
| Blood hormones as markers of training stress and overtraining | Urhausen A, Gabriel H, Kindermann W. | 1995 | Duplicate |
| Blood hormones as markers of training stress and overtraining | Urhausen A, Gabriel H, Kindermann W. | 1995 | Not about recovery |
| Blood pressure regulation x: what happens when the muscle pump is lost? post-exercise hypotension and syncope | Halliwill JR, Sieck DC, Romero SA, Buck TM, Ely MR. | 2014 | Not about recovery |
| Bodyweight high-intensity interval training: a systematic review | Machado AF, Miranda ML, Rica RL, Figueira Junior A, Bocalini DS. | 2018 | Not about recovery |
| Botulinum toxin a as an adjunct to treatment in the management of the upper limb in children with spastic cerebral palsy (update) | Hoare BJ, Wallen MA, Imms C, Villanueva E, Rawicki HB, Carey L. | 2010 | Focused on disease |
| Branched-chain amino acids activate key enzymes in protein synthesis after physical exercise | Blomstrand E, Eliasson J, Karlsson HK, Köhnke R. | 2006 | Not about recovery |
| Brief exposure to systemic hypoxia enhances plasticity of the central nervous system in spinal cord injured animals and man | Sandhu MS, Rymer WZ. | 2021 | Duplicate |
| Brief exposure to systemic hypoxia enhances plasticity of the central nervous system in spinal cord injured animals and man | Sandhu MS, Rymer WZ. | 2021 | Not about recovery |
| Bringing light into the dark: effects of compression clothing on performance and recovery | Born DP, Sperlich B, Holmberg HC. | 2013 | Duplicate |
| Bringing light into the dark: effects of compression clothing on performance and recovery | Born DP, Sperlich B, Holmberg HC. | 2013 | Low quality |
| Building a beverage for recovery from endurance activity: a review | Spaccarotella KJ, Andzel WD. | 2011 | Duplicate |
| Building a beverage for recovery from endurance activity: a review | Spaccarotella KJ, Andzel WD. | 2011 | Not a systematic review or meta-analysis |
| Building muscle: nutrition to maximize bulk and strength adaptations to resistance exercise training | Tarnopolsky MA. | 2008 | Not about recovery |
| Caffeine and exercise: what next? | Pickering C, Grgic J. | 2019 | Not a systematic review or meta-analysis |
| Caffeine for the prevention of injuries and errors in shift workers | Ker K, Edwards PJ, Felix LM, Blackhall K, Roberts I, Cochrane Injuries Group. | 2010 | Not about recovery |
| Can muscle regeneration fail in chronic inflammation: a weakness in inflammatory myopathies? | Loell I, Lundberg IE. | 2011 | Duplicate |
| Can muscle regeneration fail in chronic inflammation: a weakness in inflammatory myopathies? | Loell I, Lundberg IE. | 2011 | Not about recovery |
| Can proprioception really be improved by exercises? | Ashton-Miller JA, Wojtys EM, Huston LJ, Fry-Welch D. | 2001 | Not about recovery |
| Carb-conscious: the role of carbohydrate intake in recovery from exercise | Gonzalez JT, Wallis GA. | 2021 | Duplicate |
| Carb-conscious: the role of carbohydrate intake in recovery from exercise | Gonzalez JT, Wallis GA. | 2021 | Not a systematic review or meta-analysis |
| Carbohydrate consumption and periodization strategies applied to elite soccer players | Fernandes HS. | 2020 | Not focused on endurance athletes |
| Carbohydrate for athletic training and performance | Costill DL. | 1991 | Not about recovery |
| Carbohydrate ingestion during team games exercise: current knowledge and areas for future investigation | Phillips SM, Sproule J, Turner AP. | 2011 | Not focused on endurance athletes |
| Carbohydrate ingestion/supplementation or resistance exercise and training | Conley MS, Stone MH. | 1996 | Not about recovery |
| Carbohydrate nutrition and team sport performance | Williams C, Rollo I. | 2015 | Not focused on endurance athletes |
| Carbohydrate supplementation and resistance training | Haff GG, Lehmkuhl MJ, McCoy LB, Stone MH. | 2003 | Not about recovery |
| Carbohydrate-protein intake and recovery from endurance exercise: is chocolate milk the answer? | Saunders MJ. | 2011 | Not a systematic review or meta-analysis |
| Carbohydrates and exercise | Hargreaves M. | 1991 | Not a systematic review or meta-analysis |
| Carbohydrates for training and competition | Burke LM, Hawley JA, Wong SH, Jeukendrup AE. | 2011 | Not about recovery |
| Cardiac biomarker release after exercise in healthy children and adolescents: a systematic review and meta-analysis | Cirer-Sastre R, Legaz-Arrese A, Corbi F, George K, Nie J, Carranza-García LE, Reverter-Masià J. | 2019 | Not about recovery |
| Cardiac rehabilitation following myocardial infarction. a practical approach | Todd IC, Wosornu D, Stewart I, Wild T. | 1992 | Duplicate |
| Cardiac rehabilitation following myocardial-infarction - a practical approach | Todd IC, Wosornu D, Stewart I, Wild T. | 1992 | Not about recovery |
| Cardio-selective beta-blocker: pharmacological evidence and their influence on exercise capacity | Ladage D, Schwinger RH, Brixius K. | 2013 | Not about recovery |
| Cardiovascular benefits and potential hazards of physical exercise in elderly people | Kallinen M. | 2005 | Not about recovery |
| Child-adult differences in muscle activation - a review | Dotan R, Mitchell C, Cohen R, Klentrou P, Gabriel D, Falk B. | 2012 | Not about recovery |
| Child-adult differences in muscle activation--a review | Dotan R, Mitchell C, Cohen R, Klentrou P, Gabriel D, Falk B. | 2012 | Duplicate |
| Chocolate milk for recovery from exercise: a systematic review and meta-analysis of controlled clinical trials | Amiri M, Ghiasvand R, Kaviani M, Forbes SC, Salehi-Abargouei A. | 2019 | Duplicate |
| Chocolate milk: a post-exercise recovery beverage for endurance sports | Pritchett K, Pritchett R. | 2012 | Not a systematic review or meta-analysis |
| Chronic cannabis consumption and physical exercise performance in healthy adults: a systematic review | Kramer A, Sinclair J, Sharpe L, Sarris J. | 2020 | Duplicate |
| Chronic cannabis consumption and physical exercise performance in healthy adults: a systematic review | Kramer A, Sinclair J, Sharpe L, Sarris J. | 2020 | Not about recovery |
| Chronic exercise training effects on immune function | Mackinnon LT. | 2000 | Not about recovery |
| Circuit class therapy for improving mobility after stroke | English, C and Hillier, SL and Lynch, EA | 2017 | Focused on disease |
| Client feedback in psychological therapy for children and adolescents with mental health problems | Bergman H, Kornør H, Nikolakopoulou A, Hanssen‐Bauer K, Soares‐Weiser K, Tollefsen TK, Bjørndal A. | 2018 | Not about recovery |
| Clinical applications of electrical stimulation after spinal cord injury | Creasey GH, Ho CH, Triolo RJ, Gater DR, DiMarco AF, Bogie KM, Keith MW. | 2004 | Not about recovery |
| Clinical benefit of fixed-dose dual bronchodilation with glycopyrronium and indacaterol once daily in patients with chronic obstructive pulmonary disease: a systematic review | Ulrik CS. | 2014 | Focused on disease |
| Clinical non-superiority of technology-assisted gait training with body weight support in patients with subacute stroke: a meta-analysis | Hsu CY, Cheng YH, Lai CH, Lin YN. | 2020 | Focused on disease |
| Cognitive behavioural therapy for anxiety disorders in children and adolescents | James AC, Reardon T, Soler A, James G, Creswell C. | 2020 | Not about recovery |
| Coingestion of carbohydrate-protein during endurance exercise: influence on performance and recovery | Saunders MJ. | 2007 | Not a systematic review or meta-analysis |
| Cold-water immersion for athletic recovery: one size does not fit all | Stephens JM, Halson S, Miller J, Slater GJ, Askew CD. | 2017 | Duplicate |
| Cold-water immersion for athletic recovery: one size does not fit all | Stephens JM, Halson S, Miller J, Slater GJ, Askew CD. | 2017 | Not a systematic review or meta-analysis |
| Combined bronchodilators (tiotropium plus olodaterol) for patients with chronic obstructive pulmonary disease | Ramadan WH, Kabbara WK, El Khoury GM, Al Assir SA. | 2015 | Focused on disease |
| Combining cooling or heating applications with exercise training to enhance performance and muscle adaptations | Hyldahl RD, Peake JM. | 2020 | Not about recovery |
| Common problems in endurance athletes | Cosca DD, Navazio F. | 2007 | Not a systematic review or meta-analysis |
| Comparative effectiveness of continuation and maintenance treatments for persistent depressive disorder in adults | Machmutow K, Meister R, Jansen A, Kriston L, Watzke B, Härter MC, Liebherz S. | 2019 | Not about recovery |
| Complications and reinterventions after evar: are they decreasing in incidence? | Donas KP, Torsello G. | 2011 | Not about recovery |
| Compression garments and recovery from exercise: a meta-analysis | Brown F, Gissane C, Howatson G, van Someren K, Pedlar C, Hill J. | 2017 | Duplicate |
| Concurrent training in team sports: a systematic review | Seipp D, Quittmann OJ, Fasold F, Klatt S. | 2022 | Not about recovery, Not focused on endurance athletes |
| Consensus recommendations on training and competing in the heat | Racinais S, Alonso JM, Coutts AJ, Flouris AD, Girard O, González-Alonso J, Hausswirth C, Jay O, Lee JK, Mitchell N, Nassis GP, Nybo L, Pluim BM, Roelands B, Sawka MN, Wingo JE, Périard JD. | 2015 | Duplicate |
| Consensus recommendations on training and competing in the heat | Racinais S, Alonso JM, Coutts AJ, Flouris AD, Girard O, González-Alonso J, Hausswirth C, Jay O, Lee JK, Mitchell N, Nassis GP, Nybo L, Pluim BM, Roelands B, Sawka MN, Wingo J, Périard JD. | 2015 | Duplicate |
| Consensus recommendations on training and competing in the heat | Racinais S, Alonso JM, Coutts AJ, Flouris AD, Girard O, González-Alonso J, Hausswirth C, Jay O, Lee JK, Mitchell N, Nassis GP, Nybo L, Pluim BM, Roelands B, Sawka MN, Wingo J, Périard JD. | 2015 | Duplicate |
| Consensus recommendations on training and competing in the heat | Racinais S, Alonso JM, Coutts AJ, Flouris AD, Girard O, González-Alonso J, Hausswirth C, Jay O, Lee JK, Mitchell N, Nassis GP, Nybo L, Pluim BM, Roelands B, Sawka MN, Wingo JE, Périard JD. | 2015 | Duplicate |
| Consensus recommendations on training and competing in the heat | Racinais S, Alonso JM, Coutts AJ, Flouris AD, Girard O, González-Alonso J, Hausswirth C, Jay O, Lee JK, Mitchell N, Nassis GP, Nybo L, Pluim BM, Roelands B, Sawka MN, Wingo JE, Périard JD. | 2015 | Not about recovery |
| Contemporary periodization of altitude training for elite endurance athletes: a narrative review | Mujika I, Sharma AP, Stellingwerff T. | 2019 | Duplicate |
| Contemporary periodization of altitude training for elite endurance athletes: a narrative review | Mujika I, Sharma AP, Stellingwerff T. | 2019 | Not about recovery, Not a systematic review or meta-analysis |
| Continuation and maintenance treatments for depression in older people | Wilkinson P, Izmeth Z. | 2016 | Focused on disease |
| Cooling and performance recovery of trained athletes: a meta-analytical review | Poppendieck W, Faude O, Wegmann M, Meyer T. | 2013 | Duplicate |
| Cooling interventions for the protection and recovery of exercise performance from exercise-induced heat stress | Duffield R. | 2008 | Not a systematic review or meta-analysis |
| Couple and family therapies for post‐traumatic stress disorder (PTSD) | Suomi A, Evans L, Rodgers B, Taplin S, Cowlishaw S. | 2019 | Not about recovery |
| Covid-19: a new challenge for pulmonary rehabilitation? | Kołodziej M, Wyszyńska J, Bal-Bocheńska M. | 2021 | Duplicate |
| Covid-19: a new challenge for pulmonary rehabilitation? | Kołodziej M, Wyszyńska J, Bal-Bocheńska M. | 2021 | Focused on disease |
| Cow's milk as a post-exercise recovery drink: implications for performance and health | James LJ, Stevenson EJ, Rumbold PL, Hulston CJ. | 2019 | Not a systematic review or meta-analysis |
| Crawling to the finish line: why do endurance runners collapse? implications for understanding of mechanisms underlying pacing and fatigue | St Clair Gibson A, De Koning JJ, Thompson KG, Roberts WO, Micklewright D, Raglin J, Foster C. | 2013 | Duplicate |
| Crawling to the finish line: why do endurance runners collapse? implications for understanding of mechanisms underlying pacing and fatigue | St Clair Gibson A, De Koning JJ, Thompson KG, Roberts WO, Micklewright D, Raglin J, Foster C. | 2013 | Focused on physiology |
| Creatine for exercise and sports performance, with recovery considerations for healthy populations | Wax B, Kerksick CM, Jagim AR, Mayo JJ, Lyons BC, Kreider RB. | 2021 | Duplicate |
| Creatine for exercise and sports performance, with recovery considerations for healthy populations | Wax B, Kerksick CM, Jagim AR, Mayo JJ, Lyons BC, Kreider RB. | 2021 | Not focused on endurance athletes |
| Creatine for treating muscle disorders | Kley RA, Tarnopolsky MA, Vorgerd M. | 2013 | Not about recovery |
| Creatine supplementation and exercise performance: an update | Williams MH, Branch JD. | 1998 | Not a systematic review or meta-analysis |
| Creatine supplementation as an ergogenic aid for sports performance in highly trained athletes: a critical review | Mujika I, Padilla S. | 1997 | Not a systematic review or meta-analysis |
| Crossfit (r) training strategies from the perspective of concurrent training: a systematic review | Mujika I, Padilla S. | 2020 | Not about recovery |
| Current perspectives on anabolic androgenic steroid abuse | Lukas SE. | 1993 | Not about recovery |
| Current perspectives on anabolic-androgenic steroid abuse | Lukas SE. | 1993 | Duplicate |
| Cystine and theanine: amino acids as oral immunomodulative nutrients | Kurihara S, Shibakusa T, Tanaka KA. | 2013 | Not about recovery |
| Decrease in respiratory quotient during exercise following l-carnitine supplementation | Gorostiaga EM, Maurer CA, Eclache JP. | 1989 | Focused on physiology |
| Determinants for success in climbing: a systematic review | Saul D, Steinmetz G, Lehmann W, Schilling AF. | 2019 | Duplicate |
| Determinants for success in climbing: a systematic review | Saul D, Steinmetz G, Lehmann W, Schilling AF. | 2019 | Not about recovery |
| Determinants of post-exercise glycogen synthesis during short-term recovery | Jentjens R, Jeukendrup A. | 2003 | Focused on physiology |
| Development of a revised conceptual framework of physical training for use in research and practice | Jeffries AC, Marcora SM, Coutts AJ, Wallace L, McCall A, Impellizzeri FM. | 2022 | Not about recovery |
| Diastolic function in healthy humans: non-invasive assessment and the impact of acute and chronic exercise | George KP, Naylor LH, Whyte GP, Shave RE, Oxborough D, Green DJ. | 2010 | Not about recovery |
| Diet and physical performance | Montain SJ, Young AJ. | 2002 | Not about recovery |
| Diet, physical activity and behavioural interventions for the treatment of overweight or obese children from the age of 6 to 11 years | Mead E, Brown T, Rees K, Azevedo LB, Whittaker V, Jones D, Olajide J, Mainardi GM, Corpeleijn E, O'Malley C, Beardsmore E. | 2017 | Not about recovery |
| Dietary fuels in athletic performance | Fritzen AM, Lundsgaard AM, Kiens B. | 2019 | Duplicate |
| Dietary fuels in athletic performance | Fritzen AM, Lundsgaard AM, Kiens B. | 2019 | Not about recovery |
| Dietary sugars, exercise and hepatic carbohydrate metabolism | Gonzalez JT, Betts JA. | 2019 | Not a systematic review or meta-analysis |
| Dietary thiols in exercise: oxidative stress defence, exercise performance, and adaptation | McLeay Y, Stannard S, Houltham S, Starck C. | 2017 | Duplicate |
| Dietary thiols in exercise: oxidative stress defence, exercise performance, and adaptation | McLeay Y, Stannard S, Houltham S, Starck C. | 2017 | Not about recovery |
| Dietary-supplements in sport | Burke LM, Read RS. | 1993 | Not a systematic review or meta-analysis |
| Differences in time to task failure and fatigability between children and young adults: a systematic review and meta-analysis | Souron R, Carayol M, Martin V, Piponnier E, Duché P, Gruet M. | 2022 | Not focused on endurance athletes |
| Disentangling the consequences of growth temperature and adult acclimation temperature on starvation and thermal tolerance in the red flour beetle | Scharf I, Galkin N, Halle S. | 2015 | Not about recovery |
| Do antioxidant supplements interfere with skeletal muscle adaptation to exercise training? | Merry TL, Ristow M. | 2016 | Duplicate |
| Do antioxidant supplements interfere with skeletal muscle adaptation to exercise training? | Merry TL, Ristow M. | 2016 | Not about recovery |
| Do antioxidant vitamins prevent exercise-induced muscle damage? a systematic review | Martinez-Ferran M, Sanchis-Gomar F, Lavie CJ, Lippi G, Pareja-Galeano H. | 2020 | Not about recovery |
| Does electrical stimulation enhance post-exercise performance recovery? | Babault N, Cometti C, Maffiuletti NA, Deley G. | 2011 | Duplicate |
| Does electrical stimulation enhance post-exercise performance recovery? | Babault N, Cometti C, Maffiuletti NA, Deley G. | 2011 | Not a systematic review or meta-analysis |
| Does exercise training improve cardiac-parasympathetic nervous system activity in sedentary people? a systematic review with meta-analysis | Lizónet AC, Rocamora AM, Flatt AA, Sarabia JM, Ramon MM. | 2022 | Not about recovery, Not focused on endurance athletes |
| Does protein supplementation support adaptations to arduous concurrent exercise training? a systematic review and meta-analysis with military based applications | Chapman S, Chung HC, Rawcliffe AJ, Izard R, Smith L, Roberts JD. | 2021 | Duplicate |
| Does protein supplementation support adaptations to arduous concurrent exercise training? a systematic review and meta-analysis with military based applications | Chapman S, Chung HC, Rawcliffe AJ, Izard R, Smith L, Roberts JD. | 2021 | Not focused on endurance athletes |
| Does vitamin c minimise exercise-induced oxidative stress? | Sukri NM. | 2021 | Not about recovery |
| Dynamic strength training intensity in cardiovascular rehabilitation: is it time to reconsider clinical practice? a systematic review | Hansen D, Abreu A, Doherty P, Völler H. | 2019 | Not about recovery |
| Eccentric training in pulmonary rehabilitation of post-covid-19 patients: an alternative for improving the functional capacity, inflammation, and oxidative stress | Contreras-Briceño F, Espinosa-Ramírez M, Rozenberg D, Reid WD. | 2022 | Duplicate |
| Eccentric training in pulmonary rehabilitation of post-covid-19 patients: an alternative for improving the functional capacity, inflammation, and oxidative stress | Contreras-Briceño F, Espinosa-Ramírez M, Rozenberg D, Reid WD. | 2022 | Not about recovery |
| Effect of active versus passive recovery on performance-related outcome during high-intensity interval exercise | Perrier-Melo RJ, D'Amorim I, Santos TM, Costa EC, Barbosa RR, Costa MD. | 2021 | Not eligible |
| Effect of caffeine ingestion on indirect markers of exercise-induced muscle damage: a systematic review of human trials | Caldas LC, Salgueiro RB, Clarke ND, Tallis J, Barauna VG, Guimaraes-Ferreira L. | 2022 | Not about recovery, Not focused on endurance athletes |
| Effect of caffeine supplementation on sports performance based on differences between sexes: a systematic review | Mielgo-Ayuso J, Marques-Jiménez D, Refoyo I, Del Coso J, León-Guereño P, Calleja-González J. | 2019 | Duplicate |
| Effect of caffeine supplementation on sports performance based on differences between sexes: a systematic review | Mielgo-Ayuso J, Marques-Jiménez D, Refoyo I, Del Coso J, León-Guereño P, Calleja-González J. | 2019 | Not about recovery |
| Effect of exercise intensity, duration and mode on post-exercise oxygen consumption | Børsheim E, Bahr R. | 2003 | Focused on physiology |
| Effect of exercise interventions in the early phase to improve physical function after hip fracture - a systematic review and meta-analysis | Beckmann M, Bruun-Olsen V, Pripp AH, Bergland A, Smith T, Heiberg KE. | 2020 | Duplicate |
| Effect of exercise interventions in the early phase to improve physical function after hip fracture - a systematic review and meta-analysis | Beckmann M, Bruun-Olsen V, Pripp AH, Bergland A, Smith T, Heiberg KE. | 2020 | Not about recovery |
| Effect of food sources of nitrate, polyphenols, l-arginine and l-citrulline on endurance exercise performance: a systematic review and meta-analysis of randomised controlled trials | d'Unienville NMA, Blake HT, Coates AM, Hill AM, Nelson MJ, Buckley JD. | 2021 | Not about recovery |
| Effect of hypoxia on muscular performance capacity: "living low--training high" | Vogt M, Billeter R, Hoppeler H. | 2003 | Not about recovery |
| Effect of progressive resistive exercise training in improving mobility and functional ability of middle adulthood patients with chronic kidney disease | Sah SK, Siddiqui MA, Darain H. | 2015 | Focused on disease |
| Effect of sports massage on performance and recovery: a systematic review and meta-analysis | Davis HL, Alabed S, Chico TJA. | 2020 | Duplicate |
| Effectiveness of community-based rehabilitation interventions incorporating outdoor mobility on ambulatory ability and falls-related self-efficacy after hip fracture: a systematic review and meta-analysis | Sheehan KJ, Fitzgerald L, Lambe K, Martin FC, Lamb SE, Sackley C. | 2021 | Duplicate |
| Effectiveness of community-based rehabilitation interventions incorporating outdoor mobility on ambulatory ability and falls-related self-efficacy after hip fracture: a systematic review and meta-analysis | Sheehan KJ, Fitzgerald L, Lambe K, Martin FC, Lamb SE, Sackley C. | 2021 | Not about recovery |
| Effectiveness of neuromuscular electrical stimulation for the rehabilitation of moderate-to-severe copd: a meta-analysis | Chen RC, Li XY, Guan LL, Guo BP, Wu WL, Zhou ZQ, Huo YT, Chen X, Zhou LQ. | 2016 | Not about recovery |
| Effectiveness of neuromuscular electrical stimulation on lower limbs of patients with hemiplegia after chronic stroke: a systematic review | Hong Z, Sui M, Zhuang Z, Liu H, Zheng X, Cai C, Jin D. | 2018 | Duplicate |
| Effectiveness of neuromuscular electrical stimulation on lower limbs of patients with hemiplegia after chronic stroke: a systematic review | Hong Z, Sui M, Zhuang Z, Liu H, Zheng X, Cai C, Jin D. | 2018 | Focused on disease |
| Effectiveness of supervised home-based exercise therapy compared to a control intervention on functions, activities, and participation in older patients after hip fracture: a systematic review and meta-analysis | Kuijlaars IAR, Sweerts L, Nijhuis-van der Sanden MWG, van Balen R, Staal JB, van Meeteren NLU, Hoogeboom TJ. | 2019 | Duplicate |
| Effectiveness of supervised home-based exercise therapy compared to a control intervention on functions, activities, and participation in older patients after hip fracture: a systematic review and meta-analysis | Kuijlaars IA, Sweerts L, Nijhuis-van der Sanden MW, van Balen R, Staal JB, van Meeteren NL, Hoogeboom TJ. | 2019 | Focused on disease |
| Effects of acute exhaustive exercise and chronic exercise training on type 1 and type 2 t lymphocytes | Lancaster GI, Halson SL, Khan Q, Drysdale P, Wallace F, Jeukendrup AE, Drayson MT, Gleeson M. | 2004 | Focused on disease |
| Effects of aerobic physical exercise on neuroplasticity after stroke: systematic review | Penna LG, Pinheiro JP, Ramalho SH, Ribeiro CF. | 2021 | Focused on disease |
| Effects of an acute exercise bout on serum hepcidin levels | Domínguez R, Sánchez-Oliver AJ, Mata-Ordoñez F, Feria-Madueño A, Grimaldi-Puyana M, López-Samanes Á, Pérez-López A. | 2018 | Duplicate |
| Effects of an acute exercise bout on serum hepcidin levels | Domínguez R, Sánchez-Oliver AJ, Mata-Ordoñez F, Feria-Madueño A, Grimaldi-Puyana M, López-Samanes Á, Pérez-López A. | 2018 | Focused on physiology |
| Effects of androgenic-anabolic steroids in athletes | Hartgens F, Kuipers H. | 2004 | Duplicate |
| Effects of androgenic-anabolic steroids in athletes | Hartgens F, Kuipers H. | 2004 | Not about recovery |
| Effects of branched amino acids in endurance sports: a review | Salinas-García ME, Martínez-Sanz JM, Urdampilleta A, Mielgo-Ayuso J, Norte Navarro A, Ortiz-Moncada R. | 2015 | Duplicate |
| Effects of branched amino acids in endurance sports: a review | Salinas-García ME, Martínez-Sanz JM, Urdampilleta A, Mielgo-Ayuso J, Norte Navarro A, Ortiz-Moncada R. | 2015 | Not a systematic review or meta-analysis |
| Effects of carbohydrate-protein beverages on recovery from exercise | Cepero Gonzalez M, Padial Ruz R, Rojas Ruiz F, Romero Sanchez D, De la Cruz Marquez J. | 2016 | Not eligible |
| Effects of cardiovascular exercise early after stroke: systematic review and meta-analysis | Stoller O, de Bruin ED, Knols RH, Hunt KJ. | 2012 | Duplicate |
| Effects of cardiovascular exercise early after stroke: systematic review and meta-analysis | Stoller O, de Bruin ED, Knols RH, Hunt KJ. | 2012 | Not about recovery |
| Effects of concurrent training on physical health and performance | Jha P, Khurana S, Ali K, Ahmad I, Verma S. | 2018 | Not about recovery |
| Effects of creatine supplementation on athletic performance in soccer players: a systematic review and meta-analysis | Mielgo-Ayuso J, Calleja-Gonzalez J, Marqués-Jiménez D, Caballero-García A, Córdova A, Fernández-Lázaro D. | 2019 | Not focused on endurance athletes |
| Effects of curcumin supplementation on sport and physical exercise: a systematic review | Suhett LG, de Miranda Monteiro Santos R, Silveira BKS, Leal ACG, de Brito ADM, de Novaes JF, Lucia CMD. | 2021 | Duplicate |
| Effects of different exercise interventions on cardiac autonomic control and secondary health factors in middle-aged adults: a systematic review | Graessler B, Thielmann B, Boeckelmann I, Hoekelmann A. | 2021 | Not about recovery, Not focused on endurance athletes |
| Effects of different training interventions on heart rate variability and cardiovascular health and risk factors in young and middle-aged adults: a systematic review | Grässler B, Thielmann B, Böckelmann I, Hökelmann A. | 2021 | Duplicate |
| Effects of different training interventions on heart rate variability and cardiovascular health and risk factors in young and middle-aged adults: a systematic review | Grässler B, Thielmann B, Böckelmann I, Hökelmann A. | 2021 | Not about recovery |
| Effects of exercise on the therapeutic and rehabilitation process of patients with multiple sclerosis- a narrative review | Saadat P, Hojjati SM, Naghshineh H, Ahangar AA, Khatir AA. | 2019 | Focused on disease |
| Effects of exercise training on autonomic function in chronic heart failure: systematic review | Hsu CY, Hsieh PL, Hsiao SF, Chien MY. | 2015 | Not about recovery |
| Effects of ingesting carbohydrate-protein supplements during exercise on endurance performance: a systematic review | Espino-González E, Candia-Lujan R. | 2015 | Not about recovery |
| Effects of ingesting protein in combination with carbohydrate during exercise on endurance performance: a systematic review with meta-analysis | Stearns RL, Emmanuel H, Volek JS, Casa DJ. | 2010 | Not about recovery |
| Effects of ketone bodies on endurance exercise | Sansone M, Sansone A, Borrione P, Romanelli F, Di Luigi L, Sgrò P. | 2018 | Duplicate |
| Effects of ketone bodies on endurance exercise | Sansone M, Sansone A, Borrione P, Romanelli F, Di Luigi L, Sgrò P. | 2018 | Not about recovery |
| Effects of physical training on endocrine functions | Duclos M. | 2001 | Not about recovery |
| Effects of plyometric jump training on repeated sprint ability in athletes: a systematic review and meta-analysis | Ramirez-Campillo R, Gentil P, Negra Y, Grgic J, Girard O. | 2021 | Not about recovery |
| Effects of pomegranate supplementation on exercise performance and post-exercise recovery in healthy adults: a systematic review | Ammar A, Bailey SJ, Chtourou H, Trabelsi K, Turki M, Hökelmann A, Souissi N. | 2018 | Duplicate |
| Effects of pre-, post- and intra-exercise hyperbaric oxygen therapy on performance and recovery: a systematic review and meta-analysis | Huang X, Wang R, Zhang Z, Wang G, Gao B. | 2021 | Duplicate |
| Effects of pre-, post- and intra-exercise hyperbaric oxygen therapy on performance and recovery: a systematic review and meta-analysis | Huang X, Wang R, Zhang Z, Wang G, Gao B. | 2021 | Not eligible |
| Effects of pregnancy on maternal work tolerance | Wolfe LA, Charlesworth SA, Glenn NM, Heenan AP, Davies GA. | 2005 | Not about recovery |
| Effects of preoperative combined aerobic and resistance exercise training in cancer patients undergoing tumour resection surgery: a systematic review of randomised trials | Piraux E, Caty G, Reychler G. | 2018 | Focused on disease |
| Effects of proprioceptive exercises on pain and function in chronic neck- and low back pain rehabilitation: a systematic literature review | McCaskey MA, Schuster-Amft C, Wirth B, Suica Z, de Bruin ED. | 2014 | Not about recovery |
| Effects of protein in combination with carbohydrate supplements on acute or repeat endurance exercise performance: a systematic review | McLellan TM, Pasiakos SM, Lieberman HR. | 2014 | Duplicate |
| Effects of protein in combination with carbohydrate supplements on acute or repeat endurance exercise performance: a systematic review | McLellan TM, Pasiakos SM, Lieberman HR. | 2014 | Not focused on endurance athletes |
| Effects of protein supplementation on performance and recovery in resistance and endurance training | Cintineo HP, Arent MA, Antonio J, Arent SM. | 2018 | Duplicate |
| Effects of protein supplementation on performance and recovery in resistance and endurance training | Cintineo HP, Arent MA, Antonio J, Arent SM. | 2018 | Not a systematic review or meta-analysis |
| Effects of protein supplements on muscle damage, soreness and recovery of muscle function and physical performance: a systematic review | Pasiakos SM, Lieberman HR, McLellan TM. | 2014 | Duplicate |
| Effects of protein supplements on muscle damage, soreness and recovery of muscle function and physical performance: a systematic review | Pasiakos SM, Lieberman HR, McLellan TM. | 2014 | Not focused on endurance athletes |
| Effects of robotic gait training after stroke: a meta-analysis | Moucheboeuf G, Griffier R, Gasq D, Glize B, Bouyer L, Dehail P, Cassoudesalle H. | 2020 | Duplicate |
| Effects of robotic gait training after stroke: a meta-analysis | Moucheboeuf G, Griffier R, Gasq D, Glize B, Bouyer L, Dehail P, Cassoudesalle H. | 2020 | Not about recovery |
| Effects of skeletal muscle energy availability on protein turnover responses to exercise | Smiles WJ, Hawley JA, Camera DM. | 2016 | Duplicate |
| Effects of skeletal muscle energy availability on protein turnover responses to exercise | Smiles WJ, Hawley JA, Camera DM. | 2016 | Focused on physiology |
| Effects of small-sided games vs. conventional endurance training on endurance performance in male youth soccer players: a meta-analytical comparison | Moran J, Blagrove RC, Drury B, Fernandes JFT, Paxton K, Chaabene H, Ramirez-Campillo R. | 2019 | Duplicate |
| Effects of small-sided games vs. conventional endurance training on endurance performance in male youth soccer players: a meta-analytical comparison | Moran J, Blagrove RC, Drury B, Fernandes JFT, Paxton K, Chaabene H, Ramirez-Campillo R. | 2019 | Not about recovery |
| Effects of transcranial direct current stimulation on walking ability after stroke: a systematic review and meta-analysis | Li Y, Fan J, Yang J, He C, Li S. | 2018 | Focused on disease |
| Effects of velocity loss threshold during resistance training on strength and athletic adaptations: a systematic review with meta-analysis | Hernandez-Belmonte A, Pallares JG. | 2022 | Not about recovery |
| Efficacy of exercise in parkinson's disease | Reuter I, Ebersbach G. | 2012 | Focused on disease |
| Efficacy of interventions to improve respiratory function after stroke | Menezes KK, Nascimento LR, Avelino PR, Alvarenga MTM, Teixeira-Salmela LF. | 2018 | Focused on disease |
| Efficacy of muscle exercise in patients with muscular dystrophy: a systematic review showing a missed opportunity to improve outcomes | Gianola S, Pecoraro V, Lambiase S, Gatti R, Banfi G, Moja L. | 2013 | Not about recovery |
| Efficacy of popular diets applied by endurance athletes on sports performance: beneficial or detrimental? a narrative review | Devrim-Lanpir A, Hill L, Knechtle B. | 2021 | Duplicate |
| Efficacy of popular diets applied by endurance athletes on sports performance: beneficial or detrimental? a narrative review | Devrim-Lanpir A, Hill L, Knechtle B. | 2021 | Not about recovery |
| Efficacy of tiotropium-olodaterol fixed-dose combination in copd | Derom E, Brusselle GG, Joos GF. | 2016 | Focused on disease |
| Eicosapentaenoic acid (epa) and docosahexaenoic acid (dha) in muscle damage and function | Ochi E, Tsuchiya Y. | 2018 | Duplicate |
| Eicosapentaenoic acid (epa) and docosahexaenoic acid (dha) in muscle damage and function | Ochi E, Tsuchiya Y. | 2018 | Focused on physiology |
| Electric fans for reducing adverse health impacts in heatwaves | Gupta S, Carmichael C, Simpson C, Clarke MJ, Allen C, Gao Y, Chan EY, Murray V. | 2012 | Not about recovery |
| Endurance assessment in handball: a systematic review | Camacho-Cardenosa A, Camacho-Cardenosa M, Brazo-Sayavera J. | 2019 | Not about recovery |
| Endurance training and cardiorespiratory conditioning after traumatic brain injury | Mossberg KA, Amonette WE, Masel BE. | 2010 | Focused on disease |
| Endurance training at altitude | Saunders PU, Pyne DB, Gore CJ. | 2009 | Duplicate |
| Endurance training at altitude | Saunders PU, Pyne DB, Gore CJ. | 2009 | Not about recovery |
| Energy availability in athletics: health, performance, and physique | Melin AK, Heikura IA, Tenforde A, Mountjoy M. | 2019 | Duplicate |
| Energy availability in athletics: health, performance, and physique | Melin AK, Heikura IA, Tenforde A, Mountjoy M. | 2019 | Not about recovery |
| Energy availability, macronutrient intake, and nutritional supplementation for improving exercise performance in endurance athletes | Casazza GA, Tovar AP, Richardson CE, Cortez AN, Davis BA. | 2018 | Duplicate |
| Energy availability, macronutrient intake, and nutritional supplementation for improving exercise performance in endurance athletes | Casazza GA, Tovar AP, Richardson CE, Cortez AN, Davis BA. | 2018 | Not about recovery |
| Energy beverages: content and safety | Higgins JP, Tuttle TD, Higgins CL. | 2010 | Duplicate |
| Energy beverages: content and safety | Higgins JP, Tuttle TD, Higgins CL. | 2010 | Not a systematic review or meta-analysis |
| Energy metabolism during endurance flight and the post-flight recovery phase | Jenni-Eiermann S. | 2017 | Duplicate |
| Energy metabolism during endurance flight and the post-flight recovery phase | Jenni-Eiermann S. | 2017 | Focused on physiology |
| Energy system contribution during competitive cross-country skiing | Losnegard T. | 2019 | Duplicate |
| Energy system contribution during competitive cross-country skiing | Losnegard T. | 2019 | Not about recovery |
| Epidural and transcutaneous spinal electrical stimulation for restoration of movement after incomplete and complete spinal cord injury | Mayr W, Krenn M, Dimitrijevic MR. | 2016 | Duplicate |
| Epidural and transcutaneous spinal electrical stimulation for restoration of movement after incomplete and complete spinal cord injury | Mayr W, Krenn M, Dimitrijevic MR. | 2016 | Not about recovery |
| Ergogenic aids in competitive handball players: a narrative review | Muñoz A, Sánchez Oliver AJ, Rivilla J, Lopez Samanes A. | 2022 | Focused on team sports, Not a systematic review or meta-analysis |
| Ergonomic interventions for preventing work‐related musculoskeletal disorders of the upper limb and neck among office workers | Hoe VC, Urquhart DM, Kelsall HL, Zamri EN, Sim MR. | 2018 | Not about recovery |
| Establishment and identification of an animal model of long-term exercise-induced fatigue | Yan K, Gao H, Liu X, Zhao Z, Gao B, Zhang L. | 2022 | Not about recovery |
| Evaluating the effects of increased protein intake on muscle strength, hypertrophy and power adaptations with concurrent training: a narrative review | Camera DM. | 2022 | Not a systematic review or meta-analysis |
| Evaluation of animal models and methods for assessing shoulder function after rotator cuff tear: a systematic review | Liu Y, Fu SC, Leong HT, Ling SK, Oh JH, Yung PS. | 2021 | Duplicate |
| Evaluation of animal models and methods for assessing shoulder function after rotator cuff tear: a systematic review | Liu Y, Fu SC, Leong HT, Ling SK, Oh JH, Yung PS. | 2021 | Not about recovery |
| Evidence review for outpatient hip and knee postoperative rehabilitation: joint replacement (primary): hip, knee and shoulder: evidence review R | National Guideline Centre (UK). | 2020 | Focused on disease |
| Evidence-based evaluation of potential benefits and safety of beta-alanine supplementation for military personnel | Ko R, Low Dog T, Gorecki DK, Cantilena LR, Costello RB, Evans WJ, Hardy ML, Jordan SA, Maughan RJ, Rankin JW, Smith-Ryan AE. | 2014 | Not focused on endurance athletes |
| Evidence-based post-exercise recovery strategies in rugby: a narrative review | Calleja-González J, Mielgo-Ayuso J, Ostojic SM, Jones MT, Marques-Jiménez D, Caparros T, Terrados N. | 2019 | Duplicate |
| Evidence-based post-exercise recovery strategies in rugby: a narrative review | Calleja-González J, Mielgo-Ayuso J, Ostojic SM, Jones MT, Marques-Jiménez D, Caparros T, Terrados N. | 2019 | Not focused on endurance athletes |
| Evidence-based recommendations for optimal dietary protein intake in older people: a position paper from the prot-age study group | Bauer J, Biolo G, Cederholm T, Cesari M, Cruz-Jentoft AJ, Morley JE, Phillips S, Sieber C, Stehle P, Teta D, Visvanathan R, Volpi E, Boirie Y. | 2013 | Not focused on endurance athletes |
| Evidence-based rehabilitation of mobility after stroke | Dohle C, Tholen R, Wittenberg H, Quintern J, Saal S, Stephan KM. | 2016 | Focused on disease |
| Excess of exercise increases the risk of atrial fibrillation | Müssigbrodt A, Weber A, Mandrola J, van Belle Y, Richter S, Döring M, Arya A, Sommer P, Bollmann A, Hindricks G. | 2017 | Not about recovery |
| Exercise and autonomic function in health and cardiovascular disease | Rosenwinkel ET, Bloomfield DM, Arwady MA, Goldsmith RL. | 2001 | Focused on disease |
| Exercise and functional foods | Aoi W, Naito Y, Yoshikawa T. | 2006 | Not about recovery |
| Exercise and heart transplantation. a review | Niset G, Hermans L, Depelchin P. | 1991 | Duplicate |
| Exercise and heart-transplantation - a review | Niset G, Hermans L, Depelchin P. | 1991 | Not about recovery |
| Exercise and immune function. recent developments | Nieman DC, Pedersen BK. | 1999 | Focused on physiology |
| Exercise and injury prevention in older people | Skelton DA, Beyer N. | 2003 | Not about recovery |
| Exercise and neuroplasticity in persons living with parkinson's disease | Hirsch MA, Farley BG. | 2009 | Focused on disease |
| Exercise and the regulation of inflammatory responses | Allen J, Sun Y, Woods JA. | 2015 | Not about recovery |
| Exercise and training to optimize functional motor performance in stroke: driving neural reorganization? | Shepherd RB. | 2001 | Focused on disease |
| Exercise for people with peripheral neuropathy | White CM, Pritchard J, Turner-Stokes L. | 2004 | Focused on disease |
| Exercise for the low back pain patient | Jenkins EM, Borenstein DG. | 1994 | Not about recovery |
| Exercise for treating patellofemoral pain syndrome | van der Heijden RA, Lankhorst NE, van Linschoten R, Bierma‐Zeinstra SM, van Middelkoop M. | 2015 | Focused on disease |
| Exercise in heart failure - additional effect to medication? | Seebach R, Hensler D, Christle JW, Pressler A, Halle M. | 2011 | Focused on disease |
| Exercise induced respiratory muscle fatigue - a review of methodology and recent findings | Ozkaplan A, Rhodes EC. | 2004 | Not about recovery |
| Exercise intervention studies in patients with peripheral neuropathy: a systematic review | Streckmann F, Zopf EM, Lehmann HC, May K, Rizza J, Zimmer P, Gollhofer A, Bloch W, Baumann FT. | 2014 | Duplicate |
| Exercise intervention studies in patients with peripheral neuropathy: a systematic review | Streckmann F, Zopf EM, Lehmann HC, May K, Rizza J, Zimmer P, Gollhofer A, Bloch W, Baumann FT. | 2014 | Focused on disease |
| Exercise prehabilitation in lung cancer: getting stronger to recover faster | Avancini A, Cavallo A, Trestini I, Tregnago D, Belluomini L, Crisafulli E, Micheletto C, Milella M, Pilotto S, Lanza M, Infante MV. | 2011 | Focused on disease |
| Exercise prior to influenza vaccination for limiting influenza incidence and its related complications in adults | Grande AJ, Reid H, Thomas EE, Nunan D, Foster C. | 2016 | Focused on disease |
| Exercise rehabilitation following intensive care unit discharge for recovery from critical illness | Connolly B, Salisbury L, O'Neill B, Geneen L, Douiri A, Grocott MP, Hart N, Walsh TS, Blackwood B and ERACIP Group. | 2015 | Duplicate |
| Exercise rehabilitation following intensive care unit discharge for recovery from critical illness | Connolly B, Salisbury L, O'Neill B, Geneen L, Douiri A, Grocott MP, Hart N, Walsh TS, Blackwood B and ERACIP Group. | 2015 | Focused on disease |
| Exercise therapy in patients with idiopathic inflammatory myopathies and systemic lupus erythematosus - a systematic literature review | Alexanderson H, Boström C. | 2020 | Focused on disease |
| Exercise training and interventions for coronary artery disease | Fernández-Rubio H, Becerro-de-Bengoa-Vallejo R, Rodríguez-Sanz D, Calvo-Lobo C, Vicente-Campos D, Chicharro JL. | 2022 | Focused on disease |
| Exercise training and protein metabolism: influences of contraction, protein intake, and sex-based differences | Burd NA, Tang JE, Moore DR, Phillips SM. | 2009 | Duplicate |
| Exercise training and protein metabolism: influences of contraction, protein intake, and sex-based differences | Burd NA, Tang JE, Moore DR, Phillips SM. | 2009 | Not about recovery |
| Exercise training as treatment in cancer cachexia | Lira FS, Neto JC, Seelaender M. | 2014 | Duplicate |
| Exercise training as treatment in cancer cachexia | Lira FS, Neto JC, Seelaender M. | 2014 | Not about recovery |
| Exercise training for intermittent claudication | McDermott MM. | 2017 | Not about recovery |
| Exercise versus no exercise for the occurrence, severity, and duration of acute respiratory infections | Grande AJ, Keogh J, Silva V, Scott AM. | 2020 | Focused on disease |
| Exercise-induced cardiac fatiguem - a review of the echocardiographic literature | Oxborough D, Birch K, Shave R, George K. | 2010 | Not about recovery |
| Exercise-induced dna damage: is there a relationship with inflammatory responses? | Neubauer O, Reichhold S, Nersesyan A, König D, Wagner KH. | 2008 | Duplicate |
| Exercise-induced dna damage: is there a relationship with inflammatory responses? | Neubauer O, Reichhold S, Nersesyan A, König D, Wagner KH. | 2008 | Focused on physiology |
| Exercise-induced illness and inflammation: can immunonutrition and iron help? | Castell LM, Nieman DC, Bermon S, Peeling P. | 2019 | Duplicate |
| Exercise-induced illness and inflammation: can immunonutrition and iron help? | Castell LM, Nieman DC, Bermon S, Peeling P. | 2019 | Not focused on endurance athletes |
| Exercise-induced muscle damage: mechanism, assessment and nutritional factors to accelerate recovery | Markus I, Constantini K, Hoffman JR, Bartolomei S, Gepner Y. | 2021 | Duplicate |
| Exercise-induced muscle damage: mechanism, assessment and nutritional factors to accelerate recovery | Markus I, Constantini K, Hoffman JR, Bartolomei S, Gepner Y. | 2021 | Focused on physiology |
| Exercise-induced neuroprotection and recovery of motor function in animal models of parkinson's disease | Palasz E, Niewiadomski W, Gasiorowska A, Wysocka A, Stepniewska A, Niewiadomska G. | 2019 | Focused on disease |
| Exercise, ageing and the lung | Roman MA, Rossiter HB, Casaburi R. | 2016 | Not about recovery |
| Exercise, nutrition, and supplements in the muscle carnitine palmitoyl-transferase ii deficiency: new theoretical bases for potential applications | Negro M, Cerullo G, Parimbelli M, Ravazzani A, Feletti F, Berardinelli A, Cena H, D'Antona G. | 2021 | Duplicate |
| Exercise, nutrition, and supplements in the muscle carnitine palmitoyl-transferase ii deficiency: new theoretical bases for potential applications | Negro M, Cerullo G, Parimbelli M, Ravazzani A, Feletti F, Berardinelli A, Cena H, D'Antona G. | 2021 | Not about recovery |
| Exercising with a denervated heart after cardiac transplantation | Mettauer B, Levy F, Richard R, Roth O, Zoll J, Lampert E, Lonsdorfer J, Geny B. | 2005 | Focused on disease |
| Exergaming for physical therapy in patients with down syndrome: a systematic review and meta-analysis of randomized-controlled trials | Alba-Rueda A, Moral-Munoz JA, De Miguel-Rubio A, Lucena-Anton D. | 2022 | Duplicate |
| Exergaming for physical therapy in patients with down syndrome: a systematic review and meta-analysis of randomized-controlled trials | Alba-Rueda A, Moral-Munoz JA, De Miguel-Rubio A, Lucena-Anton D. | 2022 | Not about recovery |
| Exertional heat illness: emerging concepts and advances in prehospital care | Pryor RR, Roth RN, Suyama J, Hostler D. | 2015 | Focused on disease |
| Exogenous ketone supplements in athletic contexts: past, present, and future | Evans M, McClure TS, Koutnik AP, Egan B. | 2022 | Duplicate |
| Exogenous ketone supplements in athletic contexts: past, present, and future | Evans M, McClure TS, Koutnik AP, Egan B. | 2022 | Focused on physiology |
| Experiences of taking neuroleptic medication and impacts on symptoms, sense of self and agency: a systematic review and thematic synthesis of qualitative data | Thompson J, Stansfeld JL, Cooper RE, Morant N, Crellin NE, Moncrieff J. | 2020 | Not about recovery |
| Extreme conditioning programs: potential benefits and potential risks | Knapik JJ. | 2015 | Not about recovery |
| Facilitation of descending excitatory and spinal inhibitory networks from training of endurance and precision walking in participants with incomplete spinal cord injury | Zewdie ET, Roy FD, Yang JF, Gorassini MA. | 2015 | Not about recovery |
| Factors affecting the rate of phosphocreatine resynthesis following intense exercise | McMahon S, Jenkins D. | 2002 | Not about recovery, Focused on physiology |
| Factors that influence the performance of elite sprint cross-country skiers | Hébert-Losier K, Zinner C, Platt S, Stöggl T, Holmberg HC. | 2017 | Duplicate |
| Factors that influence the performance of elite sprint cross-country skiers | Hébert-Losier K, Zinner C, Platt S, Stöggl T, Holmberg HC. | 2017 | Not about recovery |
| Family therapy approaches for anorexia nervosa | Fisher CA, Skocic S, Rutherford KA, Hetrick SE. | 2019 | Focused on disease |
| Fasting and recovery from exercise | Burke L. | 2010 | Not a systematic review or meta-analysis |
| Fatigability during volitional walking in incomplete spinal cord injury: cardiorespiratory and motor performance considerations | Gollie JM. | 2018 | Not about recovery |
| Fatigue and illness in athletes | Nimmo MA, Ekblom B and International Association of Athletics Federations. | 2007 | Not about recovery |
| Fatigue and underperformance in athletes: the overtraining syndrome | Budgett R. | 1998 | Not about recovery |
| Fatigue management in the preparation of olympic athletes | Robson-Ansley PJ, Gleeson M, Ansley L. | 2009 | Not about recovery |
| Feasibility and effectiveness of repetitive gait training early after stroke: a systematic review and meta-analysis | Schröder J, Truijen S, Van Criekinge T, Saeys W. | 2019 | Duplicate |
| Feasibility and effectiveness of repetitive gait training early after stroke: a systematic review and meta-analysis | Schröder J, Truijen S, Van Criekinge T, Saeys W. | 2019 | Focused on disease |
| Field-based tests for the assessment of physical fitness in children and adolescents practicing sport: a systematic review within the esa program | Tabacchi G, Lopez Sanchez GF, Nese Sahin F, Kizilyalli M, Genchi R, Basile M, Kirkar M, Silva C, Loureiro N, Teixeira E, Demetriou Y. | 2019 | Not about recovery |
| Fingertip injuries | Kotkansalo T. | 2012 | Not about recovery |
| Fitness training for cardiorespiratory conditioning after traumatic brain injury | Hassett L, Moseley AM, Harmer AR. | 2017 | Focused on disease |
| Fluid and electrolyte balance in ultra-endurance sport | Rehrer NJ. | 2001 | Focused on physiology |
| From tusko to titin: the role for comparative physiology in an era of molecular discovery | Lindstedt SL, Nishikawa KC. | 2015 | Not about recovery, Focused on physiology |
| Fructose co-ingestion to increase carbohydrate availability in athletes | Fuchs CJ, Gonzalez JT, van Loon LJC. | 2019 | Duplicate |
| Fructose co-ingestion to increase carbohydrate availability in athletes | Fuchs CJ, Gonzalez JT, van Loon LJC. | 2019 | Not about recovery |
| Fruit for sport | Naderi A, Rezaei S, Moussa A, Levers K, Earnest CP. | 2018 | Not a systematic review or meta-analysis |
| Fruit-derived polyphenol supplementation for athlete recovery and performance | Bowtell J, Kelly V. | 2019 | Duplicate |
| Fruit-derived polyphenol supplementation for athlete recovery and performance | Bowtell J, Kelly V. | 2019 | Not a systematic review or meta-analysis |
| Fueling and recovery | Patton K. | 2019 | Duplicate |
| Fueling and recovery | Patton K. | 2019 | Not a systematic review or meta-analysis |
| Fueling the rugby player: maximizing performance on and off the pitch | Casiero D. | 2013 | Not about recovery |
| Functional evaluation of the lung resection candidate | Bolliger CT, Perruchoud AP. | 1998 | Focused on disease |
| Functional impact of post-exercise cooling and heating on recovery and training adaptations: application to resistance, endurance, and sprint exercise | Chaillou T, Treigyte V, Mosely S, Brazaitis M, Venckunas T, Cheng AJ. | 2022 | Duplicate |
| Functional impact of post-exercise cooling and heating on recovery and training adaptations: application to resistance, endurance, and sprint exercise | Chaillou T, Treigyte V, Mosely S, Brazaitis M, Venckunas T, Cheng AJ. | 2022 | Not a systematic review or meta-analysis |
| Functional overreaching in endurance athletes: a necessity or cause for concern? | Bellinger P. | 2020 | Not about recovery |
| Functional, structural and molecular plasticity of mammalian skeletal muscle in response to exercise stimuli | Fluck M. | 2006 | Not about recovery |
| Fundamentals of glycogen metabolism for coaches and athletes | Murray B, Rosenbloom C. | 2018 | Not about recovery |
| Gastrointestinal complaints during exercise: prevalence, etiology, and nutritional recommendations | de Oliveira EP, Burini RC, Jeukendrup A. | 2014 | Duplicate |
| Gastrointestinal complaints during exercise: prevalence, etiology, and nutritional recommendations | de Oliveira EP, Burini RC, Jeukendrup A. | 2014 | Not about recovery |
| Gender-specific prediction of cardiac disease: importance of risk factors and exercise variables | D'Amore S, Mora S. | 2006 | Focused on disease |
| Ginger (zingiber officinale) as an analgesic and ergogenic aid in sport: a systemic review | Wilson PB. | 2015 | Duplicate |
| Ginger (zingiber officinale) as an analgesic and ergogenic aid in sport: a systemic review | Wilson PB. | 2015 | Not focused on endurance athletes |
| Global trends and hot topics in electrical stimulation of skeletal muscle research over the past decade: a bibliometric analysis | Huang Y, Gong Y, Liu Y, Lu J. | 2022 | Not about recovery |
| Glucose plus fructose ingestion for post-exercise recovery-greater than the sum of its parts? | Gonzalez JT, Fuchs CJ, Betts JA, Van Loon LJ. | 2017 | Duplicate |
| Glutamine as an aid in the recovery of muscle strength: systematic review of literature | Hernández Valencia SE, Méndez Sánchez L, Clark P, Moreno Altamirano L, Mejía Aranguré JM. | 2015 | Duplicate |
| Glutamine as an aid in the recovery of muscle strength: systematic review of literature | Hernández Valencia SE, Méndez Sánchez L, Clark P, Moreno Altamirano L, Mejía Aranguré JM. | 2015 | Not about recovery |
| Glutamine, exercise and immune function - links and possible mechanisms | Walsh NP, Blannin AK, Robson PJ, Gleeson M. | 1998 | Focused on physiology |
| Glutamine, exercise and immune function. links and possible mechanisms | Walsh NP, Blannin AK, Robson PJ, Gleeson M. | 1998 | Duplicate |
| Glutamine: the nonessential amino acid for performance enhancement | Phillips GC. | 2007 | Not about recovery |
| Glycaemic index and optimal performance | Walton P, Rhodes EC. | 1997 | Not about recovery |
| Glycemic index and endurance performance | Donaldson CM, Perry TL, Rose MC. | 2010 | Duplicate |
| Glycemic index and endurance performance | Donaldson CM, Perry TL, Rose MC. | 2010 | Focused on physiology |
| Glycemic index in sport nutrition | Mondazzi L, Arcelli E. | 2009 | Duplicate |
| Glycemic index in sport nutrition | Mondazzi L, Arcelli E. | 2009 | Not about recovery |
| Glycogen availability and skeletal muscle adaptations with endurance and resistance exercise | Knuiman P, Hopman MT, Mensink M. | 2015 | Duplicate |
| Glycogen availability and skeletal muscle adaptations with endurance and resistance exercise | Knuiman P, Hopman MT, Mensink M. | 2015 | Not about recovery |
| Glycopyrronium bromide for the treatment of chronic obstructive pulmonary disease | Riario-Sforza GG, Ridolo E, Riario-Sforza E, Incorvaia C. | 2015 | Focused on disease |
| Growth hormone-insulin-like growth factor axis, thyroid axis, prolactin, and exercise | Hackney AC, Davis HC, Lane AR. | 2016 | Focused on physiology |
| Guidelines for optimal replacement beverages for different athletic events | Gisolfi CV, Duchman SM. | 1992 | Not a systematic review or meta-analysis |
| Guidelines to classify subject groups in sport-science research | De Pauw K, Roelands B, Cheung SS, De Geus B, Rietjens G, Meeusen R. | 2013 | Not about recovery |
| Health and performance consequences of relative energy deficiency in sport (red-s) | Vardardottir B, Gudmundsdottir SL, Olafsdottir AS. | 2020 | Not about recovery |
| Health challenges in long-distance dog sled racing: a systematic review of literature | Calogiuri G, Weydahl A. | 2017 | Not about recovery |
| Health- and performance-related potential of resistance training | Stone MH, Fleck SJ, Triplett NT, Kraemer WJ. | 1991 | Duplicate |
| Health-related and performance-related potential of resistance training | Stone MH, Fleck SJ, Triplett NT, Kraemer WJ. | 1991 | Not about recovery |
| Heart rate monitoring in soccer: interest and limits during competitive match play and training, practical application | Alexandre D, Da Silva CD, Hill-Haas S, Wong DP, Natali AJ, De Lima JR, Bara Filho MG, Marins JJ, Garcia ES, Karim C. | 2012 | Not about recovery |
| Heart rate variability and physical exercise. current status | Hottenrott K, Hoos O, Esperer HD. | 2006 | Focused on physiology |
| Heart rate variability-guided training for enhancing cardiac-vagal modulation, aerobic fitness, and endurance performance: a methodological systematic review with meta-analysis | Manresa-Rocamora A, Sarabia JM, Javaloyes A, Flatt AA, Moya-Ramon M. | 2021 | Duplicate |
| Heart rate variability-guided training for enhancing cardiac-vagal modulation, aerobic fitness, and endurance performance: a methodological systematic review with meta-analysis | Manresa-Rocamora A, Sarabia JM, Javaloyes A, Flatt AA, Moya-Ramon M. | 2021 | Not about recovery |
| Heart rate-based indices to detect parasympathetic hyperactivity in functionally overreached athletes. a meta-analysis | Manresa-Rocamora A, Flatt AA, Casanova-Lizón A, Ballester-Ferrer JA, Sarabia JM, Vera-Garcia FJ, Moya-Ramón M. | 2021 | Duplicate |
| Heart rate-based indices to detect parasympathetic hyperactivity in functionally overreached athletes. a meta-analysis | Manresa-Rocamora A, Flatt AA, Casanova-Lizón A, Ballester-Ferrer JA, Sarabia JM, Vera-Garcia FJ, Moya-Ramón M. | 2021 | Focused on physiology |
| Hesperidin functions as an ergogenic aid by increasing endothelial function and decreasing exercise-induced oxidative stress and inflammation, thereby contributing to improved exercise performance | Imperatrice M, Cuijpers I, Troost FJ, Sthijns MMJPE. | 2022 | Duplicate |
| Hesperidin functions as an ergogenic aid by increasing endothelial function and decreasing exercise-induced oxidative stress and inflammation, thereby contributing to improved exercise performance | Imperatrice M, Cuijpers I, Troost FJ, Sthijns MMJPE. | 2022 | Not about recovery |
| High responders and low responders: factors associated with individual variation in response to standardized training | Mann TN, Lamberts RP, Lambert MI. | 2014 | Not about recovery |
| High-intensity intermittent activities at school: controversies and facts | Ratel S, Lazaar N, Dore E, Baquet G, Williams CA, Berthoin S, Van Praagh E, Bedu M, Duche P. | 2004 | Duplicate |
| High-intensity intermittent activities at school: controversies and facts | Ratel S, Lazaar N, Dore E, Baquet G, Williams CA, Berthoin S, Van Praagh E, Bedu M, Duche P. | 2004 | Not about recovery |
| High-intensity intermittent exercise: methodological and physiological aspects | Tschakert G, Hofmann P. | 2013 | Not about recovery, Focused on physiology |
| High-intensity interval training in the sports therapy of overweight and obese adolescents: a review | Engel FA, Oestreich J, Donath L, Kunz P, Sperlich B. | 2019 | Not about recovery |
| High-intensity interval training shock microcycle for enhancing sport performance: a brief review | Dolci F, Kilding AE, Chivers P, Piggott B, Hart NH. | 2020 | Not a systematic review or meta-analysis |
| High-intensity interval training, solutions to the programming puzzle. part ii: anaerobic energy, neuromuscular load and practical applications | Buchheit M, Laursen PB. | 2013 | Not about recovery |
| High-intensity training in football | Iaia FM, Rampinini E, Bangsbo J. | 2009 | Duplicate |
| High-intensity training in football | Iaia FM, Rampinini E, Bangsbo J. | 2009 | Not about recovery |
| How can we minimize the risks by optimizing patient's condition shortly before thoracic surgery? | Ellenberger C, Schorer R, Bedat B, Hagerman A, Triponez F, Karenovics W, Licker M. | 2021 | Duplicate |
| How can we minimize the risks by optimizing patient's condition shortly before thoracic surgery? | Ellenberger C, Schorer R, Bedat B, Hagerman A, Triponez F, Karenovics W, Licker M. | 2021 | Focused on disease |
| Hyperbaric oxygen therapy for delayed onset muscle soreness and closed soft tissue injury | Bennett MH, Best TM, Babul‐Wellar S, Taunton JE. | 2005 | Focused on physiology |
| Immunological parameters and upper respiratory tract infections in team sports athletes | Dias R, Baganha RJ, Cieslak F, Krinski K, Camarço NF, Verlengia R, Lopes CR, Prestes J, Silva CD, Cavaglieri CR. | 2017 | Not focused on endurance athletes |
| Impact of cardiac rehabilitation and exercise training programs in coronary heart disease | Kachur S, Chongthammakun V, Lavie CJ, De Schutter A, Arena R, Milani RV, Franklin BA. | 2017 | Focused on disease |
| Impact of cold-water immersion compared with passive recovery following a single bout of strenuous exercise on athletic performance in physically active participants: a systematic review with meta-analysis and meta-regression | Moore E, Fuller JT, Buckley JD, Saunders S, Halson SL, Broatch JR, Bellenger CR. | 2002 | Duplicate |
| Impact of cow's milk intake on exercise performance and recovery of muscle function: a systematic review | Alcantara JMA, Sanchez-Delgado G, Martinez-Tellez B, Labayen I, Ruiz JR. | 2019 | Duplicate |
| Impact of extreme exercise at high altitude on oxidative stress in humans | Quindry J, Dumke C, Slivka D, Ruby B. | 2016 | Focused on physiology |
| Impact of reduced training on performance in endurance athletes | Houmard JA. | 1991 | Duplicate |
| Impact of reduced training on performance in endurance athletes | Houmard JA. | 1991 | Not about recovery |
| Implications of impaired endurance performance following single bouts of resistance training: an alternate concurrent training perspective | Doma K, Deakin GB, Bentley DJ. | 2017 | Duplicate |
| Implications of impaired endurance performance following single bouts of resistance training: an alternate concurrent training perspective | Doma K, Deakin GB, Bentley DJ. | 2017 | Not about recovery |
| Importance of characteristics and modalities of physical activity and exercise in the management of cardiovascular health in individuals with cardiovascular disease (part iii) | Vanhees L, Geladas N, Hansen D, Kouidi E, Niebauer J, Reiner Ž, Cornelissen V, Adamopoulos S, Prescott E, Börjesson M. | 2012 | Focused on disease |
| Incorporating internal and external training load measurements in clinical decision making after acl reconstruction: a clinical commentary | Taylor JB, Ford KR, Queen RM, Owen EC, Gisselman AS. | 2021 | Not about recovery |
| Increasing self-directed training in neurorehabilitation patients through competition | Studer B, Van Dijk H, Handermann R, Knecht S. | 2016 | Not about recovery |
| Individual‐, family‐, and school‐level interventions targeting multiple risk behaviours in young people | MacArthur G, Caldwell DM, Redmore J, Watkins SH, Kipping R, White J, Chittleborough C, Langford R, Er V, Lingam R, Pasch K. | 2018 | Not about recovery |
| Inflammation and immune function: can antioxidants help the endurance athlete? | Elkington LJ, Gleeson M, Pyne DB, Callister R, Wood LG. | 2015 | Not a systematic review or meta-analysis |
| Influence of acute and chronic exercise on abdominal fat lipolysis: an update | Laurens C, De Glisezinski I, Larrouy D, Harant I, Moro C. | 2020 | Not about recovery |
| Influence of physical exercise on traumatic brain injury deficits: scaffolding effect | Archer T. | 2012 | Duplicate |
| Influence of physical exercise on traumatic brain injury deficits: scaffolding effect | Archer T. | 2012 | Focused on disease |
| Influence of prolonged physical exercise on plasma volume, plasma proteins, electrolytes, and fluid-regulating hormones | Röcker L, Kirsch KA, Heyduck B, Altenkirch HU. | 1989 | Not about recovery |
| Influences of exercise and training on the circulating concentration of prolactin in humans | Rojas Vega S, Hollmann W, Strüder HK. | 2012 | Not about recovery |
| Ingestion of carbohydrate during recovery in exercising people | Mitchell JB. | 2013 | Not a systematic review or meta-analysis |
| Injection therapies for achilles tendinopathy | Kearney RS, Parsons N, Metcalfe D, Costa ML. | 2015 | Focused on disease |
| Inpatient versus outpatient care, partial hospitalisation and waiting list for people with eating disorders | Hay PJ, Touyz S, Claudino AM, Lujic S, Smith CA, Madden S. | 2019 | Focused on disease |
| Inspiratory muscle training for the recovery of function after stroke | Xiao Y, Luo M, Wang J, Luo H. | 2012 | Focused on disease |
| Inspiratory muscle training in heart disease and heart failure: a review of the literature with a focus on method of training and outcomes | Cahalin LP, Arena R, Guazzi M, Myers J, Cipriano G, Chiappa G, Lavie CJ, Forman DE. | 2013 | Focused on disease |
| Institutional stability in management practice and industrial relations: the influence of the anglo-american council for productivity, 1948-52 | Clark I. | 1999 | Not about recovery |
| Intake of branched chain amino acids favors post-exercise muscle recovery and may improve muscle function: optimal dosage regimens and consumption conditions | Arroyo-Cerezo A, Cerrillo I, Ortega Á, Fernández-Pachón MS. | 2021 | Duplicate |
| Intake of branched chain amino acids favors post-exercise muscle recovery and may improve muscle function: optimal dosage regimens and consumption conditions | Arroyo-Cerezo A, Cerrillo I, Ortega Á, Fernández-Pachón MS. | 2021 | Not eligible |
| Intense training: the key to optimal performance before and during the taper | Mujika I. | 2010 | Duplicate |
| Intense training: the key to optimal performance before and during the taper | Mujika I. | 2010 | Not about recovery |
| Intensive care unit-acquired weakness and the covid-19 pandemic: a clinical review | Qin ES, Hough CL, Andrews J, Bunnell AE. | 2022 | Duplicate |
| Intensive care unit-acquired weakness and the covid-19 pandemic: a clinical review | Qin ES, Hough CL, Andrews J, Bunnell AE. | 2022 | Not about recovery |
| Interference between concurrent resistance and endurance exercise: molecular bases and the role of individual training variables | Fyfe JJ, Bishop DJ, Stepto NK. | 2014 | Duplicate |
| Interference between concurrent resistance and endurance exercise: molecular bases and the role of individual training variables | Fyfe JJ, Bishop DJ, Stepto NK. | 2014 | Not about recovery |
| International society of sports nutrition position stand: nutrient timing | Kerksick CM, Arent S, Schoenfeld BJ, Stout JR, Campbell B, Wilborn CD, Taylor L, Kalman D, Smith-Ryan AE, Kreider RB, Willoughby D, Arciero PJ, VanDusseldorp TA, Ormsbee MJ, Wildman R, Greenwood M, Ziegenfuss TN, Aragon AA, Antonio J. | 2008 | Duplicate |
| International society of sports nutrition position stand: nutrient timing | Kerksick CM, Arent S, Schoenfeld BJ, Stout JR, Campbell B, Wilborn CD, Taylor L, Kalman D, Smith-Ryan AE, Kreider RB, Willoughby D, Arciero PJ, VanDusseldorp TA, Ormsbee MJ, Wildman R, Greenwood M, Ziegenfuss TN, Aragon AA, Antonio J. | 2008 | Not a systematic review or meta-analysis |
| International society of sports nutrition position stand: nutritional considerations for single-stage ultra-marathon training and racing | Tiller NB, Roberts JD, Beasley L, Chapman S, Pinto JM, Smith L, Wiffin M, Russell M, Sparks SA, Duckworth L, O'Hara J, Sutton L, Antonio J, Willoughby DS, Tarpey MD, Smith-Ryan AE, Ormsbee MJ, Astorino TA, Kreider RB, McGinnis GR, Stout JR, Smith JW, Arent SM, Campbell BI, Bannock L. | 2019 | Duplicate |
| International society of sports nutrition position stand: nutritional considerations for single-stage ultra-marathon training and racing | Tiller NB, Roberts JD, Beasley L, Chapman S, Pinto JM, Smith L, Wiffin M, Russell M, Sparks SA, Duckworth L, O'Hara J, Sutton L, Antonio J, Willoughby DS, Tarpey MD, Smith-Ryan AE, Ormsbee MJ, Astorino TA, Kreider RB, McGinnis GR, Stout JR, Smith JW, Arent SM, Campbell BI, Bannock L. | 2019 | Not a systematic review or meta-analysis |
| International society of sports nutrition position stand: protein and exercise | Jäger R, Kerksick CM, Campbell BI, Cribb PJ, Wells SD, Skwiat TM, Purpura M, Ziegenfuss TN, Ferrando AA, Arent SM, Smith-Ryan AE, Stout JR, Arciero PJ, Ormsbee MJ, Taylor LW, Wilborn CD, Kalman DS, Kreider RB, Willoughby DS, Hoffman JR, Krzykowski JL, Antonio J. | 2017 | Duplicate |
| International society of sports nutrition position stand: protein and exercise | Jäger R, Kerksick CM, Campbell BI, Cribb PJ, Wells SD, Skwiat TM, Purpura M, Ziegenfuss TN, Ferrando AA, Arent SM, Smith-Ryan AE, Stout JR, Arciero PJ, Ormsbee MJ, Taylor LW, Wilborn CD, Kalman DS, Kreider RB, Willoughby DS, Hoffman JR, Krzykowski JL, Antonio J. | 2017 | Not a systematic review or meta-analysis |
| International society of sports nutrition position stand: sodium bicarbonate and exercise performance | Grgic J, Pedisic Z, Saunders B, Artioli GG, Schoenfeld BJ, McKenna MJ, Bishop DJ, Kreider RB, Stout JR, Kalman DS, Arent SM. | 2021 | Not about recovery |
| Internet‐based cognitive and behavioural therapies for post‐traumatic stress disorder (ptsd) in adults | Simon N, Robertson L, Lewis C, Roberts NP, Bethell A, Dawson S, Bisson JI. | 2021 | Not about recovery |
| Interval training for performance: a scientific and empirical practice. special recommendations for middle- and long-distance running. part i: aerobic interval training | Billat LV. | 2001 | Not about recovery |
| Interventions for cellulitis and erysipelas | Kilburn SA, Featherstone P, Higgins B, Brindle R. | 2010 | Focused on disease |
| Interventions for central serous chorioretinopathy: a network meta‐analysis | Salehi M, Wenick AS, Law HA, Evans JR, Gehlbach P. | 2015 | Focused on disease |
| Interventions for improving mobility after hip fracture surgery in adults | Fairhall NJ, Dyer SM, Mak JC, Diong J, Kwok WS, Sherrington C. | 2022 | Duplicate |
| Interventions for improving mobility after hip fracture surgery in adults | Fairhall NJ, Dyer SM, Mak JC, Diong J, Kwok WS, Sherrington C. | 2022 | Focused on disease |
| Interventions for preventing and treating stress fractures and stress reactions of bone of the lower limbs in young adults | Rome K, Handoll HH, Ashford RL. | 2005 | Focused on disease |
| Interventions for promoting habitual exercise in people living with and beyond cancer | Turner RR, Steed L, Quirk H, Greasley RU, Saxton JM, Taylor SJ, Rosario DJ, Thaha MA, Bourke L. | 2013 | Duplicate |
| Interventions for promoting habitual exercise in people living with and beyond cancer | Bourke L, Homer KE, Thaha MA, Steed L, Rosario DJ, Robb KA, Saxton JM, Taylor SJ. | 2013 | Duplicate |
| Interventions for promoting habitual exercise in people living with and beyond cancer | Bourke L, Homer KE, Thaha MA, Steed L, Rosario DJ, Robb KA, Saxton JM, Taylor SJ. | 2013 | Focused on disease |
| Interventions for promoting habitual exercise in people living with and beyond cancer | Turner RR, Steed L, Quirk H, Greasley RU, Saxton JM, Taylor SJ, Rosario DJ, Thaha MA, Bourke L. | 2013 | Focused on disease |
| Interventions for the prevention and treatment of pes cavus | Burns J, Landorf KB, Ryan MM, Crosbie J, Ouvrier RA. | 2007 | Focused on disease |
| Interventions for treating acute elbow dislocations in adults | Taylor F, Sims M, Theis JC, Herbison GP. | 2012 | Focused on disease |
| Interventions for treating chronic ankle instability | de Vries JS, Krips R, Sierevelt IN, Blankevoort L, Van Dijk CN. | 2011 | Focused on disease |
| Intramuscular mechanisms of overtraining | Cheng AJ, Jude B, Lanner JT. | 2020 | Focused on physiology |
| Iron and physical activity: bioavailability enhancers, properties of black pepper (bioperine(®)) and potential applications | Fernández-Lázaro D, Mielgo-Ayuso J, Córdova Martínez A, Seco-Calvo J. | 2020 | Duplicate |
| Iron and physical activity: bioavailability enhancers, properties of black pepper (bioperine(®)) and potential applications | Fernández-Lázaro D, Mielgo-Ayuso J, Córdova Martínez A, Seco-Calvo J. | 2020 | Not about recovery |
| Is -hydroxy -methylbutyrate an effective anabolic agent to improve outcome in older diseased populations? | Engelen MP, Deutz NE, Mostert R, Wouters EF, Schols AM. | 2018 | Focused on disease |
| Is high-intensity interval training suitable to promote neuroplasticity and cognitive functions after stroke? | Hugues N, Pellegrino C, Rivera C, Berton E, Pin-Barre C, Laurin J. | 2021 | Focused on disease |
| Is it time to turn our attention toward central mechanisms for post-exertional recovery strategies and performance? | Rattray B, Argus C, Martin K, Northey J, Driller M. I | 2015 | Not a systematic review or meta-analysis |
| Is there a specific role for sucrose in sports and exercise performance? | Wallis GA, Wittekind A. | 2013 | Not a systematic review or meta-analysis |
| Is there evidence that runners can benefit from wearing compression clothing? | Engel FA, Holmberg HC, Sperlich B. | 2016 | Duplicate |
| Is β-hydroxy β-methylbutyrate an effective anabolic agent to improve outcome in older diseased populations? | Engelen MPKJ, Deutz NEP. | 2018 | Duplicate |
| Ischemic preconditioning and exercise performance: shedding light through smallest worthwhile change | Marocolo M, Simim MA, Bernardino A, Monteiro IR, Patterson SD, da Mota GR. | 2019 | Not about recovery |
| Ischemic therapy in musculoskeletal medicine | Ramme AJ, Rourke BJ, Larson CM, Bedi A. | 2020 | Not about recovery |
| Isolated effects of caffeine and sodium bicarbonate ingestion on performance in the yo-yo test: a systematic review and meta-analysis | Grgic J, Garofolini A, Pickering C, Duncan MJ, Tinsley GM, Del Coso J. | 2020 | Not about recovery |
| Keto-adaptation and endurance exercise capacity, fatigue recovery, and exercise-induced muscle and organ damage prevention: a narrative review | Ma S, Suzuki K. | 2019 | Duplicate |
| Keto-adaptation and endurance exercise capacity, fatigue recovery, and exercise-induced muscle and organ damage prevention: a narrative review | Ma S, Suzuki K. | 2019 | Focused on physiology |
| L-arginine as a potential ergogenic aid in healthy subjects | Álvares TS, Meirelles CM, Bhambhani YN, Paschoalin VM, Gomes PS. | 2011 | Not about recovery |
| L-carnitine in the feeding of sports horses | Janicki B, Buzała M. | 2011 | Not about recovery |
| L-carnitine supplementation: influence upon physiological function | Kraemer WJ, Volek JS, Dunn-Lewis C. | 2008 | Focused on physiology |
| Laser‐assisted subepithelial keratectomy (lasek) versus laser‐assisted in‐situ keratomileusis (lasik) for correcting myopia | Kuryan J, Cheema A, Chuck RS. | 2017 | Focused on disease |
| Left ventricular function and cardiac biomarker release-the influence of exercise intensity, duration and mode: a systematic review and meta-analysis | Donaldson JA, Wiles JD, Coleman DA, Papadakis M, Sharma R, O’driscoll JM. | 2019 | Not about recovery |
| Legal nutritional supplements during a sporting event | Nicholas C. | 2008 | Duplicate |
| Legal nutritional supplements during a sporting event | Nicholas C. | 2008 | Not about recovery |
| Lessons learned from the united states ocean observatories initiative | Smith LM, Yarincik K, Vaccari L, Kaplan MB, Barth JA, Cram GS, Fram JP, Harrington M, Kawka OE, Kelley DS, Matthias P. | 2019 | Not about recovery |
| Limitations in intense exercise performance of athletes - effect of speed endurance training on ion handling and fatigue development | Hostrup M, Bangsbo J. | 2017 | Duplicate |
| Limitations in intense exercise performance of athletes - effect of speed endurance training on ion handling and fatigue development | Hostrup M, Bangsbo J. | 2017 | Not about recovery |
| Lipid metabolism and exercise | Lacour JR. | 2001 | Focused on physiology |
| Liver glycogen metabolism during and after prolonged endurance-type exercise | Gonzalez JT, Fuchs CJ, Betts JA, van Loon LJ. | 2016 | Duplicate |
| Liver glycogen metabolism during and after prolonged endurance-type exercise | Gonzalez JT, Fuchs CJ, Betts JA, van Loon LJ. | 2016 | Not about recovery |
| Long-term cognitive and functional impairments after critical illness | Rengel KF, Hayhurst CJ, Pandharipande PP, Hughes CG. | 2019 | Duplicate |
| Long-term cognitive and functional impairments after critical illness | Rengel KF, Hayhurst CJ, Pandharipande PP, Hughes CG. | 2019 | Not about recovery |
| Lower limbs wearable sports garments for muscle recovery: an umbrella review | Duarte JP, Fernandes RJ, Silva G, Sousa F, Machado L, Pereira JR, Vilas-Boas JP. | 2022 | Duplicate |
| Lower limbs wearable sports garments for muscle recovery: an umbrella review | Duarte JP, Fernandes RJ, Silva G, Sousa F, Machado L, Pereira JR, Vilas-Boas JP. | 2022 | Not a systematic review or meta-analysis |
| Lower mortality after early supervised pulmonary rehabilitation following copd-exacerbations: a systematic review and meta-analysis | Ryrsø CK, Godtfredsen NS, Kofod LM, Lavesen M, Mogensen L, Tobberup R, Farver-Vestergaard I, Callesen HE, Tendal B, Lange P, Iepsen UW. | 2018 | Focused on disease |
| Lymphocyte responses to maximal exercise - a physiological perspective | Nielsen HB. | 2003 | Focused on physiology |
| Macronutrients and performance | Williams C. | 1995 | Not about recovery |
| Magnesium and vitamin d supplementation on exercise performance | Hunt G, Sukumar D, Volpe SL. | 2021 | Not about recovery |
| Magnesium for skeletal muscle cramps | Garrison SR, Korownyk CS, Kolber MR, Allan GM, Musini VM, Sekhon RK, Dugré N. | 2020 | Not about recovery |
| Manipulating resistance training program variables to optimize maximum strength in men: a review | Tan B. | 1999 | Not about recovery, Not focused on endurance athletes |
| Manual therapy and exercise for rotator cuff disease | Page MJ, Green S, McBain B, Surace SJ, Deitch J, Lyttle N, Mrocki MA, Buchbinder R, Cochrane Musculoskeletal Group. | 2016 | Focused on disease |
| Massage and performance recovery: a meta-analytical review | Poppendieck W, Wegmann M, Ferrauti A, Kellmann M, Pfeiffer M, Meyer T. | 2016 | Duplicate |
| Mast‐cell stabilising agents to prevent exercise‐induced bronchoconstriction | Spooner C, Spooner GR, Rowe BH, Cochrane Airways Group. | 2003 | Focused on disease |
| Match analysis and player characteristics in rugby sevens | Ross A, Gill N, Cronin J. | 2014 | Not about recovery |
| Match analysis and the physiological demands of australian football | Gray AJ, Jenkins DG. | 2010 | Duplicate |
| Match analysis and the physiological demands of australian football | Gray AJ, Jenkins DG. | 2010 | Focused on physiology, Not focused on endurance athletes |
| Maximizing post-exercise anabolism: the case for relative protein intakes | Moore DR. | 2019 | Duplicate |
| Maximizing post-exercise anabolism: the case for relative protein intakes | Moore DR. | 2019 | Not focused on endurance athletes |
| May strenuous endurance sports activity damage the cardiovascular system of healthy athletes? a narrative review | Graziano F, Juhasz V, Brunetti G, Cipriani A, Szabo L, Merkely B, Corrado D, D'Ascenzi F, Vago H, Zorzi A. | 2022 | Duplicate |
| May strenuous endurance sports activity damage the cardiovascular system of healthy athletes? a narrative review | Graziano F, Juhasz V, Brunetti G, Cipriani A, Szabo L, Merkely B, Corrado D, D'Ascenzi F, Vago H, Zorzi A. | 2022 | Not a systematic review or meta-analysis |
| Mechanisms of exercise-induced mitochondrial biogenesis in skeletal muscle | Hood DA. | 2009 | Focused on physiology |
| Mechanistic and methodological perspectives on the impact of intense interval training on post-exercise metabolism | Moniz SC, Islam H, Hazell TJ. | 2020 | Not about recovery |
| Melatonin and agomelatine for preventing seasonal affective disorder | Nussbaumer‐Streit B, Greenblatt A, Kaminski‐Hartenthaler A, Van Noord MG, Forneris CA, Morgan LC, Gaynes BN, Wipplinger J, Lux LJ, Winkler D, Gartlehner G. | 2019 | Focused on disease |
| Metabolic clues regarding the enhanced performance of elite endurance athletes from orchiectomy-induced hormonal changes | Atwood CS, Bowen RL. | 2007 | Not about recovery |
| Metabolic markers in sports medicine | Banfi G, Colombini A, Lombardi G, Lubkowska A. | 2012 | Duplicate |
| Metabolic markers in sports medicine | Banfi G, Colombini A, Lombardi G, Lubkowska A. | 2012 | Focused on physiology |
| Metabolite concentration changes in humans after a bout of exercise: a systematic review of exercise metabolomics studies | Schranner D, Kastenmüller G, Schönfelder M, Römisch-Margl W, Wackerhage H. | 2020 | Not about recovery |
| Methodological approaches and related challenges associated with the determination of critical power and curvature constant | Muniz-Pumares D, Karsten B, Triska C, Glaister M. | 2019 | Not about recovery |
| Methods for monitoring training status and their effects on performance in rowing | Jurimae J. | 2008 | Not about recovery |
| Micronutrients and athletic performance: a review | Beck KL, von Hurst PR, O'Brien WJ, Badenhorst CE. | 2021 | Not about recovery |
| Micronutrients and exercise: anti-oxidants and minerals | Clarkson PM. | 1995 | Focused on physiology |
| Microrna profile and adaptive response to exercise training: a review | Domańska-Senderowska D, Laguette MN, Jegier A, Cięszczyk P, September AV, Brzeziańska-Lasota E. | 2019 | Duplicate |
| Microrna profile and adaptive response to exercise training: a review | Domańska-Senderowska D, Laguette MN, Jegier A, Cięszczyk P, September AV, Brzeziańska-Lasota E. | 2019 | Not about recovery |
| Military applicability of interval training for health and performance | Gibala MJ, Gagnon PJ, Nindl BC. | 2015 | Not about recovery, Not focused on endurance athletes |
| Military-specific application of nutritional supplements: a brief overview | Hoedebecke K, Brink W. | 2015 | Not focused on endurance athletes |
| Milk: the new sports drink? a review | Roy BD. | 2008 | Not a systematic review or meta-analysis |
| Minimally invasive ways to monitor changes in cardiocirculatory fitness in running-based sports: a systematic review | Schimpchen J, Correia PF, Meyer T. | 2022 | Focused on physiology |
| Mobility after stroke: relearning to walk | Stephan KM, Pérennou D. | 2021 | Focused on disease |
| Modifications of arterial baroreflexes: obligatory roles in cardiovascular regulation in stress and poststress recovery | Nosaka S. | 1996 | Duplicate |
| Modifications of arterial baroreflexes: obligatory roles in cardiovascular regulation in stress and poststress recovery | Nosaka S. | 1996 | Focused on physiology |
| Molecular responses to high-intensity interval exercise | Gibala M. | 2009 | Not about recovery, Focused on physiology |
| Monitoring athletic training status through autonomic heart rate regulation: a systematic review and meta-analysis | Bellenger CR, Fuller JT, Thomson RL, Davison K, Robertson EY, Buckley JD. | 2016 | Duplicate |
| Monitoring athletic training status through autonomic heart rate regulation: a systematic review and meta-analysis | Bellenger CR, Fuller JT, Thomson RL, Davison K, Robertson EY, Buckley JD. | 2016 | Focused on physiology |
| Monitoring of performance and training in rowing | Mäestu J, Jürimäe J, Jürimäe T. | 2005 | Duplicate |
| Monitoring of performance and training in rowing | Mäestu J, Jürimäe J, Jürimäe T. | 2005 | Not about recovery |
| Monitoring stress and allostatic load in first responders and tactical operators using heart rate variability: a systematic review | Corrigan SL, Roberts S, Warmington S, Drain J, Main LC. | 2021 | Not about recovery |
| Motor imagery for gait rehabilitation after stroke | Silva S, Borges LR, Santiago L, Lucena L, Lindquist AR, Ribeiro T. | 2020 | Duplicate |
| Motor imagery for gait rehabilitation after stroke | Silva S, Borges LR, Santiago L, Lucena L, Lindquist AR, Ribeiro T. | 2020 | Focused on disease |
| Motor rehabilitation in stroke and traumatic brain injury: stimulating and intense | Breceda EY, Dromerick AW. | 2013 | Duplicate |
| Motor rehabilitation in stroke and traumatic brain injury: stimulating and intense | Breceda EY, Dromerick AW. | 2013 | Focused on disease |
| Mucosal immunity and respiratory illness in elite athletes | Gleeson M. | 2000 | Focused on disease |
| Multi-task exposure assessment to infer musculoskeletal disorder risk: a scoping review of injury causation theories and tools available to assess exposures | Veerasammy S, Davidson JB, Fischer SL. | 2022 | Not about recovery, Not a systematic review or meta-analysis |
| Multiple session early psychological interventions for the prevention of post‐traumatic stress disorder | Roberts NP, Kitchiner NJ, Kenardy J, Robertson L, Lewis C, Bisson JI. | 2019 | Not about recovery |
| Multiple sprint work: physiological responses, mechanisms of fatigue and the influence of aerobic fitness | Glaister M. | 2005 | Not about recovery |
| Muscle atrophy and procedures for training after spinal cord injury | Gordon T, Mao J. | 1994 | Duplicate |
| Muscle atrophy and procedures for training after spinal-cord injury | Gordon T, Mao J. | 1994 | Not about recovery |
| Muscle damage and inflammation during recovery from exercise | Peake JM, Neubauer O, Della Gatta PA, Nosaka K. | 2017 | Focused on physiology |
| Muscle protein turnover in endurance training: a review | Seene T, Kaasik P, Alev K. | 2011 | Duplicate |
| Muscle protein turnover in endurance training: a review | Seene T, Kaasik P, Alev K. | 2011 | Not about recovery |
| Muscle recruitment pattern in cycling: a review | So RC, Ng JK, Ng GY. | 2005 | Not about recovery |
| Muscle strain injuries | Garrett WE Jr. | 1996 | Not about recovery |
| Muscular fatigue | Sesboüé B, Guincestre JY. | 2006 | Not about recovery |
| Myocarditis in the athlete: arrhythmogenic substrates, clinical manifestations, management, and eligibility decisions | Vio R, Zorzi A, Corrado D. | 2020 | Not about recovery |
| Myofibrillar and mitochondrial protein synthesis rates do not differ in young men following the ingestion of carbohydrate with milk protein, whey, or micellar casein after concurrent resistance- and endurance-type exercise | Churchward-Venne TA, Pinckaers PJ, Smeets JS, Peeters WM, Zorenc AH, Schierbeek H, Rollo I, Verdijk LB, van Loon LJ. | 2019 | Duplicate |
| Myofibrillar and mitochondrial protein synthesis rates do not differ in young men following the ingestion of carbohydrate with whey, soy, or leucine-enriched soy protein after concurrent resistance- and endurance-type exercise | Churchward-Venne TA, Pinckaers PJ, Smeets JS, Peeters WM, Zorenc AH, Schierbeek H, Rollo I, Verdijk LB, van Loon LJ. | 2019 | Not a systematic review or meta-analysis |
| Nedocromil sodium for preventing exercise‐induced bronchoconstriction | Spooner C, Saunders LD, Rowe BH, Cochrane Airways Group. | 2002 | Focused on disease |
| Neuralgic amyotrophy | IJspeert J, Janssen RMJ, van Alfen N. | 2021 | Duplicate |
| Neuralgic amyotrophy | IJspeert J, Janssen RMJ, van Alfen N. | 2021 | Focused on disease |
| Neuromuscular control in lumbar disorders | Leinonen V. | 2004 | Focused on disease |
| Neuromuscular electrical stimulation (nmes) for patellofemoral pain syndrome | Martimbianco AL, Torloni MR, Andriolo BN, Porfírio GJ, Riera R. | 2017 | Focused on disease |
| Neuromuscular factors associated with decline in long-distance running performance in master athletes | Brisswalter J, Nosaka K. | 2013 | Duplicate |
| Neuromuscular factors associated with decline in long-distance running performance in master athletes | Brisswalter J, Nosaka K. | 2013 | Not about recovery |
| Neuromuscular responses to fatiguing locomotor exercise | Brownstein CG, Millet GY, Thomas K. | 2021 | Focused on physiology |
| Neuromuscular treatments for speech and swallowing: a tutorial | Clark HM. | 2003 | Duplicate |
| Neuromuscular treatments for speech and swallowing: a tutorial | Clark HM. | 2003 | Not about recovery |
| New generation antidepressants for depression in children and adolescents: a network meta‐analysis | Hetrick SE, McKenzie JE, Bailey AP, Sharma V, Moller CI, Badcock PB, Cox GR, Merry SN, Meader N. | 2021 | Not about recovery |
| Non-carbohydrate dietary factors and their influence on post-exercise glycogen storage: a review | Lawler TP, Cialdella-Kam L. | 2020 | Duplicate |
| Non-carbohydrate dietary factors and their influence on post-exercise glycogen storage: a review | Lawler TP, Cialdella-Kam L. | 2020 | Focused on physiology |
| Non-pharmacological management of persistent headaches associated with neck pain: a clinical practice guideline from the ontario protocol for traffic injury management (optima) collaboration | Côté P, Yu H, Shearer HM, Randhawa K, Wong JJ, Mior S, Ameis A, Carroll LJ, Nordin M, Varatharajan S, Sutton D. | 2019 | Not about recovery |
| Noninvasive neuromodulation in poststroke gait disorders: rationale, feasibility, and state of the art | Chieffo R, Comi G, Leocani L. | 2016 | Duplicate |
| Noninvasive neuromodulation in poststroke gait disorders: rationale, feasibility, and state of the art | Chieffo R, Comi G, Leocani L. | 2016 | Focused on disease |
| Nonoperative and postoperative rehabilitation for glenohumeral instability | Wilk KE, Macrina LC. | 2013 | Not about recovery |
| Normative yo-yo intermittent recovery level 1 and yo-yo intermittent endurance level 1 test values of boys aged 9-16 years | Schmitz B, Pfeifer C, Kreitz K, Borowski M, Faldum A, Brand SM. | 2019 | Not about recovery |
| Normative yo-yo intermittent recovery level 1 and yo-yo intermittent endurance level 1 test values of boys aged 9-16years | Schmitz B, Pfeifer C, Kreitz K, Borowski M, Faldum A, Brand SM. | 2019 | Duplicate |
| Nutrient timing: a garage door of opportunity? | Arent SM, Cintineo HP, McFadden BA, Chandler AJ, Arent MA. | 2020 | Not a systematic review or meta-analysis |
| Nutrition and athletic performance | Rodriguez NR, Di Marco NM, Langley S. American College of Sports Medicine position stand. | 2009 | Not a systematic review or meta-analysis |
| Nutrition and health--nutrition and performance in sports | Saris WH, van Loon LJ. | 2004 | Not a systematic review or meta-analysis |
| Nutrition and supplement update for the endurance athlete: review and recommendations | Vitale K, Getzin A. | 2019 | Duplicate |
| Nutrition and supplement update for the endurance athlete: review and recommendations | Vitale K, Getzin A. | 2019 | Not a systematic review or meta-analysis |
| Nutrition for distance events | Burke LM, Millet G, Tarnopolsky MA and International Association of Athletics Federations. | 2007 | Not a systematic review or meta-analysis |
| Nutrition for optimal performance during exercise: carbohydrate and fat | Brown RC. | 2002 | Not about recovery |
| Nutrition in the sport practice: adaptation of the food guide pyramid to the characteristics of athletes diet | González-Gross M, Gutiérrez A, Mesa JL, Ruiz-Ruiz J, Castillo MJ. | 2001 | Not a systematic review or meta-analysis |
| Nutrition to support recovery from endurance exercise: optimal carbohydrate and protein replacement | Moore DR. | 2015 | Not a systematic review or meta-analysis |
| Nutritional aspects of women strength athletes | Volek JS, Forsythe CE, Kraemer WJ. | 2006 | Duplicate |
| Nutritional aspects of women strength athletes | Volek JS, Forsythe CE, Kraemer WJ. | 2006 | Not focused on endurance athletes |
| Nutritional concerns in the diabetic athlete | Jensen J. | 2004 | Focused on disease |
| Nutritional consideration in the aging athlete | Tarnopolsky MA. | 2008 | Duplicate |
| Nutritional consideration in the aging athlete | Tarnopolsky MA. | 2008 | Not focused on endurance athletes |
| Nutritional implications for ultra-endurance walking and running events | Williamson E. | 2016 | Not a systematic review or meta-analysis |
| Nutritional needs for exercise in the heat | Burke LM. | 2001 | Not about recovery |
| Nutritional optimization for female elite football players-topical review | de Sousa MV, Lundsgaard AM, Christensen PM, Christensen L, Randers MB, Mohr M, Nybo L, Kiens B, Fritzen AM. | 2022 | Not focused on endurance athletes |
| Nutritional practices of athletes: are they sub-optimal? | Hawley JA, Dennis SC, Lindsay FH, Noakes TD. | 1995 | Not a systematic review or meta-analysis |
| Nutritional practices of male and female endurance cyclists | Burke LM. | 2001 | Duplicate |
| Nutritional practices of male and female endurance cyclists | Burke LM. | 2001 | Not a systematic review or meta-analysis |
| Nutritional recommendations for synchronized swimming | Robertson S, Benardot D, Mountjoy M. | 2014 | Not focused on endurance athletes |
| Nutritional recommendations for water polo | Cox GR, Mujika I, van den Hoogenband CR. | 2014 | Not focused on endurance athletes |
| Nutritional strategies to influence adaptations to training | Spriet LL, Gibala MJ. | 2004 | Not about recovery, Focused on physiology |
| Nutritional strategies to optimize training and racing in middle-distance athletes | Stellingwerff T, Boit MK, Res PT and International Association of Athletics Federations. | 2007 | Not about recovery |
| Nutritional strategies to promote postexercise recovery | Beelen M, Burke LM, Gibala MJ, van Loon L JC. | 2010 | Duplicate |
| Nutritional strategies to promote postexercise recovery | Beelen M, Burke LM, Gibala MJ, van Loon L JC. | 2010 | Not a systematic review or meta-analysis |
| Omega-3 fatty acids for sport performance-are they equally beneficial for athletes and amateurs? a narrative review | Thielecke F, Blannin A. | 2020 | Duplicate |
| Omega-3 fatty acids for sport performance-are they equally beneficial for athletes and amateurs? a narrative review | Thielecke F, Blannin A. | 2020 | Not about recovery, Not a systematic review or meta-analysis |
| Omega‐3 fatty acids for depression in adults | Appleton KM, Voyias PD, Sallis HM, Dawson S, Ness AR, Churchill R, Perry R. | 2021 | Not about recovery |
| On-ice return-to-hockey progression after anterior cruciate ligament reconstruction | Capin JJ, Behrns W, Thatcher K, Arundale A, Smith AH, Snyder-Mackler L. | 2017 | Not about recovery |
| Optimising training adaptations and performance in military environment | Kyröläinen H, Pihlainen K, Vaara JP, Ojanen T, Santtila M. | 2018 | Not about recovery, Not focused on endurance athletes |
| Optimization of exercise countermeasures for human space flight: operational considerations for concurrent strength and aerobic training | Jones TW, Petersen N, Howatson G. | 2019 | Not about recovery |
| Optimizing recovery to support multi-evening cycling competition performance | Richard NA, Koehle MS. | 2019 | Not a systematic review or meta-analysis |
| Oral branched-chain amino acids supplementation in athletes: a systematic review | Martinho DV, Nobari H, Faria A, Field A, Duarte D, Sarmento H. | 2022 | Duplicate |
| Oral branched-chain amino acids supplementation in athletes: a systematic review | Martinho DV, Nobari H, Faria A, Field A, Duarte D, Sarmento H. | 2022 | Not eligible |
| Oral motor disorders in humans | Clark GT, Koyano K, Browne PA. | 1993 | Focused on disease |
| Outcome after surgical repair of proximal hamstring avulsions: a systematic review | van der Made AD, Reurink G, Gouttebarge V, Tol JL, Kerkhoffs GM. | 2015 | Focused on disease |
| Outcomes after operative and nonoperative treatment of proximal hamstring avulsions: a systematic review and meta-analysis | Bodendorfer BM, Curley AJ, Kotler JA, Ryan JM, Jejurikar NS, Kumar A, Postma WF. | 2018 | Focused on disease |
| Overactivity in chronic pain, the role of pain-related endurance and neuromuscular activity an interdisciplinary, narrative review | Hasenbring MI, Andrews NE, Ebenbichler G. | 2020 | Not a systematic review or meta-analysis |
| Overactivity in chronic pain, the role of pain-related endurance and neuromuscular activity: an interdisciplinary, narrative review | Hasenbring MI, Andrews NE, Ebenbichler G. | 2020 | Duplicate |
| Overtraining in endurance athletes - a brief review | Lehmann M, Foster C, Keul J. | 1993 | Not about recovery |
| Overtraining in endurance athletes: a brief review | Lehmann M, Foster C, Keul J. | 1993 | Duplicate |
| Passclaim - physical performance and fitness | Saris WH, Antoine JM, Brouns F, Fogelholm M, Gleeson M, Hespel P, Jeukendrup AE, Maughan RJ, Pannemans D, Stich V. | 2003 | Duplicate |
| Passclaim - physical performance and fitness | Saris WH, Antoine JM, Brouns F, Fogelholm M, Gleeson M, Hespel P, Jeukendrup AE, Maughan RJ, Pannemans D, Stich V. | 2003 | Not about recovery |
| Patellar tendon versus hamstring tendon autograft for anterior cruciate ligament rupture in adults | Mohtadi NG, Chan DS, Dainty KN, Whelan DB. | 2011 | Not about recovery |
| Pathogenesis of idiopathic inflammatory myopathies | Grundtman C, Lundberg IE. | 2007 | Not about recovery |
| Pathophysiology of noncardiac syncope in athletes | Christou GA, Christou KA, Kiortsis DN. | 2018 | Not about recovery |
| Per-cooling (using cooling systems during physical exercise) enhances physical and cognitive performances in hot environments. a narrative review | Douzi W, Dupuy O, Theurot D, Smolander J, Dugué B. | 2020 | Duplicate |
| Per-cooling (using cooling systems during physical exercise) enhances physical and cognitive performances in hot environments. a narrative review | Douzi W, Dupuy O, Theurot D, Smolander J, Dugué B. | 2020 | Not a systematic review or meta-analysis |
| Performance adaptations to intensified training in top-level football | Hostrup M, Bangsbo J. | 2022 | Duplicate |
| Performance adaptations to intensified training in top-level football | Hostrup M, Bangsbo J. | 2022 | Not about recovery |
| Performance analysis of surfing: a review | Farley OR, Abbiss CR, Sheppard JM. | 2017 | Duplicate |
| Performance analysis of surfing: a review | Farley OR, Abbiss CR, Sheppard JM. | 2017 | Not about recovery |
| Performance effects of periodized carbohydrate restriction in endurance trained athletes - a systematic review and meta-analysis | Gejl KD, Nybo L. | 2021 | Duplicate |
| Performance effects of periodized carbohydrate restriction in endurance trained athletes - a systematic review and meta-analysis | Gejl KD, Nybo L. | 2021 | Not eligible |
| Performance enhancing hormone doping in sport | Handelsman DJ. | 2020 | Not about recovery |
| Pharmacological interventions for the treatment of disordered and problem gambling | Lubman D, Thomas S, Bowden-Jones H, Cowlishaw S. | 2022 | Not about recovery |
| Pharmacotherapy for patellofemoral pain syndrome | Heintjes EM, Berger M, Bierma‐Zeinstra SM, Bernsen RM, Verhaar JA, Koes BW. | 2004 | Focused on disease |
| Physical activity and bone health | Kohrt WM, Bloomfield SA, Little KD, Nelson ME, Yingling VR. | 2004 | Focused on physiology |
| Physical activity in women with breast cancer and those after mastectomy | Gierszon P, Stachura A, Paziewska M, Samardakiewicz M, Furtak-Niczyporuk M. | 2020 | Focused on disease |
| Physical and physiological attributes of wrestlers: an update | Chaabene H, Negra Y, Bouguezzi R, Mkaouer B, Franchini E, Julio U, Hachana Y. | 2017 | Duplicate |
| Physical and physiological attributes of wrestlers: an update | Chaabene H, Negra Y, Bouguezzi R, Mkaouer B, Franchini E, Julio U, Hachana Y. | 2017 | Focused on physiology |
| Physical and physiological demands of futsal | Naser N, Ali A, Macadam P. | 2017 | Not about recovery, Not focused on endurance athletes |
| Physical and physiological profiles of taekwondo athletes | Bridge CA, Ferreira da Silva Santos J, Chaabène H, Pieter W, Franchini E. | 2014 | Duplicate |
| Physical and physiological profiles of taekwondo athletes | Bridge CA, Ferreira da Silva Santos J, Chaabène H, Pieter W, Franchini E. | 2014 | Not about recovery |
| Physical determinants, emerging concepts, and training approaches in gait of individuals with spinal cord injury | Barbeau H, Nadeau S, Garneau C. | 2006 | Focused on disease, Not about recovery |
| Physical exercise ameliorates deficits induced by traumatic brain injury | Archer T, Svensson K, Alricsson M. | 2012 | Focused on disease |
| Physical exercise and dna injury: good or evil? | Danese E, Lippi G, Sanchis-Gomar F, Brocco G, Rizzo M, Banach M, Montagnana M. | 2017 | Not about recovery |
| Physical exercise training interventions for children and young adults during and after treatment for childhood cancer | Braam KI, Takken T, Veening MA, van Dulmen‐den Broeder E, Kaspers GJ. | 2016 | Focused on disease |
| Physical exercise: a novel tool to protect mitochondrial health | Sorriento D, Di Vaia E, Iaccarino G. | 2021 | Not about recovery |
| Physical function and physical activity in obese adults after total knee arthroplasty | Smith WA, Zucker-Levin A, Mihalko WM, Williams M, Loftin M, Gurney JG. | 2017 | Not about recovery |
| Physical rehabilitation and occupational therapy | Capozzi LC, Dolgoy ND, McNeely ML. | 2018 | Not about recovery |
| Physical tests for shoulder impingements and local lesions of bursa, tendon or labrum that may accompany impingement | Hanchard NC, Lenza M, Handoll HH, Takwoingi Y. | 2013 | Focused on disease |
| Physical therapy in unilateral and bilateral vestibular hypofunction | Benito-Orejas JI, Aylagas-Andrés MJ, Martín-Mooratinos C, Gallardo-Chaparro I, Pérez-Hickman L, Aladro-Abad V, Plaza-García N, López-Franco JM, Díez-Rabadán B, García-Franco C, Seco-Rodríguez L. | 2020 | Not about recovery |
| Physiological adaptations to interval training and the role of exercise intensity | MacInnis MJ, Gibala MJ. | 2017 | Duplicate |
| Physiological adaptations to interval training and the role of exercise intensity | MacInnis MJ, Gibala MJ. | 2017 | Focused on physiology |
| Physiological adaptations to interval training to promote endurance | Gibala MJ, Bostad W, McCarthy DG. | 2019 | Not about recovery |
| Physiological and nutritional aspects of post-exercise recovery specific recommendations for female athletes | Hausswirth C, Le Meur Y. | 2011 | Not a systematic review or meta-analysis |
| Physiological and pathophysiological features of the control of lipolysis and lipid mobilization by natriuretic peptides | Moro C, Berlan M, Lafontan M. | 2006 | Focused on physiology |
| Physiological and performance adaptations to high-intensity interval training | Gibala MJ, Jones AM. | 2013 | Not about recovery |
| Physiological and tactical on-court demands of water polo | Botonis PG, Toubekis AG, Platanou TI. | 2019 | Duplicate |
| Physiological and tactical on-court demands of water polo | Botonis PG, Toubekis AG, Platanou TI. | 2019 | Not about recovery |
| Physiological aspects of competitive cross-country skiing | Hoffman MD, Clifford PS. | 1992 | Focused on physiology |
| Physiological aspects of soccer refereeing performance and training | Castagna C, Abt G, D’Ottavio S. | 2007 | Not about recovery |
| Physiological basis of brief vigorous exercise to improve health | Gibala MJ, Little JP. | 2020 | Focused on physiology |
| Physiological basis of brief, intense interval training to enhance maximal oxygen uptake: a mini-review | Gibala MJ, MacInnis MJ. | 2022 | Focused on physiology, Not a systematic review or meta-analysis |
| Physiological basis of temperature-dependent biogeography: trade-offs in muscle design and performance in polar ectotherms | Portner HO. | 2002 | Focused on physiology |
| Physiological benefits and performance of sea water ingestion for athletes in endurance events: a systematic review | Aragón-Vela J, González-Acevedo O, Plaza-Diaz J, Casuso RA, Huertas JR. | 2022 | Duplicate |
| Physiological benefits and performance of sea water ingestion for athletes in endurance events: a systematic review | Aragón-Vela J, González-Acevedo O, Plaza-Diaz J, Casuso RA, Huertas JR. | 2022 | Not about recovery |
| Physiological responses to low-volume interval training in women | Skelly LE, Bailleul C, Gillen JB. | 2021 | Focused on physiology |
| Physiological responses to repeated running sprint ability tests: a systematic review | Charron J, Garcia JEV, Roy P, Ferland PM, Comtois AS. | 2020 | Not about recovery, Focused on physiology |
| Physiology of difficult rock climbing | Watts PB. | 2004 | Duplicate |
| Physiology of difficult rock climbing | Watts PB. | 2004 | Focused on physiology |
| Physiology of ice hockey | Montgomery DL. | 1988 | Focused on physiology, Not focused on endurance athletes |
| Physiotherapy in patients with chronic pain | Konrad B, Prate K, Brannasch K, Ettrich U. | 2010 | Focused on disease |
| Physiotherapy interventions for shoulder pain | Green S, Buchbinder R, Hetrick SE, Cochrane Musculoskeletal Group. | 2003 | Focused on disease |
| Plant adaptogens-history and future perspectives | Todorova V, Ivanov K, Delattre C, Nalbantova V, Karcheva-Bahchevanska D, Ivanova S. | 2021 | Duplicate |
| Plant adaptogens-history and future perspectives | Todorova V, Ivanov K, Delattre C, Nalbantova V, Karcheva-Bahchevanska D, Ivanova S. | 2021 | Not about recovery |
| Platelet-rich plasma injection for adults with acute achilles tendon rupture: the path-2 rct | Alsousou J, Keene D, Harrison P, Hulley P, Wagland S, Thompson J, Byrne C, Schlüssel M, O'Connor H, Dutton S, Lamb S. | 2019 | Focused on disease |
| Platelet‐rich therapies for musculoskeletal soft tissue injuries | Moraes VY, Lenza M, Tamaoki MJ, Faloppa F, Belloti JC. | 2014 | Focused on disease |
| Position of the american dietetic association, dietitians of canada, and the american college of sports medicine: nutrition and athletic performance | Rodriguez NR, DiMarco NM, Langley S. | 2009 | Not a systematic review or meta-analysis |
| Position stand on androgen and human growth hormone use | Hoffman JR, Kraemer WJ, Bhasin S, Storer T, Ratamess NA, Haff GG, Willoughby DS, Rogol AD. | 2009 | Not about recovery |
| Post-exercise cold water immersion effects on physiological adaptations to resistance training and the underlying mechanisms in skeletal muscle: a narrative review | Petersen AC, Fyfe JJ. | 2021 | Duplicate |
| Post-exercise cold water immersion effects on physiological adaptations to resistance training and the underlying mechanisms in skeletal muscle: a narrative review | Petersen AC, Fyfe JJ. | 2021 | Not a systematic review or meta-analysis |
| Post-exercise ingestion of carbohydrate, protein and water: a systematic review and meta-analysis for effects on subsequent athletic performance | McCartney D, Desbrow B, Irwin C. | 2018 | Duplicate |
| Post-exercise nutrition in football - a performance benefit? | Niess AM, Striegel H. | 2013 | Not about recovery |
| Post-exercise recovery for the endurance athlete with type 1 diabetes: a consensus statement | Scott SN, Fontana FY, Cocks M, Morton JP, Jeukendrup A, Dragulin R, Wojtaszewski JF, Jensen J, Castol R, Riddell MC, Stettler C. | 2021 | Duplicate |
| Post-exercise recovery for the endurance athlete with type 1 diabetes: a consensus statement | Scott SN, Fontana FY, Cocks M, Morton JP, Jeukendrup A, Dragulin R, Wojtaszewski JF, Jensen J, Castol R, Riddell MC, Stettler C. | 2021 | Focused on disease |
| Postexercise dietary protein strategies to maximize skeletal muscle repair and remodeling in masters endurance athletes: a review | Doering TM, Reaburn PR, Phillips SM, Jenkins DG. | 2016 | Duplicate |
| Postexercise dietary protein strategies to maximize skeletal muscle repair and remodeling in masters endurance athletes: a review | Doering TM, Reaburn PR, Phillips SM, Jenkins DG. | 2016 | Not a systematic review or meta-analysis |
| Postexercise hypotension and sustained postexercise vasodilatation: what happens after we exercise? | Halliwill JR, Buck TM, Lacewell AN, Romero SA. | 2013 | Not about recovery |
| Postexercise muscle glycogen resynthesis in humans | Burke LM, van Loon LJ, Hawley JA. | 2017 | Focused on physiology |
| Postexercise recovery period: carbohydrate and protein metabolism | Viru A. | 1996 | Duplicate |
| Postexercise recovery period: carbohydrate and protein metabolism | Viru A. | 1996 | Not about recovery |
| Potential of bioremediation and pgp traits in streptomyces as strategies for bio-reclamation of salt-affected soils for agriculture | Romano-Armada N, Yañez-Yazlle MF, Irazusta VP, Rajal VB, Moraga NB. | 2020 | Duplicate |
| Potential of bioremediation and pgp traits in streptomyces as strategies for bio-reclamation of salt-affected soils for agriculture | Romano-Armada N, Yañez-Yazlle MF, Irazusta VP, Rajal VB, Moraga NB. | 2020 | Not about recovery |
| Potential relevance of bioactive peptides in sports nutrition | König D, Kohl J, Jerger S, Centner C. | 2021 | Duplicate |
| Potential relevance of bioactive peptides in sports nutrition | König D, Kohl J, Jerger S, Centner C. | 2021 | Not about recovery |
| Power assessment in road cycling: a narrative review | Sitko S, Cirer-Sastre R, Corbi F, López-Laval I. | 2020 | Not a systematic review or meta-analysis |
| Power profiling and the power-duration relationship in cycling: a narrative review | Leo P, Spragg J, Podlogar T, Lawley JS, Mujika I. | 2021 | Not about recovery, Not a systematic review or meta-analysis |
| Practices and applications of heart rate variability monitoring in endurance athletes | Lundstrom CJ, Foreman NA, Biltz G. | 2022 | Focused on physiology |
| Pre-exercise nutrition: the role of macronutrients, modified starches and supplements on metabolism and endurance performance | Ormsbee MJ, Bach CW, Baur DA. | 2014 | Not about recovery |
| Prehabilitation, from theory to clinical practice: implementation and perspectives | Le Guen M, Barizien N, Bizard A, Fischler M, Carli F. | 2019 | Not about recovery |
| Prehabilitation. preparing patients for surgery to improve functional recovery and reduce postoperative morbidity | Debes C, Aissou M, Beaussier M. | 2014 | Focused on disease |
| Present‐centered therapy (pct) for post‐traumatic stress disorder (ptsd) in adults | Belsher BE, Beech E, Evatt D, Smolenski DJ, Shea MT, Otto JL, Rosen CS, Schnurr PP. | 2019 | Focused on disease |
| Principles and applications of therapeutic exercises for small animals | Drum MG, Marcellin-Little DJ, Davis MS. | 2015 | Not about recovery |
| Pro-arrhythmic effects of low plasma [k(+)] in human ventricle: an illustrated review | Trenor B, Cardona K, Romero L, Gomez JF, Saiz J, Rajamani S, Belardinelli L, Giles W. | 2018 | Duplicate |
| Pro-arrhythmic effects of low plasma [k+] in human ventricle: an illustrated review | Trenor B, Cardona K, Romero L, Gomez JF, Saiz J, Rajamani S, Belardinelli L, Giles W. | 2018 | Not about recovery, Not a systematic review or meta-analysis |
| Probiotics and athletic performance: a systematic review | Nichols AW. | 2017 | Not about recovery |
| Probiotics and sports: a new magic bullet? | Leite GSF, Resende Master Student AS, West NP, Lancha AH Jr. | 2019 | Duplicate |
| Probiotics and sports: a new magic bullet? | Leite GSF, Resende Master Student AS, West NP, Lancha AH Jr. | 2019 | Not a systematic review or meta-analysis |
| Programming plyometric-jump training in soccer: a review | Ramirez-Campillo R, Moran J, Oliver JL, Pedley JS, Lloyd RS, Granacher U. | 2022 | Not about recovery |
| Promoting training adaptations through nutritional interventions | Hawley JA, Tipton KD, Millard-Stafford ML. | 2006 | Not about recovery |
| Prospective function of different antioxidant containing natural products in the treatment of neurodegenerative diseases | Rahman MH, Akter R, Kamal MA. | 2021 | Duplicate |
| Prospective function of different antioxidant containing natural products in the treatment of neurodegenerative diseases | Rahman MH, Akter R, Kamal MA. | 2021 | Focused on disease |
| Protein degradation during endurance exercise and recovery | Dohm GL, Tapscott EB, Kasperek GJ. | 1987 | Focused on physiology |
| Protein for exercise and recovery | Kreider RB, Campbell B. | 2009 | Not a systematic review or meta-analysis |
| Protein requirements for master athletes: just older versions of their younger selves | Moore DR. | 2021 | Duplicate |
| Protein requirements for master athletes: just older versions of their younger selves | Moore DR. | 2021 | Not a systematic review or meta-analysis |
| Protein turnover, amino acid requirements and recommendations for athletes and active populations | Poortmans JR, Carpentier A, Pereira-Lancha LO, Lancha A Jr. | 2012 | Duplicate |
| Protein turnover, amino acid requirements and recommendations for athletes and active populations | Poortmans JR, Carpentier A, Pereira-Lancha LO, Lancha A Jr. | 2012 | Not about recovery |
| Protein-based supplementation to enhance recovery in team sports: what is the evidence? | Poulios A, Georgakouli K, Draganidis D, Deli CK, Tsimeas PD, Chatzinikolaou A, Papanikolaou K, Batrakoulis A, Mohr M, Jamurtas AZ, Fatouros IG. | 2019 | Not focused on endurance athletes |
| Psychiatric, psychosocial, and rehabilitative aspects of lung transplantation | Craven JL, Bright J, Dear CL. | 1990 | Focused on disease |
| Psychological first aid and nursing | Kılıç N, Şimşek N. | 2018 | Not about recovery |
| Psychological interventions to foster resilience in healthcare professionals | Kunzler AM, Helmreich I, Chmitorz A, König J, Binder H, Wessa M, Lieb K. | 2020 | Not about recovery |
| Psychological therapies for anxiety and depression in children and adolescents with long‐term physical conditions | Thabrew H, Stasiak K, Hetrick SE, Donkin L, Huss JH, Highlander A, Wong S, Merry SN. | 2018 | Not about recovery |
| Psychological therapies for children and adolescents exposed to trauma | Gillies D, Maiocchi L, Bhandari AP, Taylor F, Gray C, O'Brien L. | 2016 | Focused on disease |
| Psychological therapies for women who experience intimate partner violence | Hameed M, O'Doherty L, Gilchrist G, Tirado-Muñoz J, Taft A, Chondros P, Feder G, Tan M, Hegarty K. | 2020 | Not about recovery |
| Pulmonary and respiratory muscle function in response to marathon and ultra-marathon running: a review | Tiller NB. | 2019 | Duplicate |
| Pulmonary and respiratory muscle function in response to marathon and ultra-marathon running: a review | Tiller NB. | 2019 | Not about recovery |
| Pulmonary rehabilitation in patients undergoing lung-volume reduction surgery | Bartels MN, Kim H, Whiteson JH, Alba AS. | 2006 | Not about recovery |
| Putative effects of doping in cycling | Kuipers H. | 2006 | Not about recovery |
| Quantification of training and competition loads in endurance sports: methods and applications | Mujika I. | 2017 | Duplicate |
| Quantification of training and competition loads in endurance sports: methods and applications | Mujika I. | 2017 | Not about recovery |
| Reactive oxygen/nitrogen species and contractile function in skeletal muscle during fatigue and recovery | Cheng AJ, Yamada T, Rassier DE, Andersson DC, Westerblad H, Lanner JT. | 2016 | Duplicate |
| Reactive oxygen/nitrogen species and contractile function in skeletal muscle during fatigue and recovery | Cheng AJ, Yamada T, Rassier DE, Andersson DC, Westerblad H, Lanner JT. | 2016 | Focused on physiology |
| Recent advances in specific training for cycling | Faria EW. | 2009 | Not about recovery |
| Recent progress in applicability of exercise immunology and inflammation research to sports nutrition | Suzuki K. | 2021 | Not a systematic review or meta-analysis |
| Recommendations for treatment of hyponatraemia at endurance events | Hsieh M. | 2004 | Duplicate |
| Recommendations for treatment of hyponatraemia at endurance events | Hsieh M. | 2004 | Focused on disease |
| Recovery after exercise: what is the current state of play? | Peake JM. | 2019 | Not a systematic review or meta-analysis |
| Recovery from training: a brief review: brief review | Bishop PA, Jones E, Woods AK. | 2008 | Not a systematic review or meta-analysis |
| Recovery nutrition: timing and composition after endurance exercise | Millard-Stafford M, Childers WL, Conger SA, Kampfer AJ, Rahnert JA. | 2008 | Duplicate |
| Recovery nutrition: timing and composition after endurance exercise | Millard-Stafford M, Childers WL, Conger SA, Kampfer AJ, Rahnert JA. | 2008 | Not a systematic review or meta-analysis |
| Recovery of central and peripheral neuromuscular fatigue after exercise | Carroll TJ, Taylor JL, Gandevia SC. | 2017 | Duplicate |
| Recovery of central and peripheral neuromuscular fatigue after exercise | Carroll TJ, Taylor JL, Gandevia SC. | 2017 | Focused on physiology |
| Recovery of heart rate following intense dynamic exercise | Coote JH. | 2010 | Duplicate |
| Recovery of heart rate following intense dynamic exercise | Coote JH. | 2010 | Focused on physiology |
| Recovery of the immune system after exercise | Peake JM, Neubauer O, Walsh NP, Simpson RJ. | 2017 | Focused on physiology |
| Redox mechanism of reactive oxygen species in exercise | He F, Li J, Chuang CC, Zuo L | 2016 | Duplicate |
| Redox mechanism of reactive oxygen species in exercise | He F, Li J, Chuang CC, Zuo L | 2016 | Not about recovery |
| Regulation of glycogen resynthesis following exercise - dietary considerations | Friedman JE, Neufer PD, Dohm GL. | 1991 | Not about recovery |
| Regulation of glycogen resynthesis following exercise. dietary considerations | Friedman JE, Neufer PD, Dohm GL. | 1991 | Duplicate |
| Rehabilitation after anterior cruciate ligament reconstruction: criteria-based progression through the return-to-sport phase | Myer GD, Paterno MV, Ford KR, Quatman CE, Hewett TE. | 2016 | Not about recovery |
| Rehabilitation following surgery for lumbar spinal stenosis | McGregor AH, Probyn K, Cro S, Doré CJ, Burton AK, Balagué F, Pincus T, Fairbank J. | 2013 | Focused on disease |
| Rehabilitation for distal radial fractures in adults | Handoll HH, Elliott J. | 2015 | Focused on disease |
| Rehabilitation for hamstring injuries | Mason DL, Dickens VA, Vail A. | 2012 | Not about recovery |
| Rehabilitation in the intensive care unit | Rochester CL. | 2009 | Duplicate |
| Rehabilitation in the intensive care unit | Rochester CL. | 2009 | Not about recovery |
| Rehabilitation nutrition for injury recovery of athletes: the role of macronutrient intake | Papadopoulou SK. | 2020 | Duplicate |
| Rehabilitation nutrition for injury recovery of athletes: the role of macronutrient intake | Papadopoulou SK. | 2020 | Not about recovery |
| Rehabilitation on effort of low back pain. functional restoration programs | Poiraudeau S, Rannou F, MM LC, Boutron I, Revel M. | 2004 | Not about recovery |
| Relation between exercise performance and blood storage condition and storage time in autologous blood doping | Seeger B, Grau M. | 2021 | Not about recovery |
| Relationship of carbohydrate intake during a single-stage one-day ultra-trail race with fatigue outcomes and gastrointestinal problems: a systematic review | Arribalzaga S, Viribay A, Calleja-González J, Fernández-Lázaro D, Castañeda-Babarro A, Mielgo-Ayuso J. | 2021 | Not about recovery |
| Repeated-sprint ability - part ii: recommendations for training | Bishop D, Girard O, Mendez-Villanueva A. | 2011 | Not about recovery |
| Replicability of physical exercise interventions in lung transplant recipients; a systematic review | Knols RH, Fischer N, Kohlbrenner D, Manettas A, De Bruin ED. | 2018 | Focused on disease |
| Resistance exercise training in patients with heart failure | Volaklis KA, Tokmakidis SP. | 2005 | Duplicate |
| Resistance exercise training in patients with heart failure | Volaklis KA, Tokmakidis SP. | 2005 | Not about recovery |
| Resistance training in hypoxia as a new therapeutic modality for sarcopenia-a narrative review | Jung WS, Kim SW, Kim JW, Park HY. | 2021 | Not about recovery |
| Resistance training, antioxidant status, and antioxidant supplementation | Ismaeel A, Holmes M, Papoutsi E, Panton L, Koutakis P. | 2019 | Duplicate |
| Resistance training, antioxidant status, and antioxidant supplementation | Ismaeel A, Holmes M, Papoutsi E, Panton L, Koutakis P. | 2019 | Not about recovery |
| Resistance, resilience, and community dynamics in mediterranean-climate streams | Hershkovitz Y, Gasith A. | 2013 | Not about recovery |
| Respiratory muscle failure | Rochester DF, Arora NS. | 1983 | Focused on physiology |
| Respiratory muscle fatigue during exercise: implications for performance | Johnson BD, Aaron EA, Babcock MA, Dempsey JA. | 1996 | Not about recovery, Focused on physiology |
| Respiratory muscle training for tetraplegic patients: a literature review | Stiller K, Huff N. | 1999 | Not about recovery |
| Respiratory muscle training in children and adults with neuromuscular disease | Silva IS, Pedrosa R, Azevedo IG, Forbes AM, Fregonezi GA, Junior ME, Lima SR, Ferreira GM. | 2019 | Focused on disease |
| Responses of the cardiac transplant patient to exercise and training | Shephard RJ. | 1992 | Focused on disease |
| Responses to acute exercise and training after cardiac transplantation - a review | Shephard RJ. | 1991 | Not about recovery |
| Responses to acute exercise and training after cardiac transplantation: a review | Shephard RJ. | 1991 | Duplicate |
| Rest interval between sets in strength training | Freitas de Salles B, Simao R, Miranda F, da Silva Novaes J, Lemos A, Willardson JM. | 2009 | Not about recovery |
| Restoration of muscle glycogen and functional capacity: role of post-exercise carbohydrate and protein co-ingestion | Alghannam AF, Gonzalez JT, Betts JA. | 2018 | Not a systematic review or meta-analysis |
| Return to sport in athletes with midportion achilles tendinopathy: a qualitative systematic review regarding definitions and criteria | Habets B, van den Broek AG, Huisstede BMA, Backx FJG, van Cingel REH. | 2018 | Duplicate |
| Return to sport in athletes with midportion achilles tendinopathy: a qualitative systematic review regarding definitions and criteria | Habets B, van den Broek AG, Huisstede BMA, Backx FJG, van Cingel REH. | 2018 | Focused on disease |
| Review glucose plus fructose ingestion for post-exercise recovery-greater than the sum of its parts? | Gonzalez JT, Fuchs CJ, Betts JA, Van Loon LJ. | 2017 | Not a systematic review or meta-analysis |
| Review of clinical effects and presumed mechanism of action of the french oak wood extract robuvit | Weichmann F, Avaltroni F, Burki C. | 2021 | Not about recovery |
| Review of physical fitness, physiological demands and performance characteristics of jockeys | Legg KA, Cochrane DJ, Gee EK, Rogers CW. | 2021 | Not about recovery |
| Right heart structural and functional remodeling in athletes | D'Andrea A, La Gerche A, Golia E, Teske AJ, Bossone E, Russo MG, Calabrò R, Baggish AL. | 2015 | Not about recovery, Focused on physiology |
| Right ventricular changes in highly trained athletes: between physiology and pathophysiology | D'Andrea A, La Gerche A, Golia E, Teske AJ, Bossone E, Russo MG, Calabrò R, Baggish AL. | 2015 | Focused on physiology |
| Robot-assisted training for people with spinal cord injury: a meta-analysis | Cheung EYY, Ng TKW, Yu KKK, Kwan RLC, Cheing GLY. | 2017 | Duplicate |
| Robot-assisted training for people with spinal cord injury: a meta-analysis | Cheung EYY, Ng TKW, Yu KKK, Kwan RLC, Cheing GLY. | 2017 | Focused on disease |
| Role of physical exercise for improving posttraumatic nerve regeneration | Armada-da-Silva PA, Pereira C, Amado S, Veloso AP. | 2013 | Duplicate |
| Role of physical exercise for improving posttraumatic nerve regeneration | Armada-da-Silva PA, Pereira C, Amado S, Veloso AP. | 2013 | Not about recovery |
| Role of sodium in fluid homeostasis with exercise | Sharp RL. | 2006 | Duplicate |
| Role of sodium in fluid homeostasis with exercise | Sharp RL. | 2006 | Not about recovery |
| Rowing injuries in elite athletes: a review of incidence with risk factors and the role of biomechanics in its management | Arumugam S, Ayyadurai P, Perumal S, Janani G, Dhillon S, Thiagarajan KA. | 2020 | Duplicate |
| Rowing injuries in elite athletes: a review of incidence with risk factors and the role of biomechanics in its management | Arumugam S, Ayyadurai P, Perumal S, Janani G, Dhillon S, Thiagarajan KA. | 2020 | Not about recovery |
| S‐adenosyl methionine (same) for depression in adults | Galizia I, Oldani L, Macritchie K, Amari E, Dougall D, Jones TN, Lam RW, Massei GJ, Yatham LN, Young AH. | 2016 | Focused on disease |
| Sarcopenia - mechanisms and treatments | Jones TE, Stephenson KW, King JG, Knight KR, Marshall TL, Scott WB. | 2009 | Focused on disease |
| Sarcopenia--mechanisms and treatments | Jones TE, Stephenson KW, King JG, Knight KR, Marshall TL, Scott WB. | 2009 | Duplicate |
| School‐based physical activity programs for promoting physical activity and fitness in children and adolescents aged 6 to 18 | Neil-Sztramko SE, Caldwell H, Dobbins M. | 2021 | Not about recovery |
| Scientific contributions of a. v. hill: exercise physiology pioneer | Bassett Jr DR. | 2002 | Focused on physiology |
| Self-healing composites: a state-of-the-art review | Kanu NJ, Gupta E, Vates UK, Singh GK. | 2019 | Not about recovery |
| Sex differences in endurance running | Besson T, Macchi R, Rossi J, Morio CYM, Kunimasa Y, Nicol C, Vercruyssen F, Millet GY. | 2022 | Duplicate |
| Sex differences in endurance running | Besson T, Macchi R, Rossi J, Morio CYM, Kunimasa Y, Nicol C, Vercruyssen F, Millet GY. | 2022 | Not about recovery |
| Sexual activity before competition and athletic performance: a systematic review | Soori M, Mohaghegh S, Hajian M, Abedi Yekta A. | 2017 | Not about recovery |
| Shock waves as a treatment modality for spasticity reduction and recovery improvement in post-stroke adults - current evidence and qualitative systematic review | Dymarek R, Ptaszkowski K, Ptaszkowska L, Kowal M, Sopel M, Taradaj J, Rosińczuk J. | 2020 | Duplicate |
| Shock waves as a treatment modality for spasticity reduction and recovery improvement in post-stroke adults - current evidence and qualitative systematic review | Dymarek R, Ptaszkowski K, Ptaszkowska L, Kowal M, Sopel M, Taradaj J, Rosińczuk J. | 2020 | Not about recovery |
| Short-burst oxygen therapy in chronic obstructive pulmonary disease | O’Neill B, Mac Mahon J, Bradley J. | 2006 | Focused on disease |
| Short-term recovery from prolonged exercise exploring the potential for protein ingestion to accentuate the benefits of carbohydrate supplements | Betts JA, Williams C. | 2010 | Not a systematic review or meta-analysis |
| Sleep hygiene for optimizing recovery in athletes: review and recommendations | Vitale KC, Owens R, Hopkins SR, Malhotra A. | 2019 | Duplicate |
| Sleep hygiene for optimizing recovery in athletes: review and recommendations | Vitale KC, Owens R, Hopkins SR, Malhotra A. | 2019 | Not a systematic review or meta-analysis |
| Spatial cognitive rehabilitation and motor recovery after stroke | Barrett AM, Muzaffar T. | 2014 | Duplicate |
| Spatial cognitive rehabilitation and motor recovery after stroke | Barrett AM, Muzaffar T. | 2014 | Not about recovery |
| Special feature for the olympics: effects of exercise on the immune system: overtraining effects on immunity and performance in athletes | MacKinnon LT. | 2000 | Not about recovery, Focused on physiology |
| Sport science applied to basketball refereeing: a narrative review | Nabli MA, Ben Abdelkrim N, Fessi MS, DeLang MD, Moalla W, Chamari K. | 2019 | Duplicate |
| Sport science applied to basketball refereeing: a narrative review | Nabli MA, Ben Abdelkrim N, Fessi MS, DeLang MD, Moalla W, Chamari K. | 2019 | Not focused on endurance athletes, Not a systematic review or meta-analysis |
| Sports and games for post‐traumatic stress disorder (PTSD) | Lawrence S, De Silva M, Henley R. | 2010 | Not about recovery |
| Squash racquets - a review of physiology and medicine | Locke S, Colquhoun D, Briner M, Ellis L, O'Brien M, Wollstein J, Allen G. | 1997 | Not about recovery |
| Squash racquets. a review of physiology and medicine | Locke S, Colquhoun D, Briner M, Ellis L, O'Brien M, Wollstein J, Allen G. | 1997 | Duplicate |
| Steroidal contraceptives and bone fractures in women: evidence from observational studies | Lopez LM, Chen M, Mullins S, Curtis KM, Helmerhorst FM. | 2015 | Focused on disease |
| Strategies for stroke rehabilitation | Dobkin BH. | 2004 | Not about recovery |
| Strength training for the warfighter | Kraemer WJ, Szivak TK. | 2012 | Not about recovery |
| Submaximal fitness tests in team sports: a theoretical framework for evaluating physiological state | Shushan T, McLaren SJ, Buchheit M, Scott TJ, Barrett S, Lovell R. | 2022 | Duplicate |
| Submaximal fitness tests in team sports: a theoretical framework for evaluating physiological state | Shushan T, McLaren SJ, Buchheit M, Scott TJ, Barrett S, Lovell R. | 2022 | Focused on physiology |
| Supraventricular arrhythmias in athletes: basic mechanisms and new directions | D’Souza A, Trussell T, Morris GM, Dobrzynski H, Boyett MR. | 2019 | Not about recovery |
| Surface neuromuscular electrical stimulation for quadriceps strengthening pre and post total knee replacement | Monaghan B, Caulfield B, O'Mathúna DP. | 2010 | Not about recovery |
| Surgery for patellar tendinopathy (jumper’s knee) | Dan M, Phillips A, Johnston RV, Harris IA. | 2019 | Focused on disease |
| Surgical interventions for treating acute achilles tendon ruptures | Khan RJ, Smith RL. | 2010 | Not about recovery |
| Surgical versus conservative interventions for treating anterior cruciate ligament injuries | Monk AP, Davies LJ, Hopewell S, Harris K, Beard DJ, Price AJ. | 2016 | Focused on disease |
| Surgical versus conservative treatment for acute injuries of the lateral ligament complex of the ankle in adults | Kerkhoffs GM, Handoll HH, de Bie R, Rowe BH, Struijs PA. | 2007 | Focused on disease |
| Surgical versus non-surgical methods for acute achilles tendon rupture: a meta-analysis of randomized controlled trials | Zhou K, Song L, Zhang P, Wang C, Wang W. | 2018 | Focused on disease |
| Surgical versus non‐surgical treatment for acute anterior shoulder dislocation | Handoll HH, Al‐Maiyah MA. | 2004 | Focused on disease |
| Syncope in the young adult and in the athlete: causes and clinical work-up to exclude a life-threatening cardiac disease | D'Ascenzi F, Zorzi A, Sciaccaluga C, Berrettini U, Mondillo S, Brignole M. | 2020 | Focused on disease, Not about recovery |
| Systematic review of high-intensity progressive resistance strength training of the lower limb compared with other intensities of strength training in older adults | Raymond MJ, Bramley-Tzerefos RE, Jeffs KJ, Winter A, Holland AE. | 2013 | Not about recovery |
| Systemic inflammatory response to exhaustive exercise. cytokine kinetics | Suzuki K, Nakaji S, Yamada M, Totsuka M, Sato K, Sugawara K. | 2002 | Focused on physiology |
| Tapering and peaking maximal strength for powerlifting performance: a review | Travis SK, Mujika I, Gentles JA, Stone MH, Bazyler CD. | 2020 | Duplicate |
| Tapering and peaking maximal strength for powerlifting performance: a review | Travis SK, Mujika I, Gentles JA, Stone MH, Bazyler CD. | 2020 | Not about recovery |
| Tart cherry juice in athletes: a literature review and commentary | Vitale KC, Hueglin S, Broad E. | 2017 | Duplicate |
| Tart cherry juice in athletes: a literature review and commentary | Vitale KC, Hueglin S, Broad E. | 2017 | Not a systematic review or meta-analysis |
| Technique for profiling the cycling-induced oxide trapped charge in nand flash memories | Chiu YY, Shirota R. | 2021 | Not about recovery |
| Ten tips to hurdle the injuries and illnesses during major athletics championships: practical recommendations and resources | Edouard P, Richardson A, Murray A, Duncan J, Glover D, Kiss M, Depiesse F, Branco P. | 2019 | Duplicate |
| Ten tips to hurdle the injuries and illnesses during major athletics championships: practical recommendations and resources | Edouard P, Richardson A, Murray A, Duncan J, Glover D, Kiss M, Depiesse F, Branco P. | 2019 | Not about recovery |
| Test-retest reliability of the yo-yo test: a systematic review | Grgic J, Oppici L, Mikulic P, Bangsbo J, Krustrup P, Pedisic Z. | 2019 | Duplicate |
| Test-retest reliability of the yo-yo test: a systematic review | Grgic J, Oppici L, Mikulic P, Bangsbo J, Krustrup P, Pedisic Z. | 2019 | Not about recovery |
| The 'critical power' concept: applications to sports performance with a focus on intermittent high-intensity exercise | Jones AM, Vanhatalo A. | 2017 | Duplicate |
| The 'critical power' concept: applications to sports performance with a focus on intermittent high-intensity exercise | Jones AM, Vanhatalo A. | 2017 | Not about recovery |
| The application of critical power, the work capacity above critical power (w '), and its reconstitution: a narrative review of current evidence and implications for cycling training prescription | Chorley A, Lamb KL. | 2020 | Not about recovery |
| The application of critical power, the work capacity above critical power (w'), and its reconstitution: a narrative review of current evidence and implications for cycling training prescription | Chorley A, Lamb KL. | 2020 | Duplicate |
| The application of lactose in sports nutrition | Odell OJ, Wallis GA. | 2021 | Not a systematic review or meta-analysis |
| The application of mental fatigue research to elite team sport performance: new perspectives | Russell S, Jenkins D, Smith M, Halson S, Kelly V. | 2019 | Not focused on endurance athletes |
| The biology of sex and sport | Bassett AJ, Ahlmen A, Rosendorf JM, Romeo AA, Erickson BJ, Bishop ME. | 2020 | Not about recovery |
| The concept of maximal lactate steady state - a bridge between biochemistry, physiology and sport science | Billat VL, Sirvent P, Py G, Koralsztein JP, Mercier J. | 2003 | Focused on physiology |
| The concept of maximal lactate steady state: a bridge between biochemistry, physiology and sport science | Billat VL, Sirvent P, Py G, Koralsztein JP, Mercier J. | 2003 | Duplicate |
| The development of aerobic and skill assessment in soccer | O’Reilly J, Wong SH. | 2012 | Not about recovery, Not focused on endurance athletes |
| The effect of acute physical exercise on nk-cell cytolytic activity: a systematic review and meta-analysis | Rumpf C, Proschinger S, Schenk A, Bloch W, Lampit A, Javelle F, Zimmer P. | 2021 | Duplicate |
| The effect of acute physical exercise on nk-cell cytolytic activity: a systematic review and meta-analysis | Rumpf C, Proschinger S, Schenk A, Bloch W, Lampit A, Javelle F, Zimmer P. | 2021 | Not about recovery |
| The effect of aging on skeletal-muscle recovery from exercise: possible implications for aging athletes | Fell J, Williams AD. | 2008 | Not about recovery |
| The effect of branched-chain amino acid on muscle damage markers and performance following strenuous exercise: a systematic review and meta-analysis | Doma K, Singh U, Boullosa D, Connor JD. | 2021 | Not about recovery |
| The effect of consuming carbohydrate with and without protein on the rate of muscle glycogen re-synthesis during short-term post-exercise recovery: a systematic review and meta-analysis | Craven J, Desbrow B, Sabapathy S, Bellinger P, McCartney D, Irwin C. | 2021 | Not eligible |
| The effect of endurance training on serum bdnf levels in the chronic post-stroke phase: current evidence and qualitative systematic review | Górna S, Domaszewska K. | 2022 | Duplicate |
| The effect of endurance training on serum bdnf levels in the chronic post-stroke phase: current evidence and qualitative systematic review | Górna S, Domaszewska K. | 2022 | Not about recovery |
| The effect of endurance, resistance training, and supplements on mitochondria and bioenergetics of muscle cells | Bajes HR, Hakooz NM, Dardeer KT, Al-Dujaili EAS. | 2021 | Not about recovery |
| The effect of high volume power training on repeated high-intensity performance and the assessment of repeat power ability: a systematic review | Natera AO, Cardinale M, Keogh JW. | 2020 | Not about recovery |
| The effect of ingesting carbohydrate and proteins on athletic performance: a systematic review and meta-analysis of randomized controlled trials | Kloby Nielsen LL, Tandrup Lambert MN, Jeppesen PB. | 2020 | Duplicate |
| The effect of lingual resistance training interventions on adult swallow function: a systematic review | Smaoui S, Langridge A, Steele CM. | 2020 | Not about recovery |
| The effect of natural or simulated altitude training on high-intensity intermittent running performance in team-sport athletes: a meta-analysis | Hamlin MJ, Lizamore CA, Hopkins WG. | 2018 | Duplicate |
| The effect of natural or simulated altitude training on high-intensity intermittent running performance in team-sport athletes: a meta-analysis | Hamlin MJ, Lizamore CA, Hopkins WG. | 2018 | Not about recovery, Not focused on endurance athletes |
| The effect of supramaximal exercise on the recovery kinetics of lactate | Freund H, Oyono-Enguelle S. | 1991 | Not about recovery, Focused on physiology |
| The effects of acute and chronic exercise of immunoglobulins | Nieman DC, Nehlsen-Cannarella SL. | 1991 | Duplicate |
| The effects of acute and chronic exercise on immunoglobulins | Nieman DC, Nehlsen-Cannarella SL. | 1991 | Not about recovery |
| The effects of acute and chronic exercise on inflammatory markers in children and adults with a chronic inflammatory disease: a systematic review | Ploeger HE, Takken T, de Greef MH, Timmons BW. | 2009 | Duplicate |
| The effects of acute and chronic exercise on inflammatory markers in children and adults with a chronic inflammatory disease: a systematic review | Ploeger HE, Takken T, de Greef MH, Timmons BW. | 2009 | Focused on disease |
| The effects of aerobic exercise on the recovery of walking ability and neuroplasticity in people with multiple sclerosis: a systematic review of animal and clinical studies | Devasahayam AJ, Downer MB, Ploughman M. | 2017 | Focused on disease |
| The effects of cycling using lower limb active passive trainers in people with neurological conditions: a systematic review | Barclay A, Gray SR, Paul L, Rooney S. | 2022 | Not about recovery |
| The effects of dietary protein supplementation on acute changes in muscle protein synthesis and longer-term changes in muscle mass, strength, and aerobic capacity in response to concurrent resistance and endurance exercise in healthy adults: a systematic review | Hartono FA, Martin-Arrowsmith PW, Peeters WM, Churchward-Venne TA. | 2022 | Duplicate |
| The effects of dietary protein supplementation on acute changes in muscle protein synthesis and longer-term changes in muscle mass, strength, and aerobic capacity in response to concurrent resistance and endurance exercise in healthy adults: a systematic review | Hartono FA, Martin-Arrowsmith PW, Peeters WM, Churchward-Venne TA. | 2022 | Not about recovery, Not focused on endurance athletes |
| The effects of intermittent pneumatic compression on the reduction of exercise-induced muscle damage in endurance athletes: a critically appraised topic | Stedge HL, Armstrong K. | 2021 | Not about recovery |
| The effects of menstrual cycle phase on elite athlete performance: a critical and systematic review | Meignié A, Duclos M, Carling C, Orhant E, Antero J. | 2021 | Not about recovery |
| The effects of mental fatigue on sport-related performance | Pageaux B, Lepers R. | 2018 | Not about recovery |
| The effects of physical prehabilitation in elderly patients undergoing colorectal surgery: a systematic review | Bruns ER, van den Heuvel B, Buskens CJ, Van Duijvendijk P, Festen S, Wassenaar EB, Van Der Zaag ES, Bemelman WA, van Munster BC. | 2016 | Focused on disease |
| The effects of regular cold-water immersion use on training-induced changes in strength and endurance performance: a systematic review with meta-analysis | Malta ES, Dutra YM, Broatch JR, Bishop DJ, Zagatto AM. | 2021 | Duplicate |
| The effects of repeated-sprint training on field-based fitness measures: a meta-analysis of controlled and non-controlled trials | Taylor J, Macpherson T, Spears I, Weston M. | 2015 | Duplicate |
| The effects of repeated-sprint training on field-based fitness measures: a meta-analysis of controlled and non-controlled trials | Taylor J, Macpherson T, Spears I, Weston M. | 2015 | Not about recovery |
| The effects of sports drinks during high-intensity exercise on the carbohydrate oxidation rate among athletes: a systematic review and meta-analysis | Li X, Wang W, Wei C. | 2020 | Not about recovery |
| The effects of training, muscle damage and fatigue on running economy | Burgess TL, Lambert MI. | 2010 | Not about recovery |
| The emerging role of glutamine as an indicator of exercise stress and overtraining | Rowbottom DG, Keast D, Morton AR. | 1996 | Not about recovery |
| The epidemiology, risk factors, and nonsurgical treatment of injuries related to endurance running | Raghunandan A, Charnoff JN, Matsuwaka ST. | 2021 | Duplicate |
| The epidemiology, risk factors, and nonsurgical treatment of injuries related to endurance running | Raghunandan A, Charnoff JN, Matsuwaka ST. | 2021 | Not about recovery |
| The ergogenic effects of acute carbohydrate feeding on resistance exercise performance: a systematic review and meta-analysis | King A, Helms E, Zinn C, Jukic I. | 2022 | Duplicate |
| The ergogenic effects of acute carbohydrate feeding on resistance exercise performance: a systematic review and meta-analysis | King A, Helms E, Zinn C, Jukic I. | 2022 | Not focused on endurance athletes |
| The impact of a novel gaming reinforcement system on oral intake outcomes in pediatric feeding therapy: a single case study | Budhan J, Scarborough D, Kuren MB. | 2019 | Focused on disease, Not a systematic review or meta-analysis |
| The impact of daytime napping on athletic performance - a narrative review | Botonis PG, Koutouvakis N, Toubekis AG. | 2021 | Not a systematic review or meta-analysis |
| The impact of hyperoxia on human performance and recovery | Sperlich B, Zinner C, Hauser A, Holmberg HC, Wegrzyk J. | 2017 | Duplicate |
| The impact of hyperoxia on human performance and recovery | Sperlich B, Zinner C, Hauser A, Holmberg HC, Wegrzyk J. | 2017 | Not a systematic review or meta-analysis |
| The impact of psychological stress on immune function in the athletic population | Clow A, Hucklebridge F. | 2001 | Not about recovery |
| The importance of exercise in lung cancer treatment | Michaels C. | 2016 | Duplicate |
| The importance of exercise in lung cancer treatment | Michaels C. | 2016 | Focused on disease |
| The importance of fatty acids as nutrients during post-exercise recovery | Lundsgaard AM, Fritzen AM, Kiens B. | 2020 | Duplicate |
| The importance of fatty acids as nutrients during post-exercise recovery | Lundsgaard AM, Fritzen AM, Kiens B. | 2020 | Not a systematic review or meta-analysis |
| The influence of exercise and aging on immune function | Mazzeo RS. | 1994 | Not about recovery |
| The influence of hydration on anaerobic performance: a review | Kraft JA, Green JM, Bishop PA, Richardson MT, Neggers YH, Leeper JD. | 2012 | Duplicate |
| The influence of hydration on anaerobic performance: a review | Kraft JA, Green JM, Bishop PA, Richardson MT, Neggers YH, Leeper JD. | 2012 | Not about recovery |
| The influence of post-exercise cold-water immersion on adaptive responses to exercise: a review of the literature | Broatch JR, Petersen A, Bishop DJ. | 2018 | Duplicate |
| The influence of post-exercise cold-water immersion on adaptive responses to exercise: a review of the literature | Broatch JR, Petersen A, Bishop DJ. | 2018 | Not focused on endurance athletes |
| The intriguing role of histamine in exercise responses | Luttrell MJ, Halliwill JR. | 2017 | Not about recovery |
| The na+, k+ pump and muscle excitability | Clausen T, Nielsen OB, Harrison AP, Flatman JA, Overgaard K. | 1998 | Not about recovery |
| The physiological demands of table tennis: a review | Kondrič M, Zagatto AM, Sekulić D. | 2013 | Focused on physiology |
| The physiology of deep-water running | Reilly T, Dowzer CN, Cable NT. | 2003 | Focused on physiology |
| The physiology of rock climbing | Giles LV, Rhodes EC, Taunton JE. | 2006 | Duplicate |
| The physiology of rock climbing | Giles LV, Rhodes EC, Taunton JE. | 2006 | Focused on physiology |
| The potential for high-intensity interval training to reduce cardiometabolic disease risk | Kessler HS, Sisson SB, Short KR. | 2012 | Not about recovery |
| The preventive and therapeutic role of physical activity in knee osteoarthritis | Restuccia R, Ruggieri D, Magaudda L, Talotta R. | 2022 | Duplicate |
| The preventive and therapeutic role of physical activity in knee osteoarthritis | Restuccia R, Ruggieri D, Magaudda L, Talotta R. | 2022 | Not about recovery |
| The psychology of ultra-marathon runners: a systematic review | Roebuck GS, Fitzgerald PB, Urquhart DM, Ng SK, Cicuttini FM, Fitzgibbon BM. | 2018 | Not about recovery |
| The relationship between aerobic fitness and recovery from high intensity intermittent exercise | Tomlin DL, Wenger HA. | 2001 | Not about recovery |
| The resilience of the size principle in the organization of motor unit properties in normal and reinnervated adult skeletal muscles | Gordon T, Thomas CK, Munson JB, Stein RB. | 2004 | Not about recovery |
| The role of energy availability in mammalian hibernation: a cost-benefit approach | Humphries MM, Thomas DW, Kramer DL. | 2003 | Not about recovery |
| The role of physical arm function and demographic disparities in breast cancer survivors' ability to return to work | Vidt ME, Heitzenrater J, Dodge D, Potochny J, Widders K, Paulishak M, Ravnic D, Henry CR, Green M, Kass R, Schmitz KH. | 2022 | Duplicate |
| The role of physical arm function and demographic disparities in breast cancer survivors' ability to return to work | Vidt ME, Heitzenrater J, Dodge D, Potochny J, Widders K, Paulishak M, Ravnic D, Henry CR, Green M, Kass R, Schmitz KH. | 2022 | Focused on disease |
| The role of post-exercise nutrient administration on muscle protein synthesis and glycogen synthesis | Poole C, Wilborn C, Taylor L, Kerksick C. | 2010 | Focused on physiology |
| The role of the nervous system in neuromuscular fatigue induced by ultra-endurance exercise | Millet GY, Martin V, Temesi J. | 2018 | Duplicate |
| The role of the nervous system in neuromuscular fatigue induced by ultra-endurance exercise | Millet GY, Martin V, Temesi J. | 2018 | Focused on physiology |
| The role of tryptophan in fatigue in different conditions of stress | Castell LM, Yamamoto T, Phoenix J, Newsholme EA. | 1999 | Not about recovery, Focused on physiology |
| The science of cycling - physiology and training - part 1 | Faria EW, Parker DL, Faria IE. | 2005 | Not about recovery |
| The training and development of elite sprint performance: an integration of scientific and best practice literature | Haugen T, Seiler S, Sandbakk Ø, Tønnessen E. | 2019 | Duplicate |
| The training and development of elite sprint performance: an integration of scientific and best practice literature | Haugen T, Seiler S, Sandbakk Ø, Tønnessen E. | 2019 | Not about recovery |
| The transition period in soccer: a window of opportunity | Silva JR, Brito J, Akenhead R, Nassis GP. | 2016 | Not focused on endurance athletes |
| The use of acute exercise interventions as game day priming strategies to improve physical performance and athlete readiness in team-sport athletes: a systematic review | Mason B, McKune A, Pumpa K, Ball N. | 2020 | Duplicate |
| The use of acute exercise interventions as game day priming strategies to improve physical performance and athlete readiness in team-sport athletes: a systematic review | Mason B, McKune A, Pumpa K, Ball N. | 2020 | Not focused on endurance athletes |
| The use of aerobic exercise training in improving aerobic capacity in individuals with stroke: a meta-analysis | Pang MY, Eng JJ, Dawson AS, Gylfadóttir S. | 2006 | Focused on disease |
| The use of carbohydrates during exercise as an ergogenic aid | Cermak NM, van Loon LJ. | 2013 | Duplicate |
| The use of carbohydrates during exercise as an ergogenic aid | Cermak NM, van Loon LJ. | 2013 | Not about recovery |
| The yo-yo intermittent recovery test : a useful tool for evaluation of physical performance in intermittent sports | Bangsbo J, Iaia FM, Krustrup P. | 2008 | Not about recovery |
| The yo-yo intermittent tests: a systematic review and structured compendium of test results | Schmitz B, Pfeifer C, Kreitz K, Borowski M, Faldum A, Brand SM. | 2018 | Duplicate |
| The yo-yo intermittent tests: a systematic review and structured compendium of test results | Schmitz B, Pfeifer C, Kreitz K, Borowski M, Faldum A, Brand SM. | 2018 | Not about recovery |
| The young athlete: some physiological considerations | Bar-Or O. | 1995 | Focused on physiology |
| Therapeutic ultrasound for acute ankle sprains | van den Bekerom MP, van der Windt DA, Ter Riet G, van der Heijden GJ, Bouter LM. | 2011 | Focused on disease |
| Time to adapt exercise training regimens in pulmonary rehabilitation--a review of the literature | Lee AL, Holland AE. | 2014 | Not about recovery |
| Time-motion analysis and physiological data of elite under-19-year-old basketball players during competition | Ben Abdelkrim N, El Fazaa S, El Ati J. | 2007 | Not about recovery, Not focused on endurance athletes |
| Timing and method of increased carbohydrate intake to cope with heavy training, competition and recovery | Coyle EF. | 1991 | Not a systematic review or meta-analysis |
| To nap or not to nap? a systematic review evaluating napping behavior in athletes and the impact on various measures of athletic performance | Lastella M, Halson SL, Vitale JA, Memon AR, Vincent GE. | 2021 | Not eligible |
| Topical nsaids for acute musculoskeletal pain in adults | Derry S, Moore RA, Gaskell H, McIntyre M, Wiffen PJ. | 2015 | Focused on disease |
| Training and overtraining: an overview and experimental results in endurance sports | Lehmann MJ, Lormes W, Opitz-Gress A, Steinacker JM, Netzer N, Foster C, Gastmann U. | 1997 | Duplicate |
| Training and overtraining: an overview and experimental results in endurance sports | Lehmann MJ, Lormes W, Opitz-Gress A, Steinacker JM, Netzer N, Foster C, Gastmann U. | 1997 | Not about recovery |
| Training considerations for optimising endurance development: an alternate concurrent training perspective | Doma K, Deakin GB, Schumann M, Bentley DJ. | 2019 | Duplicate |
| Training considerations for optimising endurance development: an alternate concurrent training perspective | Doma K, Deakin GB, Schumann M, Bentley DJ. | 2019 | Not about recovery |
| Training in hypoxia and its effects on skeletal muscle tissue | Hoppeler H, Klossner S, Vogt M. | 2008 | Not about recovery, Focused on physiology |
| Training intensity distribution, training volume, and periodization models in elite swimmers: a systematic review | González-Ravé JM, Hermosilla F, González-Mohíno F, Casado A, Pyne DB. | 2021 | Not about recovery |
| Training principles and issues for ultra-endurance athletes | Zaryski C, Smith DJ. | 2005 | Not about recovery |
| Training techniques to improve endurance exercise performances | Kubukeli ZN, Noakes TD, Dennis SC. | 2002 | Duplicate |
| Training techniques to improve endurance exercise performances | Kubukeli ZN, Noakes TD, Dennis SC. | 2002 | Not about recovery |
| Trajectories of depressive symptoms for bereaved family members of chronically ill patients: a systematic review | Kuo SC, Sun JL, Tang ST. | 2017 | Duplicate |
| Trajectories of depressive symptoms for bereaved family members of chronically ill patients: a systematic review | Kuo SC, Sun JL, Tang ST. | 2017 | Not about recovery |
| Transcriptional adaptations following exercise in thoroughbred horse skeletal muscle highlights molecular mechanisms that lead to muscle hypertrophy | McGivney BA, Eivers SS, MacHugh DE, MacLeod JN, O'Gorman GM, Park SD, Katz LM, Hill EW. | 2009 | Not about recovery |
| Transient elevation of triacylglycerol content in the liver: a fundamental component of the acute response to exercise | Henderson GC, Meyer JM. | 2021 | Not about recovery |
| Treadmill training and body weight support for walking after stroke | Moseley AM, Stark A, Cameron ID, Pollock A. | 2005 | Focused on disease |
| Treatment of proximal hamstring ruptures - a systematic review | Harris JD, Griesser MJ, Best TM, Ellis TJ. | 2011 | Not about recovery |
| Ultra-endurance exercise: unanswered questions in redox biology and immunology | Turner JE, Bennett SJ, Bosch JA, Griffiths HR, Aldred S. | 2014 | Focused on physiology |
| Understanding the ecological and evolutionary function of stopover in migrating birds | Schmaljohann H, Eikenaar C, Sapir N. | 2022 | Not about recovery |
| Updated review of the applied physiology of american college football: physical demands, strength and conditioning, nutrition, and injury characteristics of america's favorite game | Fullagar HHK, McCunn R, Murray A. | 2017 | Duplicate |
| Updated review of the applied physiology of american college football: physical demands, strength and conditioning, nutrition, and injury characteristics of america's favorite game | Fullagar HHK, McCunn R, Murray A. | 2017 | Not about recovery |
| Urinary biomarkers of physical activity: candidates and clinical utility | Sampson DL, Broadbent JA, Parker AW, Upton Z, Parker TJ. | 2014 | Not about recovery |
| Urinary excretion of cytokines versus their plasma levels after endurance exercise | Sugama K, Suzuki K, Yoshitani K, Shiraishi K, Kometani T. | 2013 | Focused on physiology |
| Use of heart rate monitors by endurance athletes: lessons from triathletes | O'Toole ML, Douglas PS, Hiller WD. | 1998 | Duplicate |
| Use of heart rate monitors by endurance athletes: lessons from triathletes | O'Toole ML, Douglas PS, Hiller WD. | 1998 | Focused on physiology |
| Use of loaded conditioning activities to potentiate middle- and long-distance performance: a narrative review and practical applications | Blagrove RC, Howatson G, Hayes PR. | 2019 | Duplicate |
| Use of loaded conditioning activities to potentiate middle- and long-distance performance: a narrative review and practical applications | Blagrove RC, Howatson G, Hayes PR. | 2019 | Not about recovery |
| Usefulness of beta-hydroxy-beta-methylbutyrate (hmb) supplementation in different sports: an update and practical implications | Albert FJ, Morente-Sanchez J, Ortega FB, Castillo MJ, Gutierrez A. | 2015 | Not a systematic review or meta-analysis |
| Usefulness of combining intermittent hypoxia and physical exercise in the treatment of obesity | Urdampilleta A, Gonzalez-Muniesa P, Portillo MP, Martinez JA. | 2012 | Not about recovery |
| Usefulness of β-hydroxy-β-methylbutyrate (hmb) supplementation in different sports: an update and practical implications | Albert FJ, Morente-Sánchez J, Ortega FB, Castillo MJ, Gutiérrez Á. | 2015 | Duplicate |
| Using aerobic exercise to improve health outcomes and quality of life in stroke: evidence-based exercise prescription recommendations | Pang MY, Charlesworth SA, Lau RW, Chung RC. | 2013 | Focused on disease |
| Using field based data to model sprint track cycling performance | Ferguson HA, Harnish C, Chase JG. | 2021 | Duplicate |
| Using field based data to model sprint track cycling performance | Ferguson HA, Harnish C, Chase JG. | 2021 | Not about recovery |
| Valproate for acute mania | Jochim J, Rifkin‐Zybutz RP, Geddes J, Cipriani A | 2019 | Focused on disease |
| Vitamin supplementation benefits in master athletes | Brisswalter J, Louis J. | 2014 | Not a systematic review or meta-analysis |
| Warm up ii - performance changes to structure the warm following active warm up and how up | Bishop D. | 2003 | Not about recovery |
| Warm up ii: performance changes following active warm up and how to structure the warm up | Bishop D. | 2003 | Not about recovery |
| Water conservation and plant survival strategies of rhizobacteria under drought stress | Khan N, Ali S, Tariq H, Latif S, Yasmin H, Mehmood A, Shahid MA. | 2020 | Not about recovery |
| Weight-loss and weight cycling in amateur wrestlers - implications for performance and resting metabolic-rate | Horswill CA. | 1993 | Not about recovery |
| What are the dietary protein requirements of physically active individuals? new evidence on the effects of exercise on protein utilization during post-exercise recovery | Fielding RA, Parkington J. | 2002 | Not a systematic review or meta-analysis |
| What are the physiological mechanisms for post-exercise cold water immersion in the recovery from prolonged endurance and intermittent exercise? | Ihsan M, Watson G, Abbiss CR. | 2016 | Duplicate |
| What are the physiological mechanisms for post-exercise cold water immersion in the recovery from prolonged endurance and intermittent exercise? | Ihsan M, Watson G, Abbiss CR. | 2016 | Not a systematic review or meta-analysis |
| What does evidence tell us about the use of gait robotic devices in patients with multiple sclerosis? a comprehensive systematic review on functional outcomes and clinical recommendations | Calabrò RS, Cassio A, Mazzoli D, Andrenelli E, Bizzarini E, Campanini I, Carmignano SM, Cerulli S, Chisari C, Colombo V, Dalise S, Fundarò C, Gazzotti V, Mazzoleni D, Mazzucchelli M, Melegari C, Merlo A, Stampacchia G, Boldrini P, Mazzoleni S, Posteraro F, Benanti P, Castelli E, Draicchio F, Falabella V, Galeri S, Gimigliano F, Grigioni M, Mazzon S, Molteni F, Petrarca M, Picelli A, Senatore M, Turchetti G, Morone G, Bonaiuti D and Italian Consensus Conference on Robotics in Neurorehabilitation (CICERONE). | 2021 | Duplicate |
| What does evidence tell us about the use of gait robotic devices in patients with multiple sclerosis? a comprehensive systematic review on functional outcomes and clinical recommendations | Calabrò RS, Cassio A, Mazzoli D, Andrenelli E, Bizzarini E, Campanini I, Carmignano SM, Cerulli S, Chisari C, Colombo V, Dalise S, Fundarò C, Gazzotti V, Mazzoleni D, Mazzucchelli M, Melegari C, Merlo A, Stampacchia G, Boldrini P, Mazzoleni S, Posteraro F, Benanti P, Castelli E, Draicchio F, Falabella V, Galeri S, Gimigliano F, Grigioni M, Mazzon S, Molteni F, Petrarca M, Picelli A, Senatore M, Turchetti G, Morone G, Bonaiuti D and Italian Consensus Conference on Robotics in Neurorehabilitation (CICERONE). | 2021 | Not about recovery |
| What makes an endurance athlete world-class? not simply a physiological conundrum | Myburgh KH. | 2003 | Duplicate |
| What makes an endurance athlete world-class? not simply a physiological conundrum | Myburgh KH. | 2003 | Not about recovery |
| Whole skeletal-muscle transplantation - mechanisms responsible for functional deficits - review | Faulkner JA, Carlson BM, Kadhiresan VA. | 1994 | Not about recovery |
| Winning the war against icu-acquired weakness: new innovations in nutrition and exercise physiology | Wischmeyer PE, San-Millan, I. | 2015 | Not about recovery |
| Yoga for stroke rehabilitation | Lawrence M, Celestino Junior FT, Matozinho HH, Govan L, Booth J, Beecher J. | 2017 | Duplicate |
| Yoga for stroke rehabilitation | Lawrence M, Celestino Junior FT, Matozinho HH, Govan L, Booth J, Beecher J. | 2017 | Not about recovery |
